# Dimethylnonacethrene – *en route* to a magnetic switch†

**DOI:** 10.1039/d3cc01301d

**Published:** 2023-05-17

**Authors:** Daniel Čavlović, Olivier Blacque, Ivo Krummenacher, Holger Braunschweig, Prince Ravat, Michal Juríček

**Affiliations:** a Department of Chemistry, University of Zurich Winterthurerstrasse 190 Zurich 8057 Switzerland michal.juricek@chem.uzh.ch; b Institute of Inorganic Chemistry, Julius Maximilian University of Würzburg Am Hubland Würzburg 97074 Germany; c Institute of Organic Chemistry, Julius Maximilian University of Würzburg Am Hubland Würzburg 97074 Germany princekumar.ravat@uni-wuerzburg.de; d Department of Chemistry, University of Basel St. Johanns-Ring 19 Basel 4056 Switzerland

## Abstract

Dimethylnonacethrene is the first derivative of the cethrene family that is energetically more stable than the product of its electrocyclic ring closure. Compared to the shorter homologue dimethylcethrene, the new system is EPR-active, because of a significantly lowered singlet–triplet gap, and displays remarkable stability. Our results suggest that adjustment of the steric bulk in the fjord region can enable realisation of diradicaloid-based magnetic photoswitches.

## Abstract 2

We report the first isolable cethrene derivative—dimethylnonacethrene. In contrast to all other reported cethrenes, this derivative is lower in energy compared to its electrocyclised product and has the lowest singlet–triplet gap in the series.

## Graphical abstract

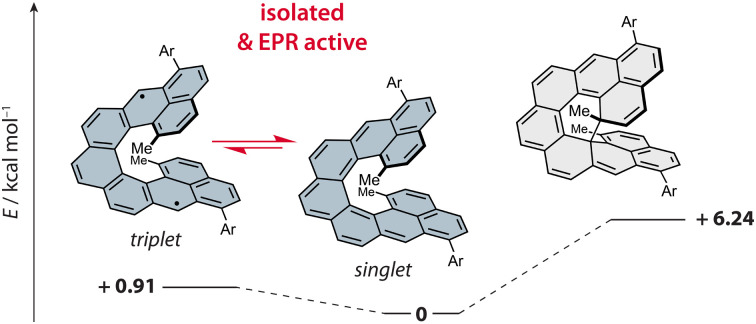

Open-shell molecular fragments of graphene,^1–4^ a subclass of polycyclic aromatic hydrocarbons (PAHs) that display magnetic properties, hold prospects of making light-weight and flexible materials, and eliminating the use of rare earth elements in electronic devices.^5–9^ An attractive opportunity to modulate magnetic properties of a device on demand are PAH-based magnetic photoswitches that operate under ambient conditions.^10–13^ There are different ways of tuning the magnetic properties in PAHs, for example, by variation of substituents^14,15^ or chemical treatment,^16,17^ but the ability to reversibly turn magnetic properties off by using light remains a design challenge.^13,18,19^

To control magnetic properties in such systems by means of secondary orbital, or through-space, interactions,^20–22^ Juríček and coworkers developed a family of C-shaped helical PAHs named cethrenes (Fig. 1).^21,23,24^ This class of compounds display either a singlet diradicaloid or a triplet diradical ground state, and a varying degree of cofacial overlap between the terminal phenalenyl units.^25^ Using a diphenyl derivative of the first homologue (Fig. 1A), we demonstrated^21^ that through-space interactions (bonding within the HOMO, antibonding within the LUMO) lower the energy gap between the singlet ground state and the triplet excited state (Δ*E*_ST_ ∼ 6 kcal mol^−1^) compared to the planar Z-shaped isomer heptazethrene (Δ*E*_ST_ ∼ 9 kcal mol^−1^). As a result, an electron paramagnetic resonance (EPR) signal originating from the thermally populated triplet state of cethrene is observed, while heptazethrene is EPR silent.^26^

**Fig. 1:**
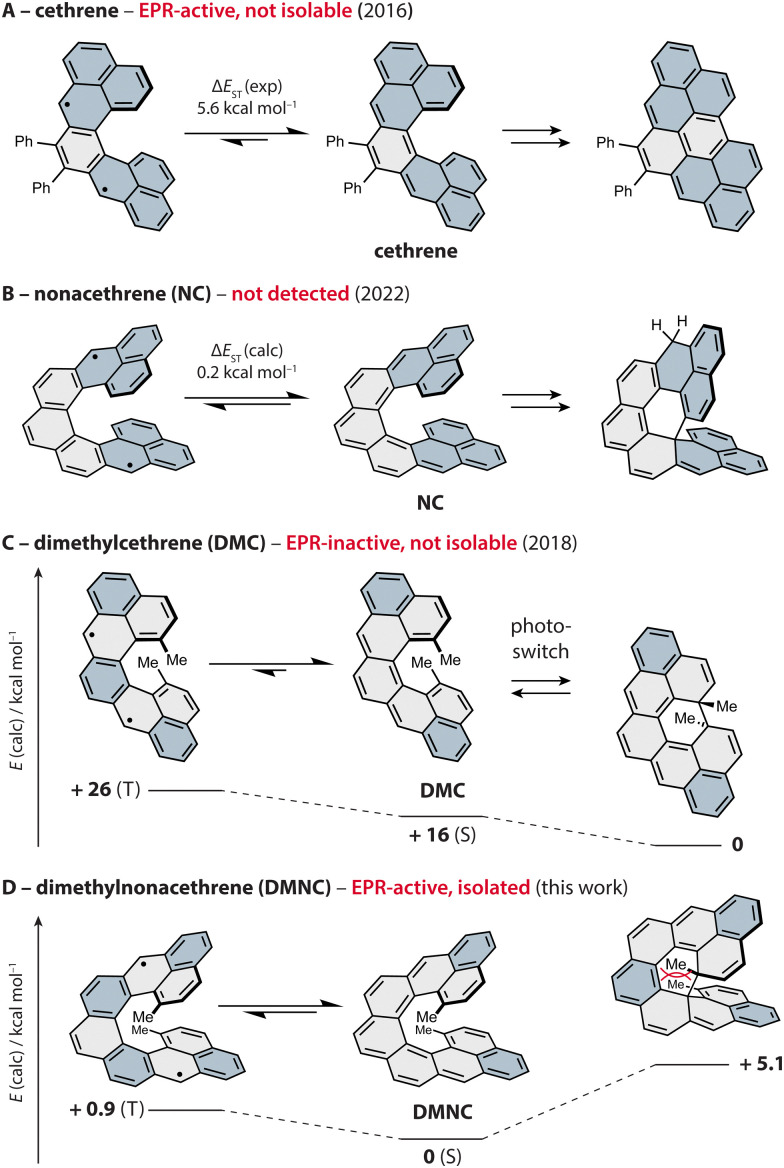
Progress in the development of magnetic photoswitches based on cethrenes, shown as thermal equilibrium between the singlet ground state (S, middle) and triplet excited state (T, left); Δ*E*_ST_ from ref. 21 (A), 38 (B) and 34 (C). The isolated products of electrocyclisation are shown on the right, except for DMNC (product not observed). The phenalenyl units (A, B) and Clar sextets (C, D) are highlighted in blue. Relative energies for DMC (0, +16) and DMNC (all) were obtained *via* DFT (ωB97DX/Def2SVP) and CAS(2,2) and the energies of the singlet ground states *via* broken-symmetry calculations.

During these investigations, we found that cethrene undergoes a formally symmetry-forbidden thermal 6π electrocyclic ring closure^27–31^ with a surprisingly low activation barrier,^32^ followed by an oxidation to a flat hydrocarbon. To prevent the irreversible oxidation step and benefit from the photochemical electrocyclic process, methyl groups were installed in the fjord region, yielding a reversible chiral photoswitch^33,34^ (Fig. 1C). Unfortunately, the improved stability came at the cost of the EPR response, as the installment of the methyl substituents led to an increase of Δ*E*_ST_ by ∼ 4 kcal mol^−1^ compared to the system without methyl groups. In addition to the electronic effect, the methyl groups increase Δ*E*_ST_ by increasing the helical twist which causes weakening of the through-space orbital overlap along with a significantly improved configurational stability.^35,36^ Despite the absence of the EPR response, we postulated that the working principle of dimethylcethrene could be applied in the design of an all-organic magnetic photoswitch, a concept that was successfully realised by Dumele and coworkers in a system operating at cryogenic temperatures.^37^

To achieve a chiral magnetic photoswitch that would function at ambient temperature, we pursued the third homologue in the series, nonacethrene^38^ (Fig. 1B), featuring a helicene backbone extended by two rings compared to cethrene. Even though the necessity to functionalise the fjord region was thought to be alleviated, we could only observe what appears to be the product of a 6π electrocyclisation of nonacethrene followed by a 1,5-hydrogen shift. In this work, we pursued the next step toward realisation of a cethrene-based magnetic photoswitch and present dimethylnonacethrene (DMNC, Fig. 1D, left), the first isolable cethrene derivative. In contrast to all other reported cethrenes, DMNC is lower in energy compared to the product of its 6π electrocyclisation, which was not observed even upon light irradiation, and has the lowest experimentally estimated Δ*E*_ST_ in the series (∼ 1 kcal mol^−1^).

The synthetic strategy toward DMNC capitalises on the route developed for nonacethrene^38^ (NC) and employs two photo-chemical steps to build the [7]helicene backbone (Scheme 1). Starting from a commercial 2-bromo-5-methylbenzaldehyde (1), intermediate 2 was prepared in a series of four steps, including Heck coupling with methyl acrylate (*a*), reduction of the double bond and the aldehyde moiety with sodium borohydride and nickel(ii) chloride (*b*), substitution of the benzylic hydroxyl group by a bromide with phosphorus tribromide (*c*) and treatment with triphenylphosphine (*d*) to afford the desired phosphonium salt 2 in an overall 61% yield. Next, a Wittig reaction of 2 with dialdehyde 3^38^ in the presence of sodium methoxide (*e*) gave diester 4 in a 71% yield. Subsequent photocyclodehydrogenation of 4 using a medium-pressure mercury lamp and iodine as an oxidant (*f*) afforded [7]helicene intermediate (9; see the ESI†) in a 75% yield, the structure of which was confirmed by single-crystal X-ray diffraction (SC-XRD; ESI†). Upon hydrolysis with lithium hydroxide (*g*), the diacid intermediate was transformed into the corresponding diacyl chloride (*h*), followed by a Friedel–Crafts acylation mediated by titanium tetrachloride (*i*) to afford the key intermediate 5 in a 73% yield over the three steps (for SC-XRD of 5, see the ESI†).

**Scheme 1:**
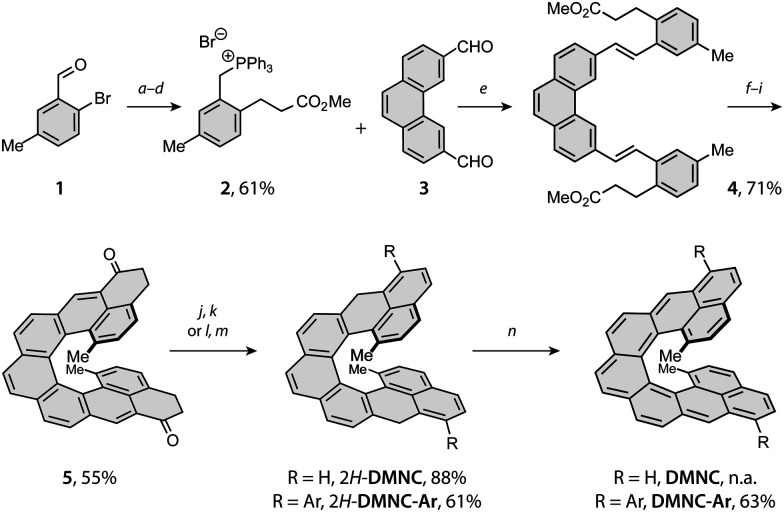
Reaction conditions: (*a*) Pd(OAc)_2_, TBAB, PPh_3_, K_2_CO_3_, methyl acrylate, DMF, 90 °C, 20 h; (*b*) NiCl_2_, NaBH_4_, THF/MeOH 1 : 1, 0 °C to rt, 1 h; (*c*) PBr_3_, Et_2_O, rt, 18 h; (*d*) PPh_3_, toluene, 100 °C, 16 h; (*e*) NaOMe, THF, rt, 20 h; (*f*) *hν*, PO, toluene, rt, 2 h; (*g*) LiOH, THF/H_2_O 10 : 1, 80 °C, 16 h; (*h*) (COCl)_2_, 65 °C, 2 h; (*i*) TiCl_4_, CH_2_Cl_2_, −50 to −20 °C, 6 h; (*j*) NaBH_4_, CH_2_Cl_2_/EtOH 2 : 1, rt, 5 h; (*k*) *p*-TSA, toluene, 90 °C, 5 min; (*l*) ArMgBr, THF, −78 °C to rt, 18 h; (*m*) TFA, CH_2_Cl_2_, rt, 5 min; (*n*) *p*-chloranil, benzene-*d*_6_, 50 °C, 10 min; TBAB = tetrabutylammonium bromide, DMF = *N*,*N*′-dimethylformamide; THF = tetrahydrofuran; rt = room temperature; PO = propylene oxide; *p*-TSA = *para*-toluenesulfonic acid; TFA = trifluoroacetic acid. Ar = 3,5-di-*tert*-butylphenyl.

From 5, first the synthesis of parent DMNC was attempted, where a reduction with sodium borohydride followed by a dehydration with a catalytic amount of *p*-toluenesulfonic acid afforded the dihydro precursor 2*H*-DMNC in an 88% yield over the two steps. The final oxidation step was performed in argon-saturated benzene with *p*-chloranil, yielding an insoluble, EPR-active solid (Fig. S49, ESI†). Because structure identification was hampered on account of limited solubility and the fact that presence of monoradical impurities could not be excluded, solubilising 3,5-di-*tert*-butylphenyl groups were installed *via* a nucleophilic 1,2-addition to the carbonyl groups of 5. These substituents provided also kinetic stabilisation, as they were installed next to the positions that display the highest spin density in the triplet excited state (Table S4, ESI†). After the addition, dehydration occurred either directly or was promoted by adding a drop of trifluoroacetic acid to afford the dihydro precursor 2*H*-DMNC-Ar in form of the most stable isomer featuring two naphthalene and one phenanthrene units. Its structure was supported by SC-XRD (Fig. 2, left). The final step was performed in argon-saturated benzene-*d*_6_ with *p*-chloranil as the oxidant, and the conversion was followed by ^1^H NMR spectroscopy. At room temperature, the conversion was relatively slow but heating to 50 °C led to a full conversion within 10 min, as indicated by a complete disappearance of the signals that belong to the starting material (Fig. S1, ESI†) and a matching *m*/*z* peak in HRMS. No new peaks could be observed, apart from a very broad signal spread over the aromatic region. The desired DMNC-Ar was isolated as a deep-purple solid (*λ*_max_ = 863 nm, Fig. 2, right) after column chromatography in a 63% yield.

**Fig. 2:**
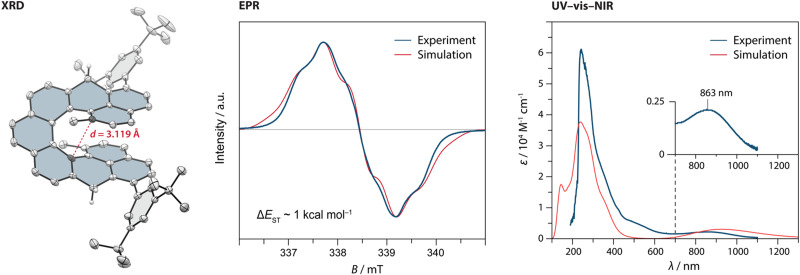
(left) The solid-state structure of 2*H*-DMNC-Ar with the thermal ellipsoids shown at a 50% probability level. The nonacethrene core is highlighted in blue. All hydrogen atoms, except for those of the CH_2_ groups, are omitted for clarity. (centre) Comparison of the experimental and simulated electron paramagnetic resonance (EPR) spectra of a solution of DMNC-Ar (8.7 mM in benzene-*d*_6_) at 298 K. Experimental parameters are shown in Table S8 (ESI†) (right) Comparison of the experimental (at 298 K) and simulated UV-vis-NIR absorption spectra of a solution of DMNC-Ar (CH_2_Cl_2_; TD-DFT/B3LYP/6-31(*d*,*p*)).

DMNC-Ar was studied by EPR spectroscopy as a solid powder, in a solid *o*-terphenyl matrix and in a benzene solution (Fig. 2, centre and Fig. S48, ESI†). In all three cases, a strong but broadened signal was detected without the observation of a forbidden half-field signal. The broadening is presumably the result of several unresolved small hyperfine couplings. In the benzene solution (8.7 mM), a discernible quintet multiplicity is observed, which is in a good qualitative agreement with the simulation (Fig. 2, centre) performed using DFT-calculated hyperfine coupling constants (Tables S4 and S8, ESI†). The shape and the intensity of the signal remained almost unchanged after the benzene sample was kept for a year in a sealed EPR tube, demonstrating that DMNC-Ar is stable in the absence of oxygen and does not undergo 6π electrocyclic ring closure, in contrast to its shorter homologue DMC. Notably, the signal could be detected even after two weeks of the sample being kept in a non-deoxygenated CH_2_Cl_2_ solution as well as using a solution prepared from a solid powder that was stored under ambient conditions for several months. For a comparison, kinetically stabilised nonazethrene has a half-life of 16 h^39^ and nonacene is reported to be stable at room temperature only under anhydrous argon atmosphere.^40^

To determine Δ*E*_ST_, variable-temperature EPR measurements were performed. The intensity of the signal arising from the thermally populated triplet excited state decreased with lowering the temperature, and the fitting of these data using the Bleaney–Bowers equation^41^ (Fig. S51, ESI†) gave a Δ*E*_ST_ estimate of ∼1 kcal mol^−1^, matching well the values (0.3–2.0 kcal mol^−1^) calculated using different methods (Table S5, ESI†). Compared to the flat isomer nonazethrene (5.6 kcal mol^−1^), linear nonacene (5.8 kcal mol^−1^)^39^ as well as non-planar tetrabenzoperylene (3.4 kcal mol^−1^),^42^ Δ*E*_ST_ of DMNC-Ar is significantly smaller, which illustrates the impact of through-space interactions. In addition, this value is smaller than that of the shorter homologue cethrene (5.6 kcal mol^−1^),^32^ which reflects the gain of two (DMNC) *versus* one (cethrene) Clar sextet in the diradical resonance structure (see Fig. 1A, B, and Fig. S58 and Table S9 for discussion of NICS and ACID studies, ESI†). This trend is similar to that for zethrene and its higher homologues.^43,44^ Compared to NC (Δ*E*_ST_ ∼ 0.2 kcal mol^−1^), Δ*E*_ST_ of DMNC-Ar is larger, which manifests the steric effect of the methyl groups leading to the increase of helical twist. For example, the distance between the carbon atoms that display the largest through-space orbital overlap (highlighted in Fig. 2, left) increases from 2.90 (NC; DFT) to 3.09 Å (DMNC-Ar; DFT), or from 2.974(0)^38^ to 3.118(6) Å (XRD; Fig. 2, left) in the case of dihydro precursors, as observed^34^ previously for cethrene and DMC.

To photochemically induce 6π electrocyclisation, UV-vis-near-infrared (NIR) absorption studies were performed in CH_2_Cl_2_. DMNC-Ar displays a broad absorption band in the NIR region (Fig. 2, right; *λ*_max_ = 863 nm) characteristic of diradicaloid compounds with small HOMO–LUMO gaps. Irradiation of the sample with LED light sources (254, 365, 505, 740 and 800 nm) for several minutes at room or cryogenic (77 K, 2-methyl tetrahydrofuran matrix) temperature did not result in any change of the absorption spectrum. DFT calculations were performed to calculate the relative energies of DMNC (the “open” form) and its electrocyclised product (the “closed” form) where three different functionals (PBE0, B3LYP-D3 and ωB97DX) show the same trend: the open form is lower in energy by 5–12 kcal mol^−1^, supporting all experimental observations. This feature is in stark contrast to all other reported cethrenes and results from a destabilising steric effect in the fjord region caused by the methyl groups. This effect is more pronounced in the closed form (∼9 kcal mol^−1^) than in the open form (∼4 kcal mol^−1^), which leads to inversion of the relative energies (for NC, the closure is only slightly exoergic; see Table S8, ESI†). Considering the relatively small energy difference between the open and the closed form of DMNC, these results suggest that either the activation energy of the ring closure is too large or, more likely, the activation energy of the ring opening is too small,^45^ which would result in a short-lived closed form that is undetectable even at cryogenic temperatures.

Our findings illustrate that similarly to planar zethrene analogues, π-extension is an effective tool to decrease Δ*E*_ST_ in helical cethrenes. Methyl substituents in the fjord region prevent reactivity previously observed for nonacethrene and, notably, invert the typical trend of relative energies for cethrenes and their electrocyclised products. Two aryl groups were installed in the periphery of DMNC to provide solubility and to promote kinetic stabilisation. The results of our spectroscopic measurements and computational studies all point toward inverted equilibrium between the open and the closed form, in contrast to all previously reported cethrenes. In addition, an extraordinary stability of the open form without significant decomposition was observed in solution and in the solid state over several months. The closed form was not observed even upon photoirradiation under ambient or cryogenic conditions. The adjustment of the steric bulk in the fjord region represents an opportunity for further optimisation to achieve bistability and a viable strategy to realise a magnetic photoswitch operating at ambient temperature.

## Conflicts of interest

There are no conflicts to declare.

## Supplementary Material

CC-059-D3CC01301D-s001

CC-059-D3CC01301D-s002
