# Supplementary material for: Dimethylnonacethrene – en route to a magnetic switch
Source: Chem Commun (Camb). 2023 May 17;59(50):7743–6. doi: 10.1039/d3cc01301d (PMC10281489; doi:10.1039/d3cc01301d)
Supplement: CC-059-D3CC01301D-s001 [file CC-059-D3CC01301D-s001.pdf]

Electronic Supplementary Material (ESI) for Chemical Communications.
This journal is © The Royal Society of Chemistry 2023

Supplementary Information

Dimethylnonacethrene – *en route* to a magnetic switch

Daniel Čavlović,[†] Olivier Blacque,[†] Ivo Krummenacher,[#] Holger Braunschweig,[#] Prince Ravat,[*,‡,§] and Michal Juríček[*,†,§]

[†]Department of Chemistry, University of Zurich, Winterthurerstrasse 190, 8057 Zurich, Switzerland

[‡]Institute of Inorganic Chemistry, Julius Maximilian University of Würzburg, Am Hubland, 97074 Würzburg, Germany

[#]Institute of Organic Chemistry, Julius Maximilian University of Würzburg, Am Hubland, 97074 Würzburg, Germany

[§]Department of Chemistry, University of Basel, St. Johanns-Ring 19, CH-4056 Basel, Switzerland

Table of Contents

Supporting Figures S1
Synthesis S2
NMR Assignments S7
Copies of NMR Spectra S9
HR-MS Data S25
EPR Spectroscopy S37
UV–vis–NIR Spectroscopy S39
X-Ray Crystallography S40
Quantum Chemical Calculations S46
Cartesian Coordinates S53
References S64

Supporting Figures

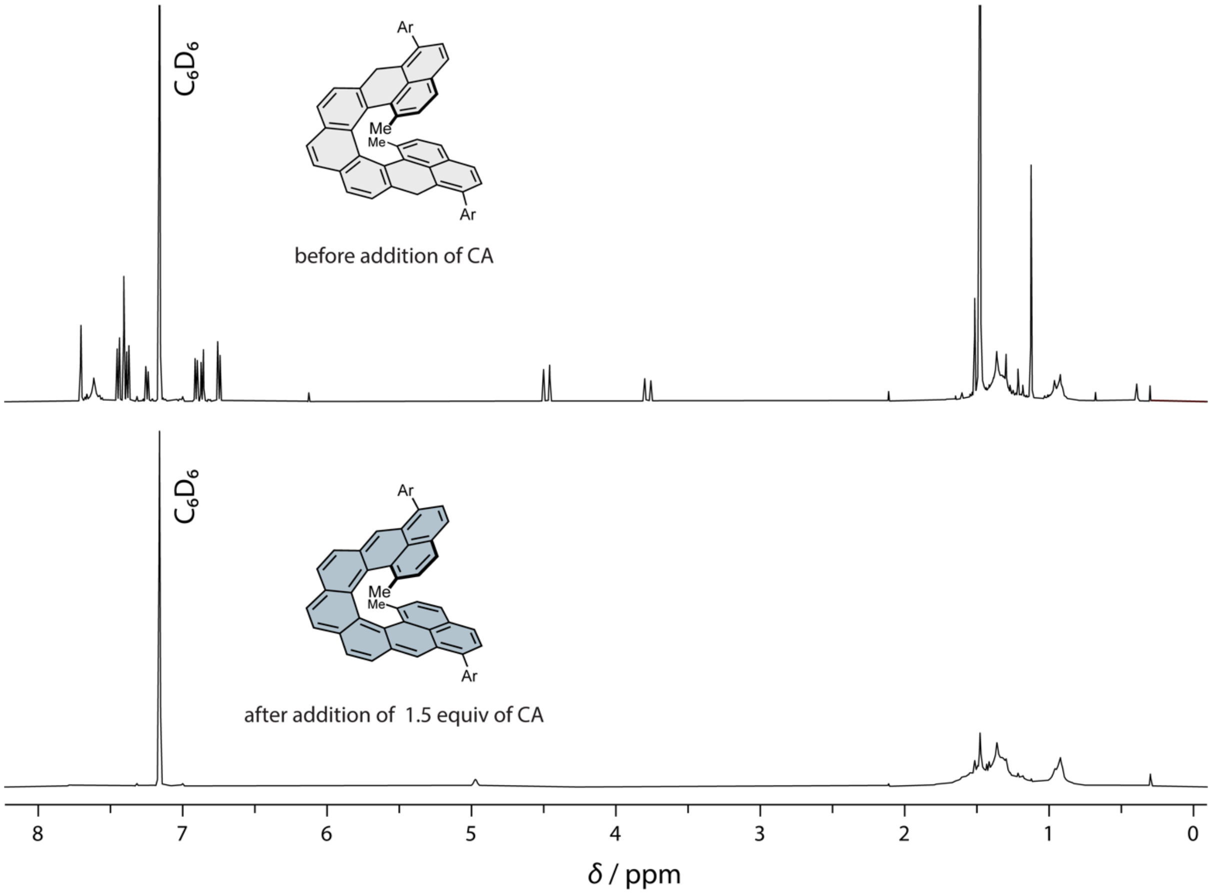

Figure S1: Comparison of ^{1}H NMR (400 MHz, 298 K, C_6D_6) spectra of 2*H*-**DMNC-Ar** before (top) and after (bottom) the addition of 1.5 equivalents of *p*-chloranil (CA).

Synthesis

Materials and Instrumentation. All chemicals and solvents were purchased from commercial sources and were used without further purification unless stated otherwise. The reactions and experiments that are sensitive to dioxygen were performed using Schlenk techniques and argon-saturated solvents. The NMR experiments were performed on NMR spectrometers operating at 400 or 500 MHz proton frequencies. Standard pulse sequences were used, and the data were processed using twofold zero-filling in the indirect dimension for all 2D experiments. Chemical shifts (δ) are reported in parts per million (ppm) relative to the solvent residual peak (^{1}H and ^{13}C NMR, respectively): $CDCl_3$ (δ = 7.26 and 77.16 ppm) and CD_2Cl_2 (δ = 5.32 and 53.84 ppm).[1] Compound **3** was previously described by our group and synthesized according to the published procedure.[2] The raw NMR and IR data is available free of charge on a public repository Zenodo (DOI:10.5281/zenodo.7733689).

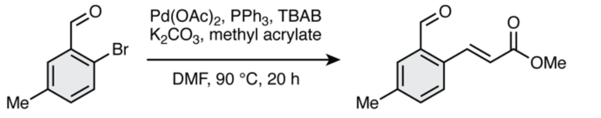

1 **6, 95%**

*Methyl (E)-3-(2-formyl-4-methylphenyl)acrylate (**6**).* A mixture of commercially available 2-bromo-5-methylbenzaldehyde (**1**; 1.00 g, 5.02 mmol, 1.0 equiv.), triphenyl phosphine (0.41 g, 1.56 mmol, 31 mol%), palladium acetate (175 mg, 779 µmol, 16 mol%), potassium carbonate (760 mg, 5.11 mmol, 1.0 equiv.), and tetrabutylammonium bromide (TBAB; 1.64 g, 5.09 mmol, 1.0 equiv.) in DMF (125 mL) was deoxygenated by passing nitrogen through the mixture under sonication for 30 min. Then, methyl acrylate (1.9 mL, 21 mmol, 4.2 equiv.) was added before the mixture was heated at 90 °C for 20 h under a nitrogen atmosphere. The reaction mixture was poured into water and extracted with CH_2Cl_2. The combined organic layers were washed with aq. HCl (2 M), dried over anhydrous $MgSO_4$, and filtered. After evaporation of the solvents, the residue was passed through a pad of celite using CH_2Cl_2 as an eluent to afford, after evaporation of the solvent, the desired product (972 mg, 95%) as an orange solid (mp 53.8–54.7 °C). ^{1}H NMR (400 MHz, $CDCl_3$, ppm): δ 10.28 (s, 1H), 8.51 (d, J = 15.9 Hz, 1H), 7.68 (d, J = 1.9 Hz, 1H), 7.55 (d, J = 7.9 Hz, 1H), 7.42 (dd, J = 7.9, 1.9 Hz, 1H), 6.36 (d, J = 15.9 Hz, 1H), 3.83 (s, 3H), 2.45 (s, 3H). ^{13}C NMR (101 MHz, $CDCl_3$, ppm): δ 192.1, 167.0, 141.2, 140.6, 134.9, 133.91, 133.89, 132.9, 128.0, 122.0, 52.0, 21.3. IR: 1710, 184, 1432, 1321, 1312, 1285, 1189, 1175, 1156, 1115, 998, 984, 932, 830, 775, 743, 710, 510 cm^{-1}. HRMS (ESI) m/z: $[M + H]^+$ Calcd for $C_{12}H_{13}O_3$ 205.08592; Found 205.08576. The data are in accordance with those reported in the literature.[3]

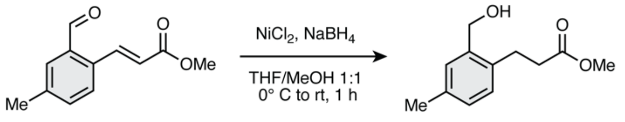

6 **7, >99%**

*Methyl 3-[2-(hydroxymethyl)-4-methylphenyl]propanoate (**7**).* A mixture of methyl (E)-3-(2-formyl-4-methylphenyl)acrylate (**6**; 972 mg, 4.76 mmol, 1.0 equiv.) and nickel chloride hexahydrate (1.13 g, 4.76 mmol, 1.0 equiv.) in THF/MeOH (80 mL, 1:1) was stirred at 0 °C for 15 min before sodium borohydride (505 mg, 13.3 mmol, 2.8 equiv.) was carefully added in three portions. The mixture was stirred for 45 min at room temperature before it was poured into aq. HCl (2 M) and extracted with CH_2Cl_2. The combined organic layers were dried over anhydrous $MgSO_4$ and filtered. After evaporation of the solvents, the residue was passed through a pad of celite using CH_2Cl_2 as an eluent to afford, after evaporation of the solvent, the desired product (992 mg, >99%) as a colorless liquid. ^{1}H NMR (400 MHz, $CDCl_3$, ppm): δ 7.18 (d, J = 2.0 Hz, 1H), 7.10 (d, J = 7.8 Hz, 1H), 7.07 (dd, J = 7.8, 2.0 Hz, 1H), 4.69 (s, 2H), 3.65 (s, 3H), 2.99 (t, J = 7.6 Hz, 2H), 2.68 (t, J = 7.6 Hz, 2H), 2.32 (s, 3H). ^{13}C NMR (101 MHz, $CDCl_3$, ppm): δ 173.9, 138.4, 136.4, 135.8, 130.0, 129.2, 129.0, 63.4, 51.9, 35.5, 26.7, 21.1. IR: 3443, 2950, 2921, 1732, 1436, 1195, 1151, 1027, 990, 822, 551 cm^{-1}. HRMS (ESI) m/z: $[M + Na]^+$ Calcd for $C_{11}H_{14}O_3$ 231.09917; Found 231.09896.

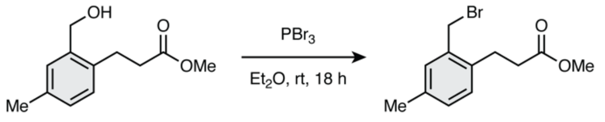

7 **8, 65%**

*Methyl 3-[2-(bromomethyl)-4-methylphenyl]propanoate (**8**).* A mixture of methyl 3-[2-(hydroxy-methyl)-4-methylphenyl]propanoate (**7**; 1.04 g, 4.99 mmol, 1.0 equiv.) and phosphorus tribromide (0.50 mL, 5.3 mmol, 1.1 equiv.) in Et_2O (20 mL) was stirred at room temperature for 18 h before it was carefully poured into water. The resulting mixture was extracted with CH_2Cl_2 and the combined organic layers were dried over anhydrous $MgSO_4$ and filtered. After evaporation of the solvents, the residue was purified by column chromatography over silica gel using CH_2Cl_2 as an eluent to afford the desired product (879 mg, 65%) as a colorless liquid. ^{1}H NMR (400 MHz, $CDCl_3$, ppm): δ 7.15 (s, 1H), 7.10–7.06 (m, 2H), 4.54 (s, 2H), 3.69 (s, 3H), 3.04 (t, J = 7.9 Hz, 2H), 2.69 (t, J = 7.9 Hz, 2H), 2.31 (s, 3H). ^{13}C NMR (101 MHz, $CDCl_3$, ppm): δ 173.4, 136.8, 136.5, 135.5,

131.5, 130.2, 129.6, 51.9, 35.1, 31.8, 26.9, 21.0. IR: 2950, 2923, 1735, 1436, 1210, 1158, 822, 576 cm^{-1}. HRMS (ESI) *m/z*: [*M* + Na]$^+$ Calcd for $C_{11}H_{13}O_2Br$ 293.01476; Found 293.01461.

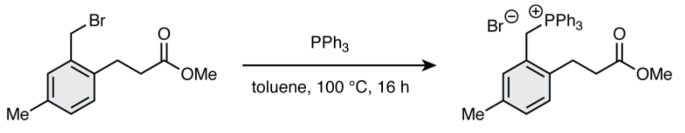

8 **2, >99%**

*[2-(3-Methoxy-3-oxopropyl)-5-methylbenzyl]triphenylphosphonium bromide (**2**).* A mixture of methyl 3-[2-(bromomethyl)-4-methylphenyl]propanoate (**8**; 879 mg, 3.24 mmol, 1.0 equiv.) and triphenyl phosphine (908 mg, 3.46 mmol, 1.1 equiv.) in toluene (30 mL) was stirred at room temperature for 16 h. The precipitate was filtered and washed with hexane. After drying under reduced pressure, the desired product (1.73 g, >99%) was obtained as a colorless powder (mp 194.7–195.4 °C). ^{1}H NMR (400 MHz, $CDCl_3$, ppm): δ 7.83–7.74 (m, 3H), 7.71–7.59 (m, 12H), 7.00 (dt, *J* = 7.9, 2.3 Hz, 1H), 6.93 (d, *J* = 2.3 Hz, 1H), 6.88 (d, *J* = 7.9 Hz, 1H), 5.29 (d, *J* = 14.2 Hz, 2H), 3.61 (s, 2H), 2.34 (t, *J* = 7.5 Hz, 2H), 2.07–2.03 (m, 5H). ^{13}C NMR (101 MHz, $CDCl_3$, ppm): δ 173.4, 137.9 (d, *J* = 6.0 Hz), 137.1 (d, *J* = 3.6 Hz), 135.2 (d, *J* = 3.1 Hz), 134.5 (d, *J* = 9.7 Hz), 132.9 (d, *J* = 5.1 Hz), 130.3 (d, *J* = 12.6 Hz), 130.1 (d, *J* = 4.0 Hz), 129.5 (d, *J* = 2.8 Hz), 125.1 (d, *J* = 8.8 Hz), 117.8 (d, *J* = 85.1 Hz), 52.0, 34.8, 28.0 (d, *J* = 46.7 Hz), 26.6, 20.9. ^{31}P NMR (162 MHz, $CDCl_3$): δ 22.44. IR: 1716, 1434, 1424, 1202, 1107, 833, 749, 738, 720, 712, 689, 517, 505, 493, 473 cm^{-1}. HRMS (ESI) *m/z*: [*M* – Br]$^+$ Calcd for $C_{29}H_{28}O_2PBr$ 453.1978; Found 453.1979.

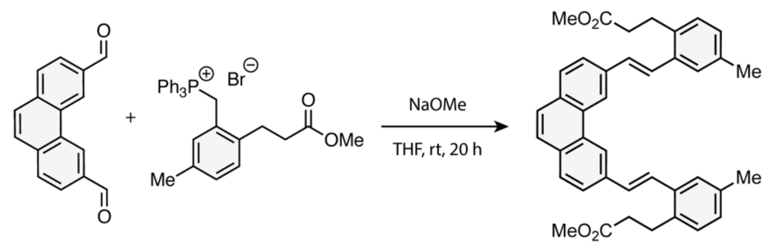

3 **2** **4, 71%**

*Dimethyl 3,3'-{[(1E,1'E)-phenanthrene-3,6-diylbis(ethene-2,1-diyl)]bis(4-methyl-2,1-phenylene)}dipropionate (**4**).* A mixture of [2-(3-methoxy-3-oxopropyl)-5-methylbenzyl]triphenylphosphonium bromide (**2**; 1.50 g, 2.81 mmol, 2.1 equiv.) and anhydrous THF (40 mL) was stirred at room temperature before sodium methoxide (5.4 M in MeOH, 0.50 mL, 3.6 mmol, 2.6 equiv.) was added dropwise and the resulting mixture was stirred for 5 min. Then, phenanthrene-3,6-dicarbaldehyde (**3**; 320 mg, 1.37 mmol, 1.0 equiv.) was added in three portions as a solid and the resulting mixture was stirred for 20 h at room temperature. The reaction mixture was poured into aq. HCl (2 M) and extracted with CH_2Cl_2. The combined organic layers were dried over anhydrous $MgSO_4$ and filtered. The solvent was evaporated and the residue was purified by column chromatography over silica gel using CH_2Cl_2 as an eluent to afford an isomeric mixture of the product (569 mg, 71%) as a pale yellow solid (mp 104.3–105.1 °C). One fraction contained pure (*E*,*E*)-isomer and was used for NMR characterization: ^{1}H NMR (400 MHz, CD_2Cl_2, ppm): δ 8.85 (s, 2H), 7.92 (s, 4H), 7.75 (s, 2H), 7.64 (d, *J* = 16.0 Hz, 2H), 7.61–7.55 (m, 2H), 7.38 (d, *J* = 16.0 Hz, 2H), 7.16 (d, *J* = 7.8 Hz, 2H), 7.11 (dd, *J* = 7.8, 1.8 Hz, 2H), 3.66 (s, 6H), 3.17 (t, *J* = 7.8 Hz, 4H), 2.67 (t, *J* = 7.9 Hz, 4H), 2.43 (s, 6H). ^{13}C NMR (101 MHz, CD_2Cl_2, ppm): δ 173.6, 136.8, 136.4, 136.2, 136.1, 132.4, 131.0, 130.9, 129.9, 129.4, 129.1, 127.0, 126.9, 126.8, 124.8, 122.1, 51.9, 35.9, 28.6, 21.3. IR: 2917, 2849, 1735, 1435, 1197, 1163, 1033, 964, 882, 838, 549 cm^{-1}. HRMS (ESI) *m/z*: [*M* + H]$^+$ Calcd for $C_{40}H_{38}O_4$ 583.28429; Found 583.28415.

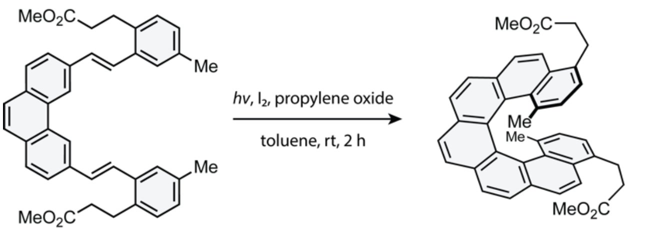

9, 75%

4

*(±)-Dimethyl 3,3'-(1,18-dimethylbenzo[1,2-c:4,3-c']diphenanthrene-4,15-diyl)dipropionate (**9**).* The photocyclodehydrogenation reaction was performed in an immersion-well photoreactor system with a quartz cooling tube and quartz immersion tubes containing 150 W medium-pressure mercury lamp. An argon-saturated solution of dimethyl 3,3'-{[(1*E*,1'*E*)-phenanthrene-3,6-diylbis(ethene-2,1-diyl)]bis(4-methyl-2,1-phenylene)}dipropionate (**4**; 176 mg, 0.301 mmol, 1.0 equiv.), iodine (170 mg, 0.670 mmol, 2.2 equiv.), and propylene oxide (10.0 mL, 143 mmol, 473 equiv.) in toluene (650 mL) was irradiated for 2 h. The resulting solution was washed with saturated aq. $Na_2S_2O_3$ and the organic layer was separated. The

solvent was evaporated and the residue was purified by column chromatography over silica gel using CH_2Cl_2 as an eluent to afford the desired product (128 mg, 75%) as a yellow solid (mp 188.5–192.4 °C). ^{1}H NMR (400 MHz, CD_2Cl_2, ppm): δ 8.02–7.99 (m, 4H), 7.87 (d, J = 8.1 Hz, 2H), 7.59 (d, J = 8.7 Hz, 2H), 7.47 (d, J = 8.7 Hz, 2H), 6.77 (d, J = 7.3 Hz, 2H), 6.30 (dd, J = 7.3, 0.9 Hz, 2H), 3.69 (s, 6H), 3.23 (ddd, J = 14.3, 10.3, 5.6 Hz, 2H), 2.97 (ddd, J = 14.4, 10.3, 6.4 Hz, 2H), 2.67 (ddd, J = 15.9, 10.3, 5.6 Hz, 2H), 2.55 (ddd, J = 15.8, 10.4, 6.4 Hz, 2H), 0.63 (s, 6H). ^{13}C NMR (101 MHz, CD_2Cl_2, ppm): δ 173.7, 133.0, 131.8, 131.4, 131.1, 130.3, 130.2, 128.4, 128.1, 127.0, 126.6, 126.3, 125.8, 124.9, 124.6, 123.4, 51.9, 35.5, 28.4, 21.4. IR: 1735, 1433, 1287, 1166, 845, 833, 823, 811, 611, 517 cm^{-1}. HRMS (ESI) m/z: $[M + H]^+$ Calcd for $C_{40}H_{34}O_4$ 579.2530; Found 579.2528. The structure was additionally validated by SC-XRD (see X-Ray Crystallography section).

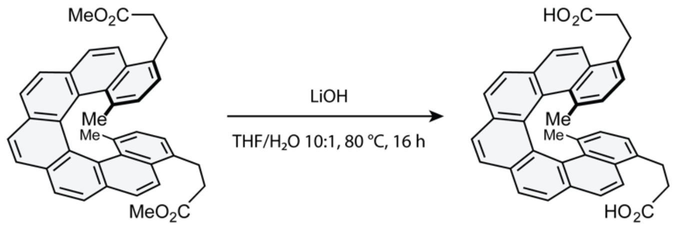

9 **10**, >99%

*3,3'-(1,18-Dimethylbenzo[1,2-c:4,3-c']diphenanthrene-4,15-diyl)dipropionic acid (**10**).* A mixture of dimethyl 3,3'-(1,18-dimethylbenzo[1,2-*c*:4,3-*c*']diphenanthrene-4,15-diyl)dipropionate (**9**; 121 mg, 0.209 mmol, 1.0 equiv.) and lithium hydroxide monohydrate (50.1 mg, 2.09 mmol, 10 equiv.) in THF/H_2O (11 mL, 10:1) was heated at 80 °C for 16 h. The reaction mixture was cooled to room temperature and concentrated under reduced pressure. The residue was acidified with aq. HCl (2 M), filtered, and washed with water and CH_2Cl_2. Drying under reduced pressure afforded the desired product (115 mg, >99%) as a yellow solid (mp 301.2–302.7 °C) that was used in the next step without further purification. Due to the poor solubility of the compound, only a ^{1}H NMR spectrum could be acquired: ^{1}H NMR (400 MHz, CD_3OD, ppm): δ 8.02 (s, 1H), 8.01 (d, J = 8.0 Hz, 2H), 7.88 (d, J = 8.1 Hz, 2H), 7.60 (d, J = 8.7 Hz, 2H), 7.51 (d, J = 8.8 Hz, 2H), 6.79 (d, J = 7.3 Hz, 2H), 6.27 (d, J = 7.4 Hz, 2H), 3.25–3.19 (m, J = 10.2, 9.7, 5.0 Hz, 2H, partial overlap with the solvent residual peak), 2.98 (ddd, J = 15.2, 10.0, 6.4 Hz, 2H), 2.65 (ddd, J = 15.7, 10.1, 5.6 Hz, 2H), 2.52 (ddd, J = 16.2, 10.1, 6.4 Hz, 2H), 0.60 (s, 6H). IR: 2925, 1705, 1412, 1295, 1209, 847, 825, 813, 612, 521 cm^{-1}. HRMS (ESI) m/z: $[M + Na]^+$ Calcd for $C_{38}H_{30}O_4$ 573.2036; Found 573.2042.

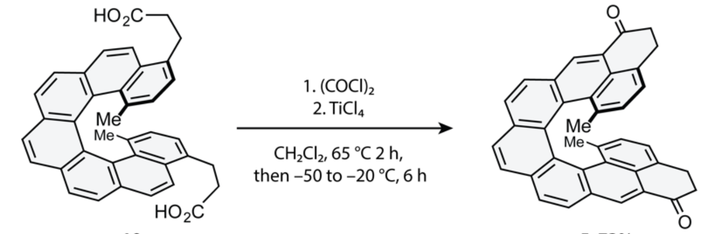

10 **5**, 73%

*(±)-1,20-Dimethyl-4,5,16,17-tetrahydrobenzo[no]benzo[8,9]-anthra[1,2-a]tetraphene-6,15-dione (**5**).* A solution of (±)-3,3'-(1,18-dimethylbenzo[1,2-*c*:4,3-*c*']diphenanthrene-4,15-diyl)dipropionic acid (**10**; 115 mg, 0.209 mmol, 1.0 equiv.) in oxalyl chloride (25 mL) was heated at 65 °C for 2 h before the excess of oxalyl chloride was removed under reduced pressure. The crude acyl chloride intermediate was dissolved in CH_2Cl_2 (50 mL) and the solution was cooled to –45 °C. $TiCl_4$ (0.25 mL, 2.3 mmol, 11 equiv.) was added and the reaction mixture was stirred in the temperature range between –45 and –20 °C over 6 h. Then, it was poured into aq. HCl (2 M), extracted with CH_2Cl_2, dried over $MgSO_4$, and filtered. After evaporation of the solvents, the residue was purified by filtration through a short plug of silica gel using CH_2Cl_2 as an eluent to give the desired product (74.8 mg, 73%) as a yellow solid (mp >321 °C decomposition). ^{1}H NMR (400 MHz, CD_2Cl_2, ppm): δ 8.31 (s, 2H), 8.11 (s, 2H), 8.09 (d, J = 8.1 Hz, 2H), 8.05 (d, J = 8.1 Hz, 2H), 6.89 (d, J = 7.3 Hz, 2H), 6.40 (dd, J = 7.2, 0.8 Hz, 2H), 3.27–3.07 (m, 4H), 2.96 (ddd, J = 15.3, 8.2, 6.3 Hz, 2H), 2.77 (ddd, J = 15.3, 7.4, 6.4 Hz, 2H), 0.64 (s, 6H). ^{13}C NMR (101 MHz, CD_2Cl_2, ppm): δ 198.5, 132.4, 131.4, 131.0, 130.4, 129.9, 129.5, 129.2, 129.0, 128.9, 128.1, 127.9 (2×), 127.7, 125.4, 124.2, 38.9, 28.8, 21.3. IR: 1678, 1592, 1244, 1161, 1140, 897, 838, 816, 739, 681, 609, 602, 581, 536, 523, 496 cm^{-1}. HRMS (ESI) m/z: $[M + H]^+$ Calcd for $C_{38}H_{26}O_2$ 515.2006; Found 515.2010. The structure was additionally validated by SC-XRD (see X-Ray Crystallography section).

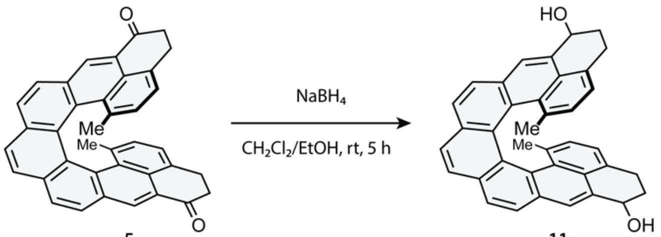

5 **11**

*(±)-1,20-Dimethyl-4,5,6,15,16,17-hexahydrobenzo[no]benzo[8,9]anthra[1,2-a]tetraphene-6,15-diol (**11**).* A solution of (±)-1,20-dimethyl-4,5,16,17-tetrahydrobenzo[*no*]benzo[8,9]anthra[1,2-*a*]tetraphene-6,15-dione (**5**; 40 mg, 0.077 mmol) and sodium borohydride (29 mg, 0.78 mmol) in CH_2Cl_2/EtOH (12 mL, 2:1) was stirred at room temperature for 5 h before the reaction was quenched by the addition of aq. HCl (2 M). The organic layer was separated and the aqueous layer was extracted with CH_2Cl_2. The combined organic layers were washed with saturated aq. $NaHCO_3$, water, and brine, dried over anhydrous Na_2SO_4, and filtered. Evaporation of the solvents afforded the desired product (37 mg, 92%) as a brown solid and as a mixture of three possible diastereomers, which was used in the next step without purification. IR: 2918, 2849, 1176, 1140, 1082, 1029, 830, 612, 500 cm^{-1}. HRMS (ESI) *m/z*: $[M + Na]^+$ Calcd for $C_{38}H_{30}O_2$ 541.2138; Found 541.2137.

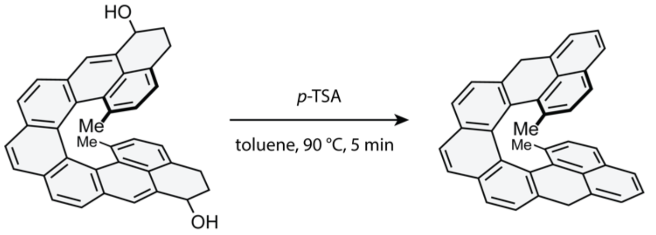

11 2*H*-**DMNC, 96%**

*(±)-1,20-Dimethyl-7,14-dihydrobenzo[no]benzo[8,9]anthra[1,2-a]tetraphene (2H-**DMNC**).* *p*-Toluene-sulfonic acid monohydrate (9.30 mg, 48.9 µmol, 0.3 equiv.) was added to a hot (90 °C) solution of (±)-1,20-dimethyl-4,5,6,15,16,17-hexahydrobenzo[*no*]benzo[8,9]anthra[1,2-*a*]tetraphene-6,15-diol (**11**; 80.0 mg, 163 µmol, 1.0 equiv.) in toluene (15 mL) and the reaction mixture was heated at 90 °C for 5 min before it was cooled in an ice bath and passed through a pad of silica gel using argon-saturated toluene/hexane (1:1) as an eluent. Evaporation of the solvent afforded the desired product (71 mg, 96%) as a yellow solid (mp >173 °C decomposition). ^{1}H NMR (500 MHz, CD_2Cl_2, ppm): δ 7.71 (d, *J* = 7.7 Hz, 2H), 7.62 (s, 2H), 7.43 (dt, *J* = 7.7, 0.9 Hz, 2H), 7.24 (dd, *J* = 8.1, 6.7 Hz, 2H), 7.13 (dt, *J* = 8.1, 1.3 Hz, 2H), 7.07 (ddt, *J* = 6.7, 2.1, 1.1 Hz, 2H), 6.45 (d, *J* = 8.2 Hz, 2H), 6.33 (d, *J* = 8.2 Hz, 2H), 4.29 (d, *J* = 21.4 Hz, 2H), 4.14 (d, *J* = 21.4 Hz, 2H), 0.87 (s, 6H). ^{13}C NMR (126 MHz, CD_2Cl_2, ppm): δ 136.8, 132.7, 132.4, 132.1, 131.1 (2×), 130.6, 130.2, 129.5, 129.1, 126.9, 126.2, 126.1, 125.6, 124.7, 124.6, 122.2, 35.9, 20.6. IR: 2918, 2850, 1029, 832, 811, 778, 739, 557, 489 cm^{-1}. HRMS (APCI) *m/z*: $[M + H]^+$ Calcd for $C_{38}H_{24}$ 483.21073; Found 483.20980.

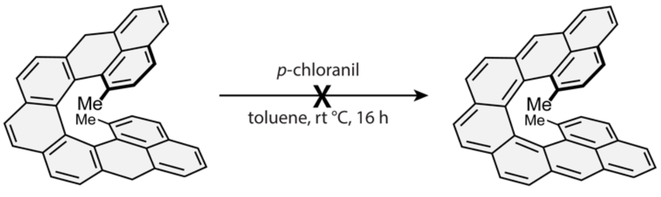

2*H*-**DMNC** **DMNC**

*(±)-1,20-Dimethyl-7,14-dihydrobenzo[no]benzo[8,9]anthra[1,2-a]tetraphene (dimethylnonacethrene, **DMNC**).* To a solution of (±)-1,20-dimethyl-7,14-dihydrobenzo[*no*]benzo-[8,9]anthra[1,2-*a*]tetraphene (2*H*-**DMNC**; 7.00 mg, 14.5 µmol, 1.0 equiv.) in argon-saturated toluene (3 mL), *p*-chloranil (5.35 mg, 21.8 µmol, 1.5 equiv.) was added before the mixture was stirred for 16 h. The resulting dark precipitate was filtered, washed with CH_2Cl_2, and dried *in vacuo*. Due to the limited solubility of the resulting powder, the structure and identity of this compound could not be determined.

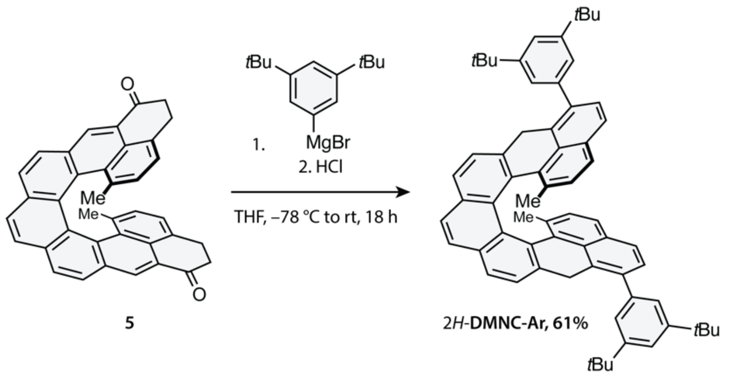

*(±)-6,15-Bis(3,5-di-tert-butylphenyl)-1,20-dimethyl-7,14-dihydrobenzo[no]benzo[8,9]anthra[1,2-a]tetra-phene (2H-**DMNC-Ar**).* To a cooled mixture of 1,20-dimethyl-4,5,16,17-tetrahydrobenzo[*no*]benzo[8,9]-anthra[1,2-*a*]tetraphene-6,15-dione (**5**; 19.5 mg, 0.0379 mmol, 1.0 equiv.) and cerium(III) chloride (17 mg, 0.069 mmol, 1.8 equiv.) in a LiCl solution (0.5 M in THF, 5 mL) at –78 °C, a solution of 3,5-di-*tert*-butylphenyl magnesium bromide (0.2 M in 0.5 M LiCl in THF, 1.0 mL, 0.20 mmol, 5.5 equiv.) was added dropwise before the mixture was slowly allowed to warm to room temperature over 16 h. Then, the mixture was poured into aq. HCl (2 M), extracted with CH_2Cl_2, dried over $MgSO_4$, and filtered. After evaporation of the solvents, the residue was purified by column chromatography over silica gel using CH_3Cl as an eluent to afford the desired product (19.8 mg, 61%) as a yellow solid (mp 84.2–86.1 °C). ^{1}H NMR (500 MHz, C_6D_6, ppm): δ 7.70 (d, J = 2.0 Hz, 2H), 7.61 (s, 4H), 7.45 (d, J = 8.2 Hz, 2H), 7.41 (d, J = 1.8 Hz, 2H), 7.38 (d, J = 7.7 Hz, 2H), 7.25 (d, J = 8.3 Hz, 2H), 6.91 (d, J = 7.7 Hz, 2H), 6.86 (d, J = 8.3 Hz, 2H), 6.75 (d, J = 8.3 Hz, 2H), 4.48 (d, J = 21.1 Hz, 2H), 3.78 (d, J = 21.1 Hz, 2H), 1.48 (s, 36H), 1.12 (s, 6H). ^{13}C NMR (126 MHz, C_6D_6, ppm): δ 151.2, 142.5, 137.1, 137.0, 132.6, 132.1, 131.8, 131.7, 131.1, 131.0, 129.5, 129.1, 127.1, 126.9, 126.1 (2×), 126.0, 125.9, 125.2, 124.5, 120.9, 35.2, 34.7, 31.8, 21.0. IR: 2954, 1594, 1363, 1304, 1214, 953, 852, 705 cm^{-1}. HRMS (ESI) m/z: $[M + H]^+$ Calcd for $C_{66}H_{66}$ 859.52373; Found 859.52336. The structure was additionally validated by SC-XRD (see X-Ray Crystallography section).

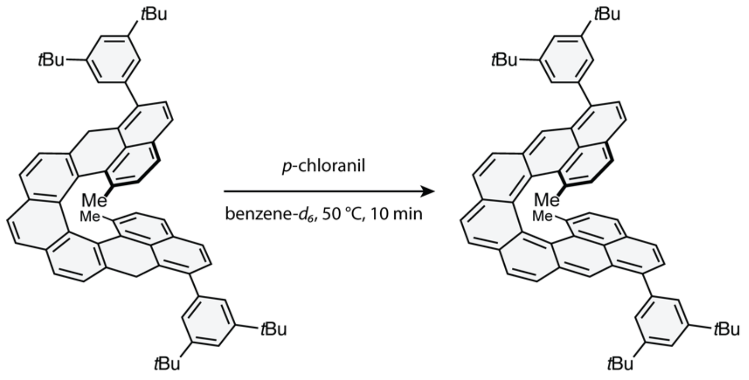

2H-**DMNC-Ar** **DMNC-Ar, 63%**

*(±)-1,20-Dimethylbenzo[no]benzo[8,9]anthra[1,2-a]tetraphene (**DMNC-Ar**).* To a solution of 6,15-bis(3,5-di-*tert*-butylphenyl)-1,20-dimethyl-7,14-dihydrobenzo[*no*]benzo[8,9]anthra[1,2-*a*]tetraphene (2*H*-**DMNC-Ar**; 6.0 mg, 7.0 μmol, 1.0 equiv.) in argon-saturated benzene-d_6 (0.5 mL) in an NMR tube, an argon-saturated solution of *p*-chloranil (0.05 M in benzene-d_6, 210 μL, 10.4 μmol, 1.5 equiv.) was added before the mixture was heated at 50 °C in a water bath for 10 min. The crude mixture was purified by column chromatography over silica gel using toluene as an eluent to obtain the desired product (3.76 mg, 63%) as a deep-purple solid (mp 95.8–97.1 °C). IR: 2953, 2923, 1408, 1307, 1201, 883, 715 cm^{-1}. HRMS (APCI) m/z: $[M + H]^+$ Calcd for $C_{66}H_{64}$ 857.50808; Found 857.50771.

NMR Assignments

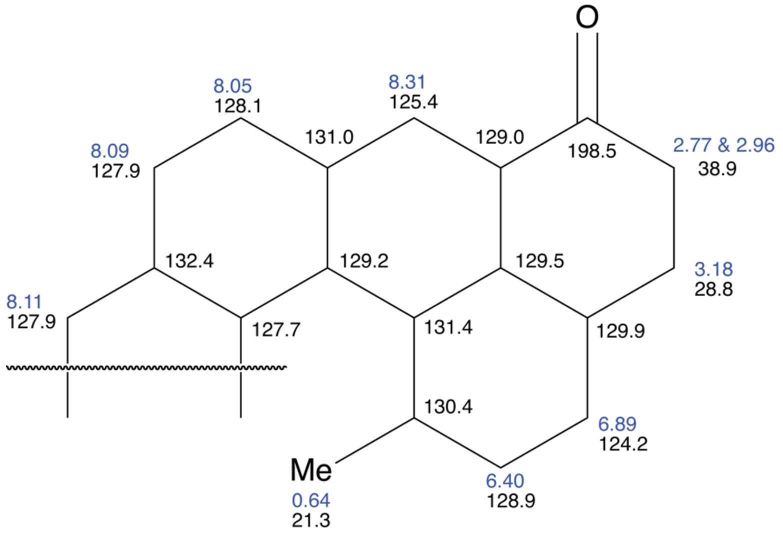

Figure S2: Assignment of ^{1}H (blue) and ^{13}C (black) NMR resonances (in ppm) of **5**. π-Bonds are omitted for clarity.

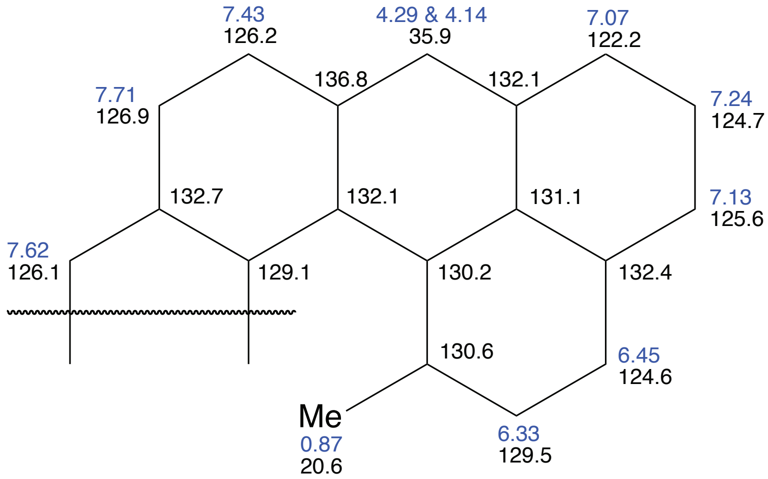

Figure S3: Assignment of ^{1}H (blue) and ^{13}C (black) NMR resonances (in ppm) of 2*H*-**DMNC**. π-Bonds are omitted for clarity.

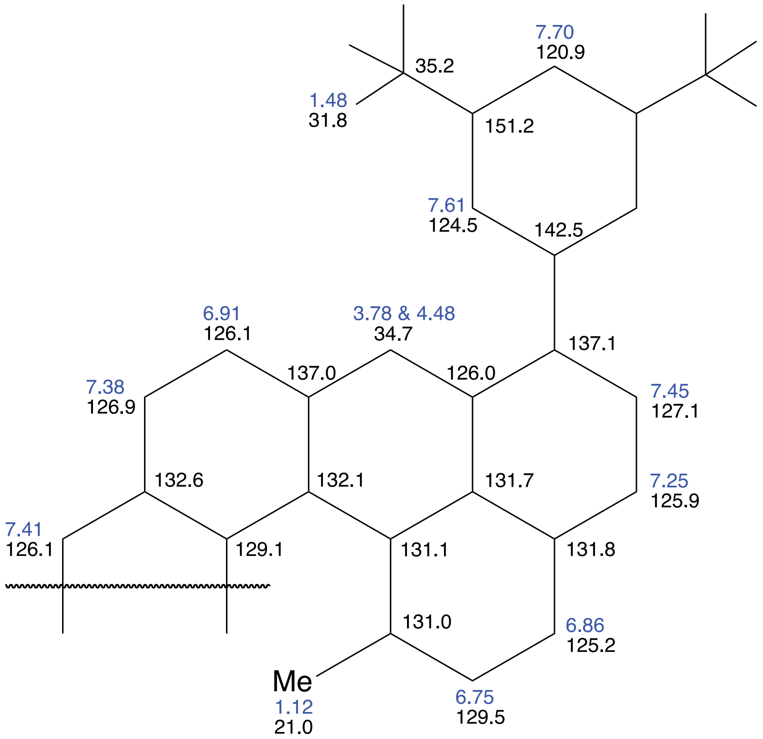

Figure S4: Assignment of ^{1}H (blue) and ^{13}C (black) NMR resonances (in ppm) of 2*H*-**DMNC-Ar**. π-Bonds are omitted for clarity.

Copies of NMR Spectra

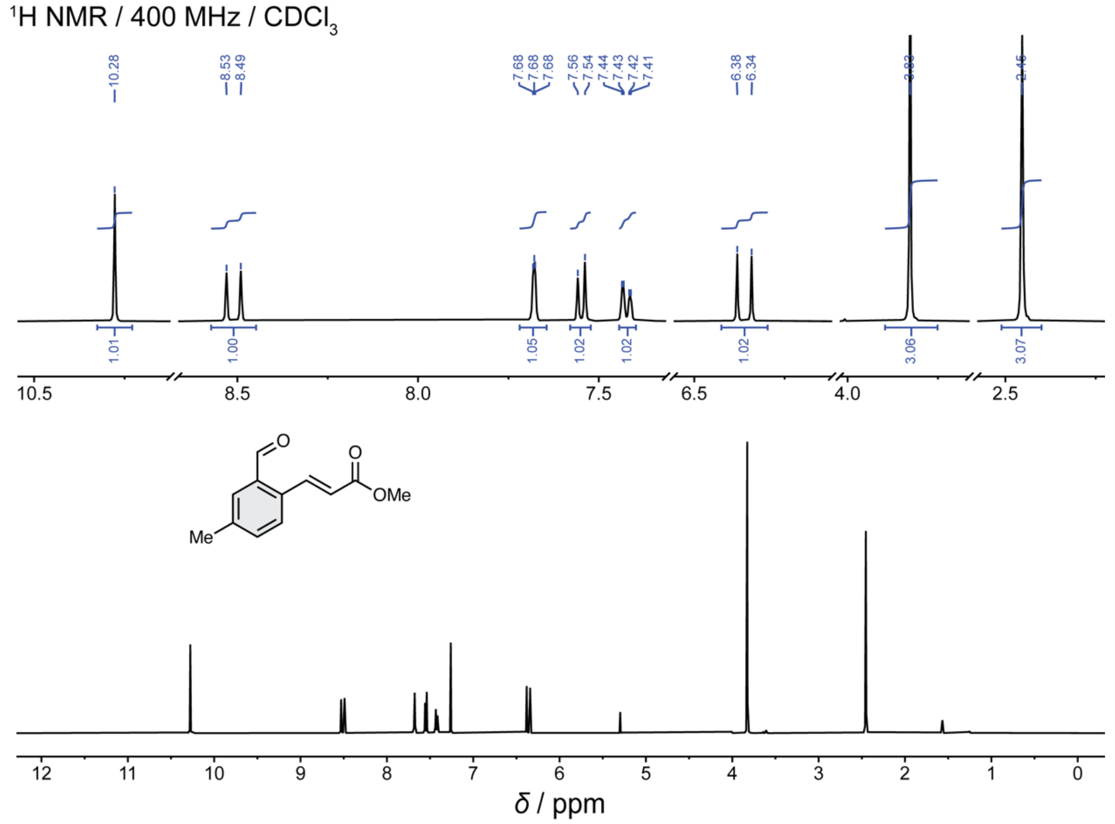

Figure S5: ^{1}H NMR spectrum of **6**.

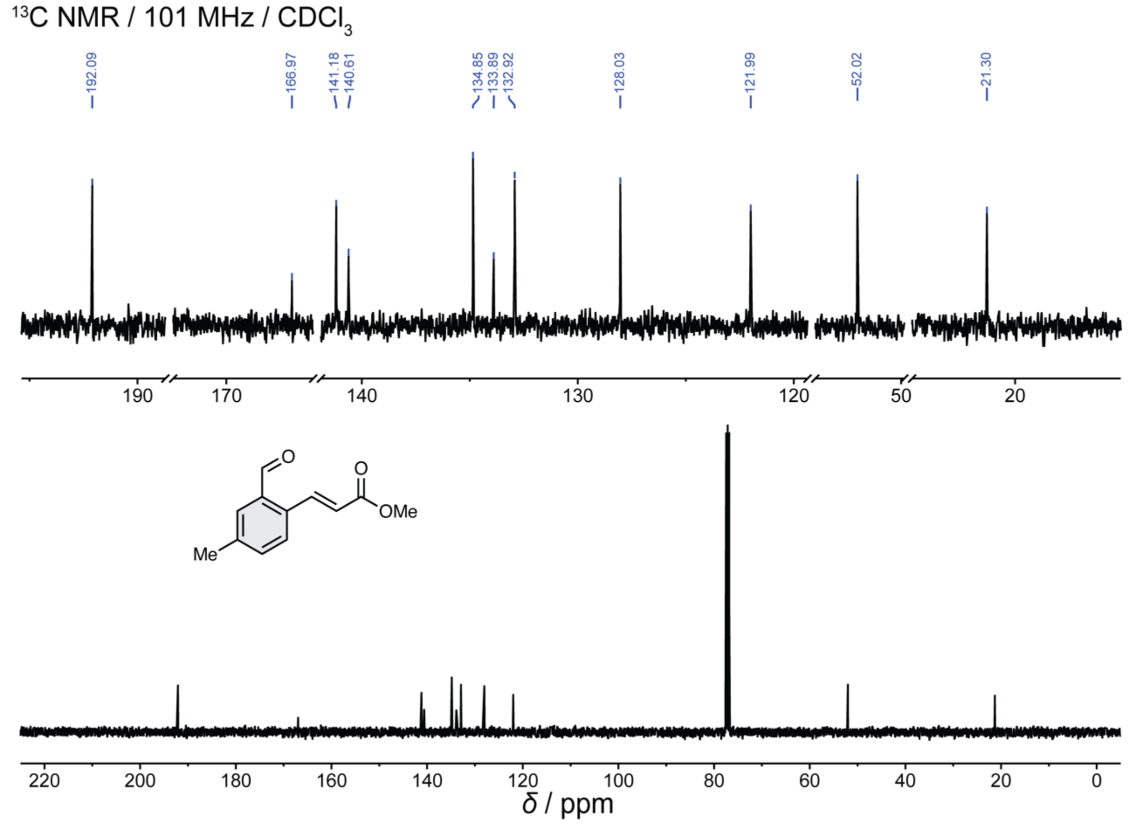

Figure S6: ^{13}C NMR spectrum of **6**.

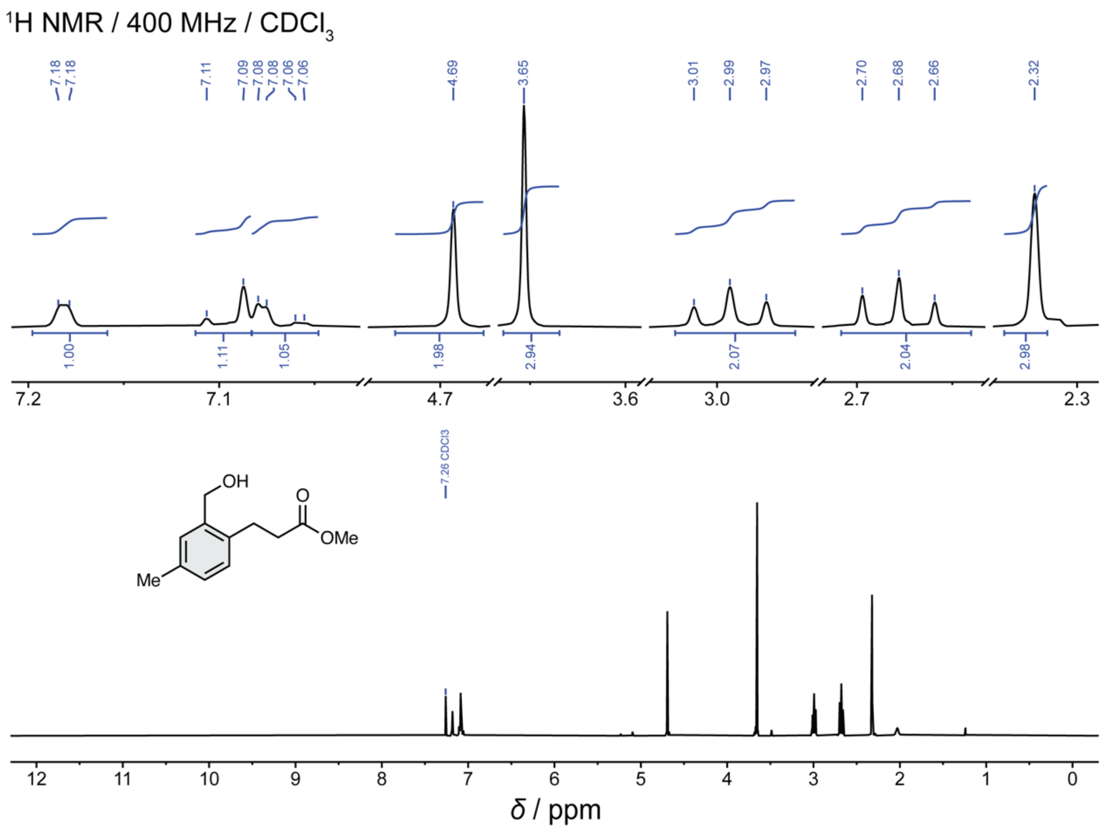

Figure S7: ^{1}H NMR spectrum of **7**.

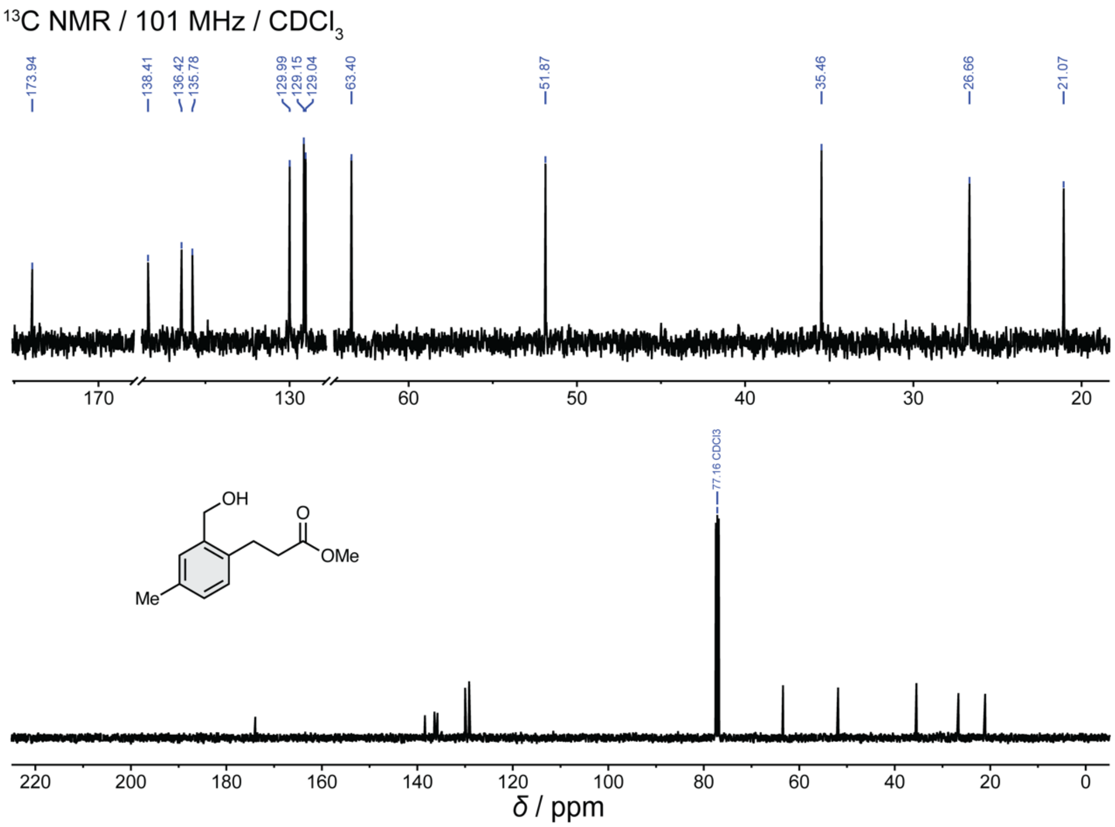

Figure S8: ^{13}C NMR spectrum of **7**.

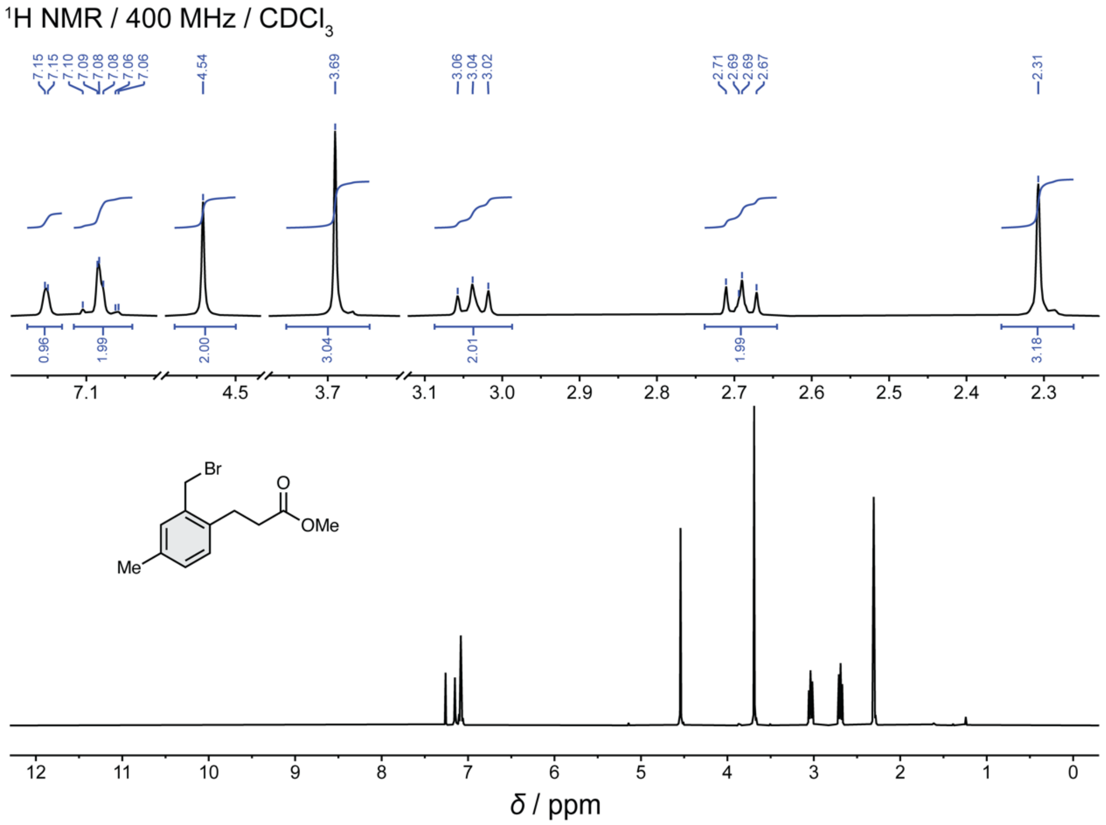

Figure S9: ^{1}H NMR spectrum of **8**.

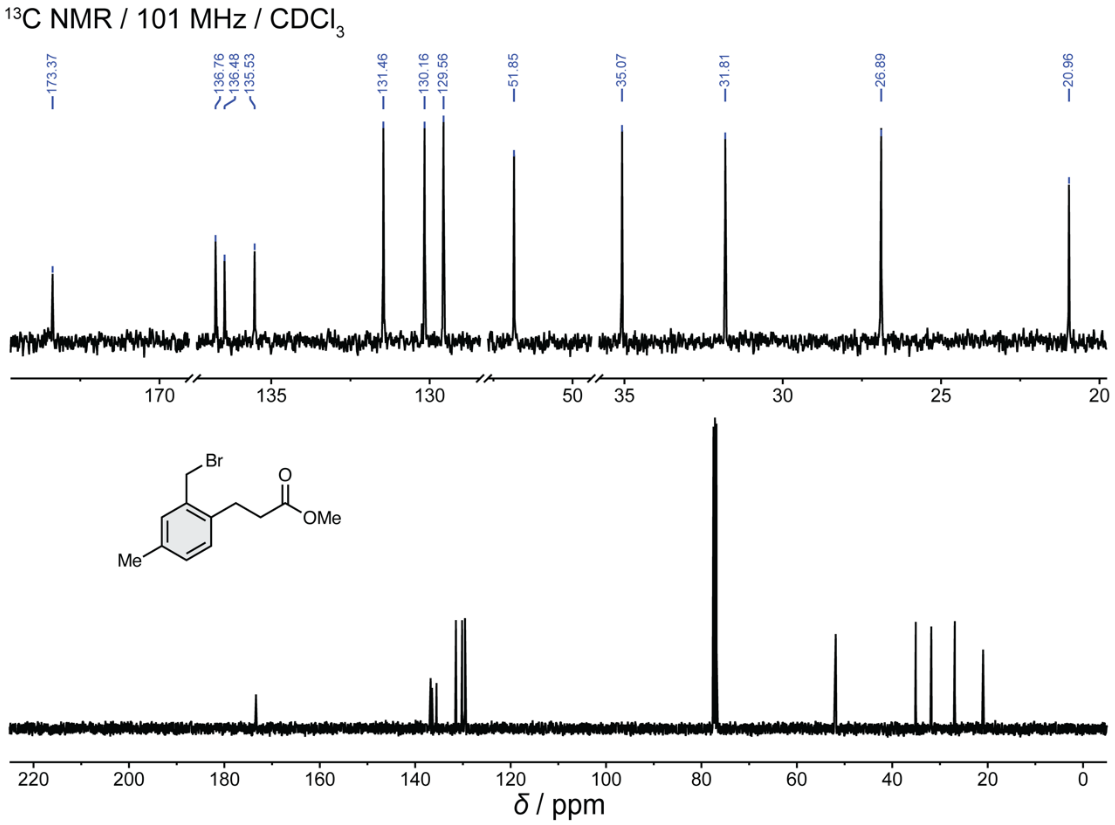

Figure S10: ^{13}C NMR spectrum of **8**.

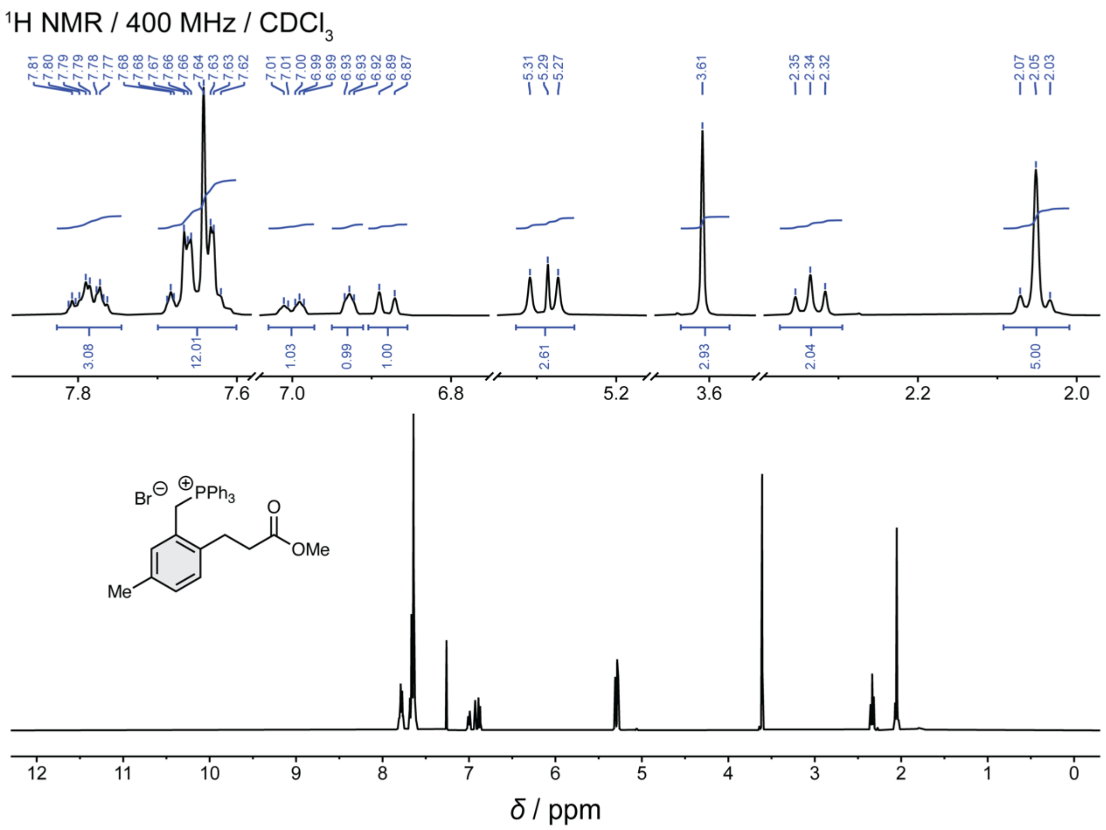

Figure S11: ¹H NMR spectrum of **2**.

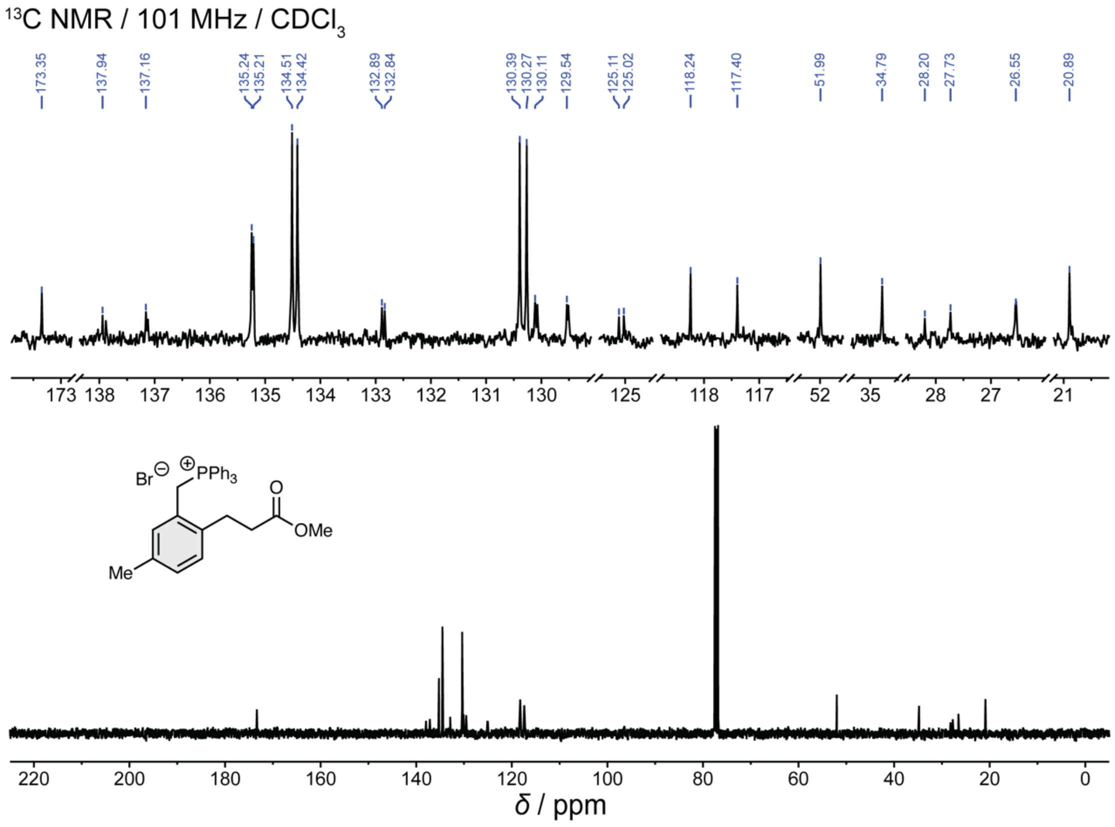

Figure S12: ¹³C NMR spectrum of **2**.

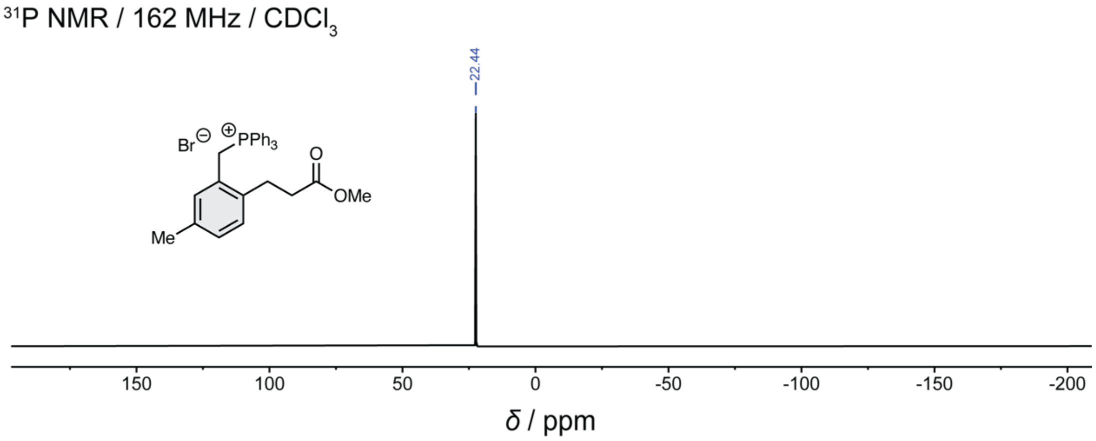

Figure S13: ^{31}P NMR spectrum of **2**.

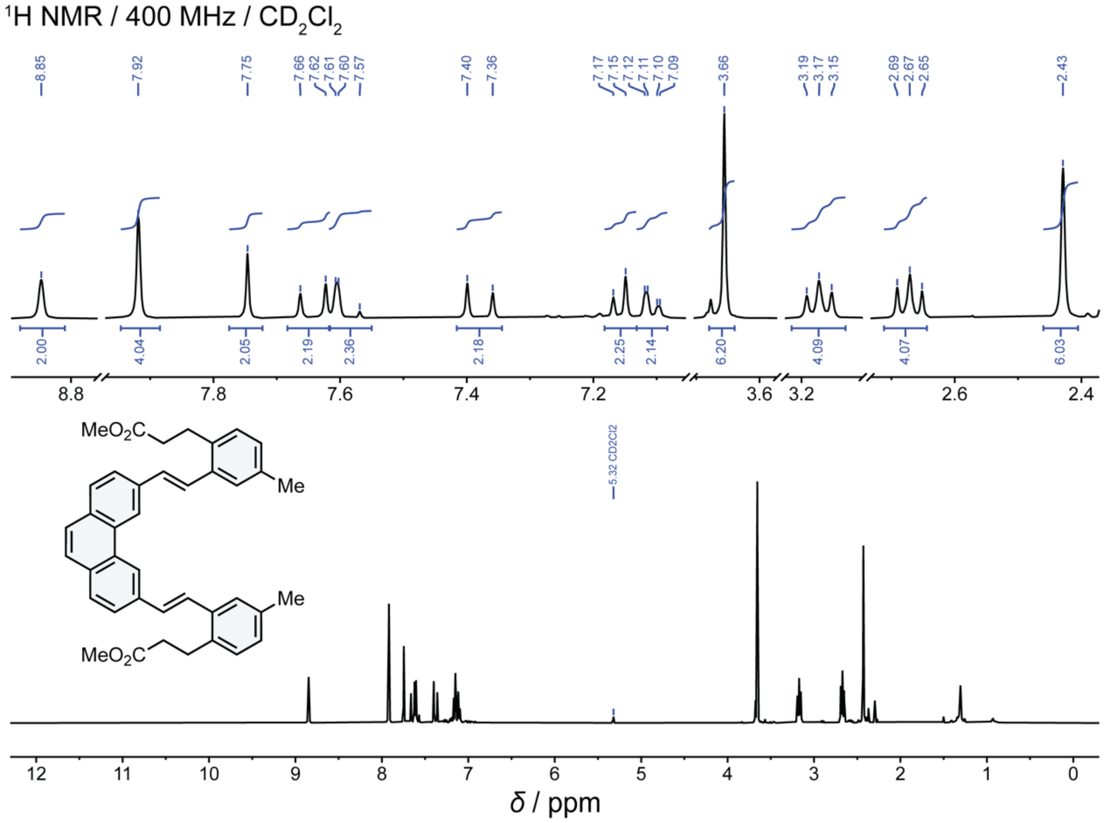

Figure S14: ^{1}H NMR spectrum of **4**.

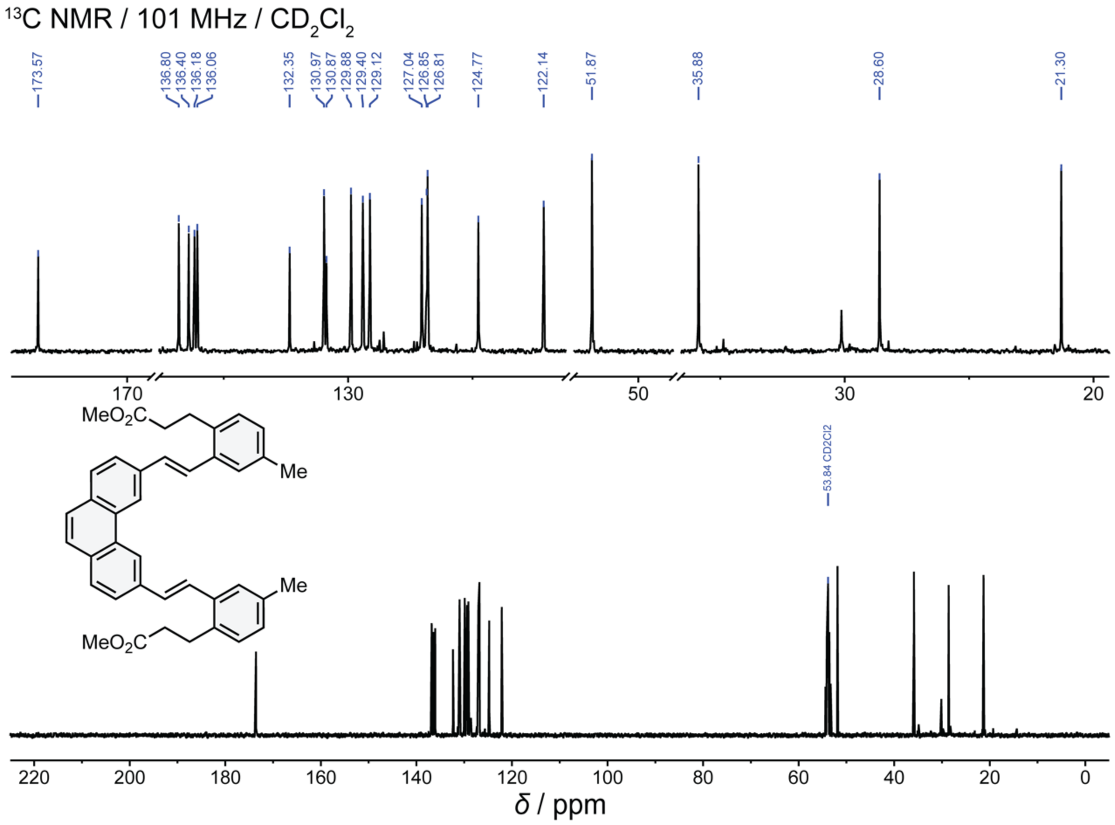

Figure S15: ^{13}C NMR spectrum of **4**.

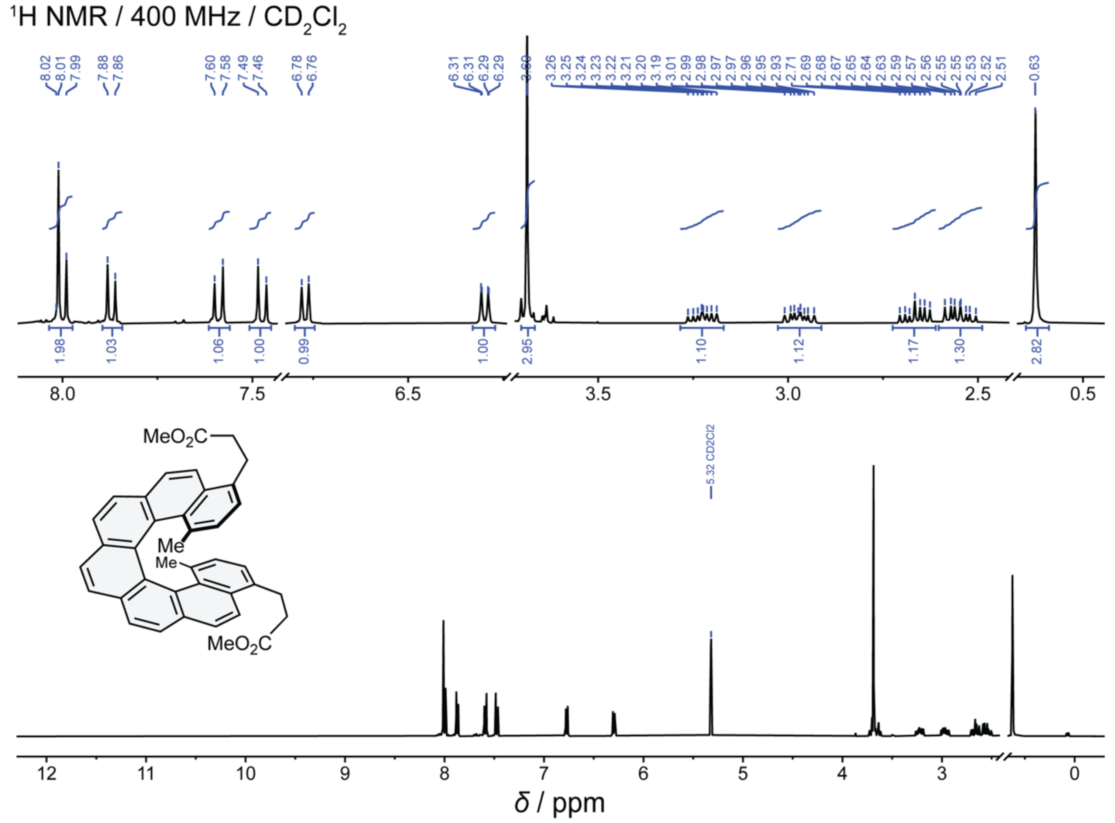

Figure S16: ^{1}H NMR spectrum of **9**.

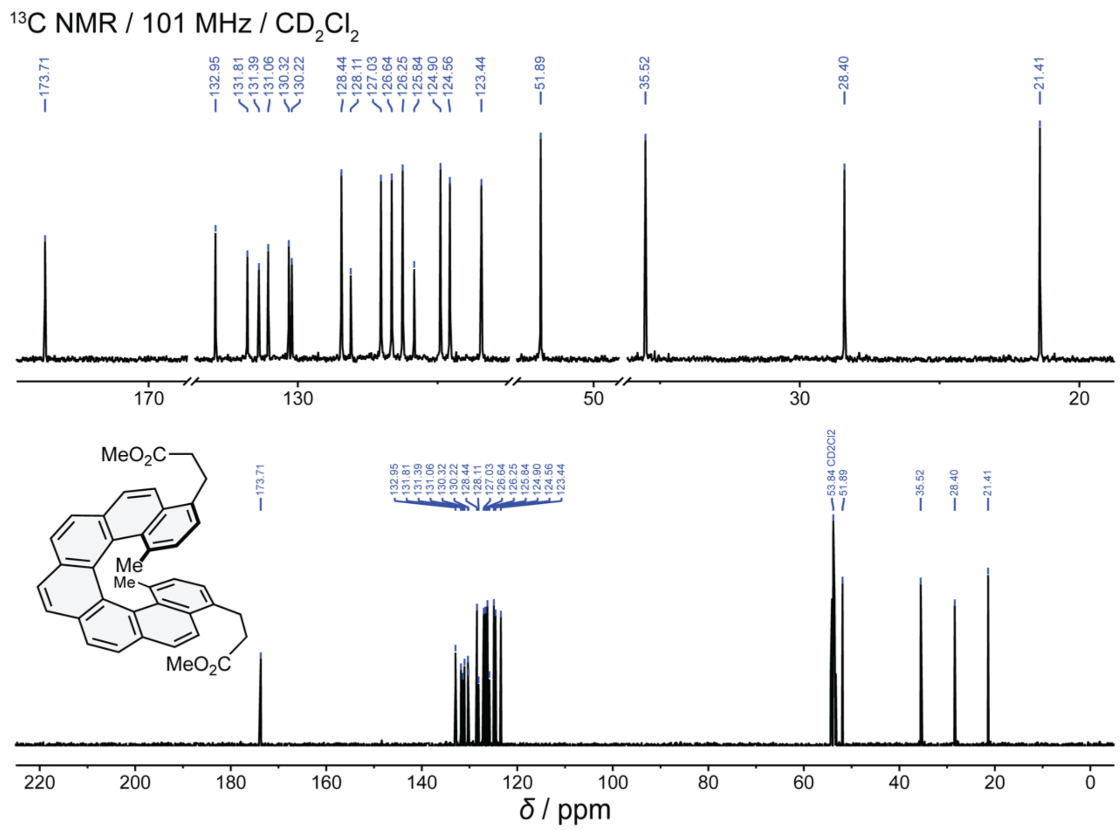

Figure S17: ^{13}C NMR spectrum of **9**.

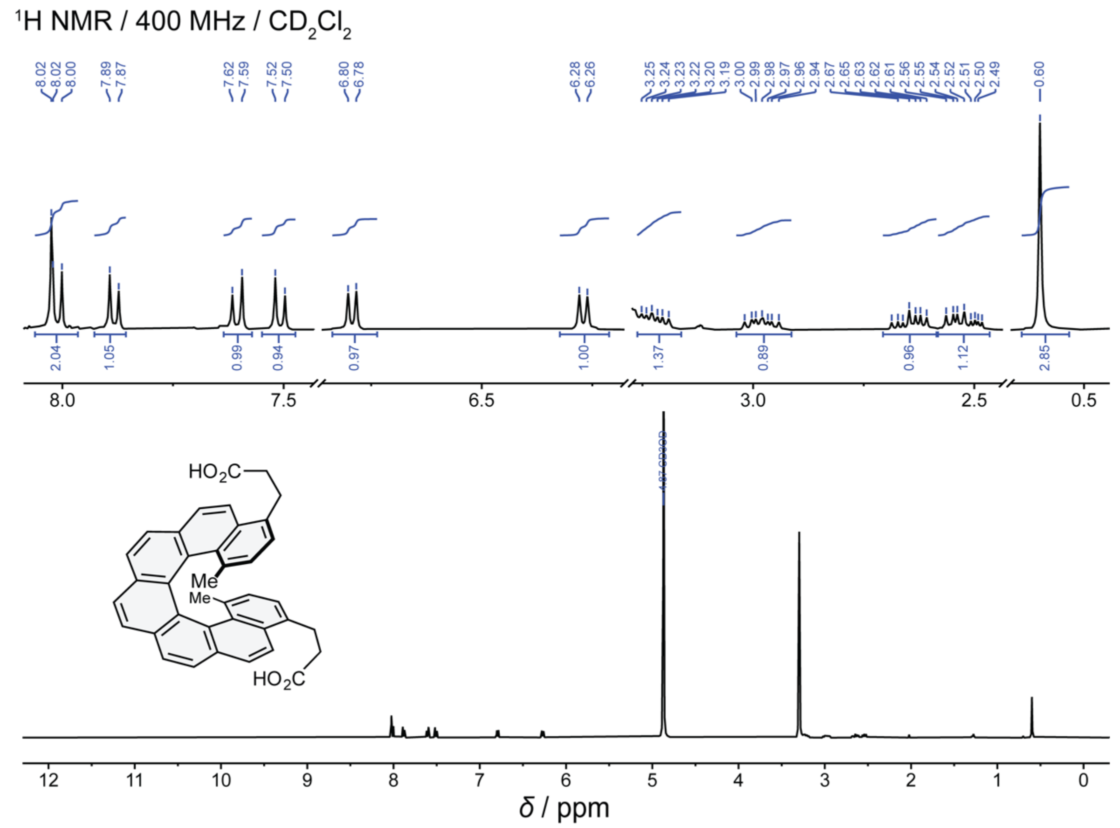

Figure S18: ^{1}H NMR spectrum of **10**.

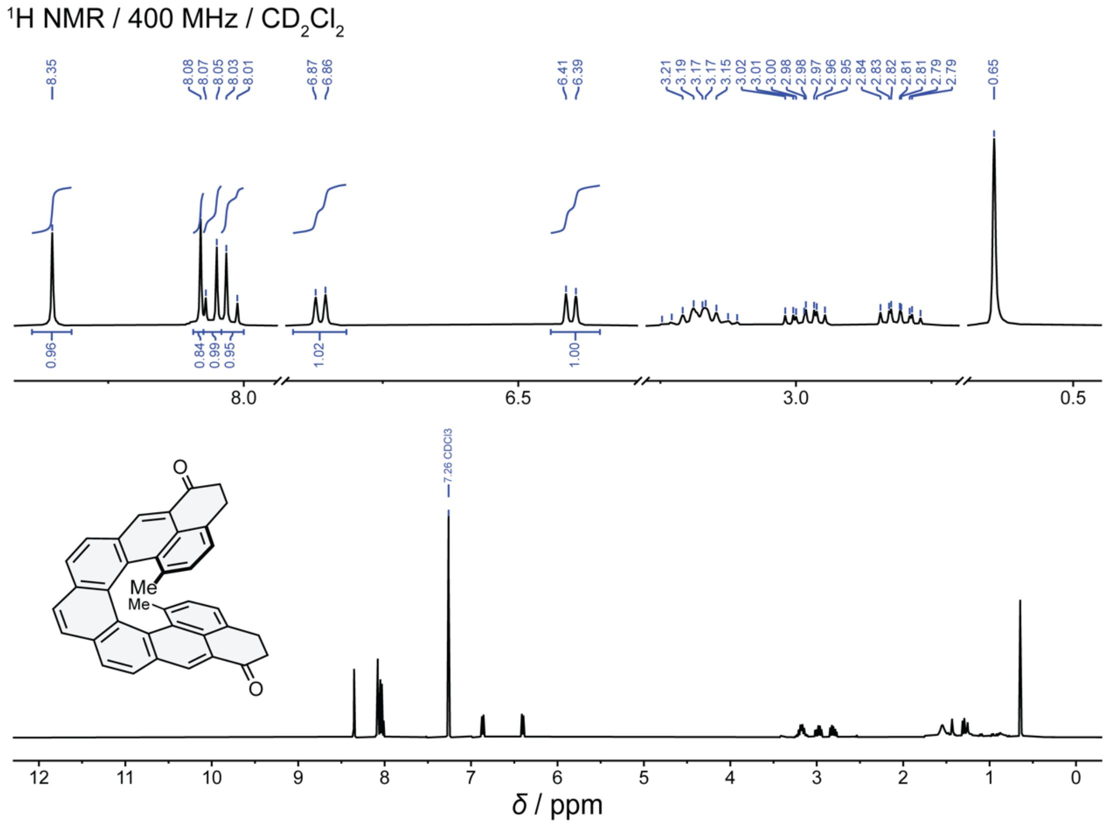

Figure S19: ¹H NMR spectrum of **5**.

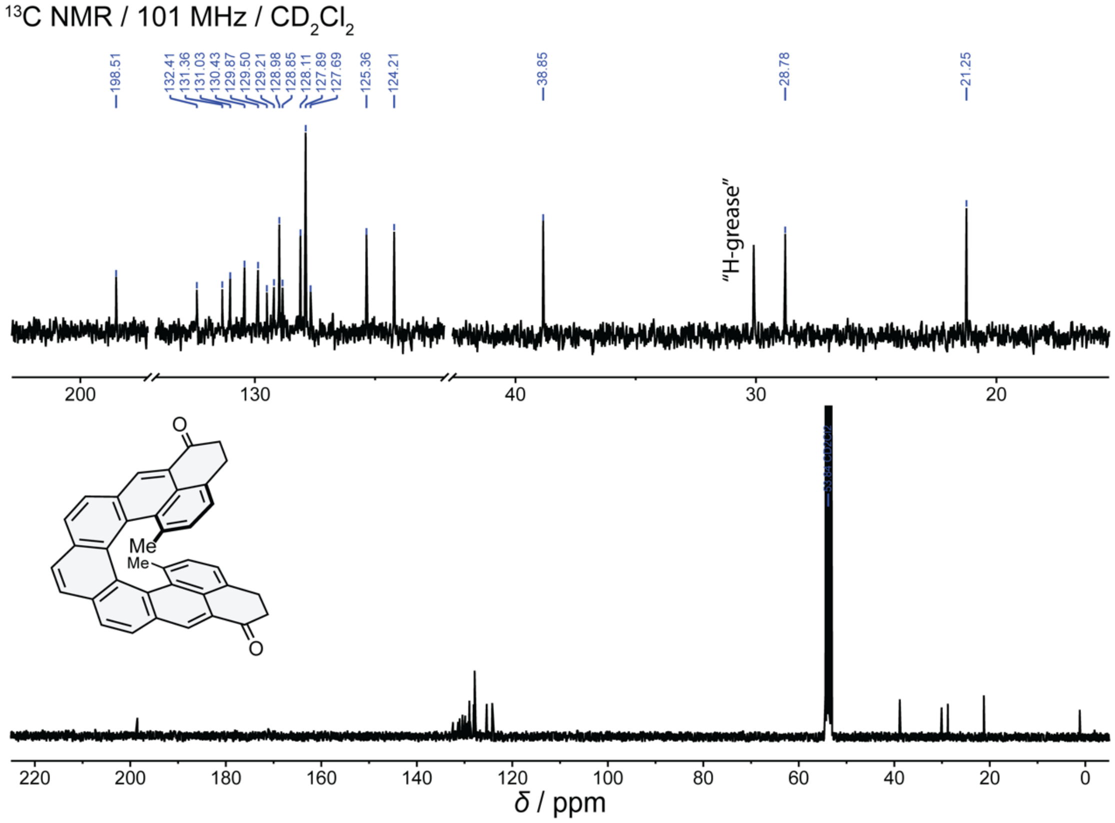

Figure S20: ¹³C NMR spectrum of **5**.

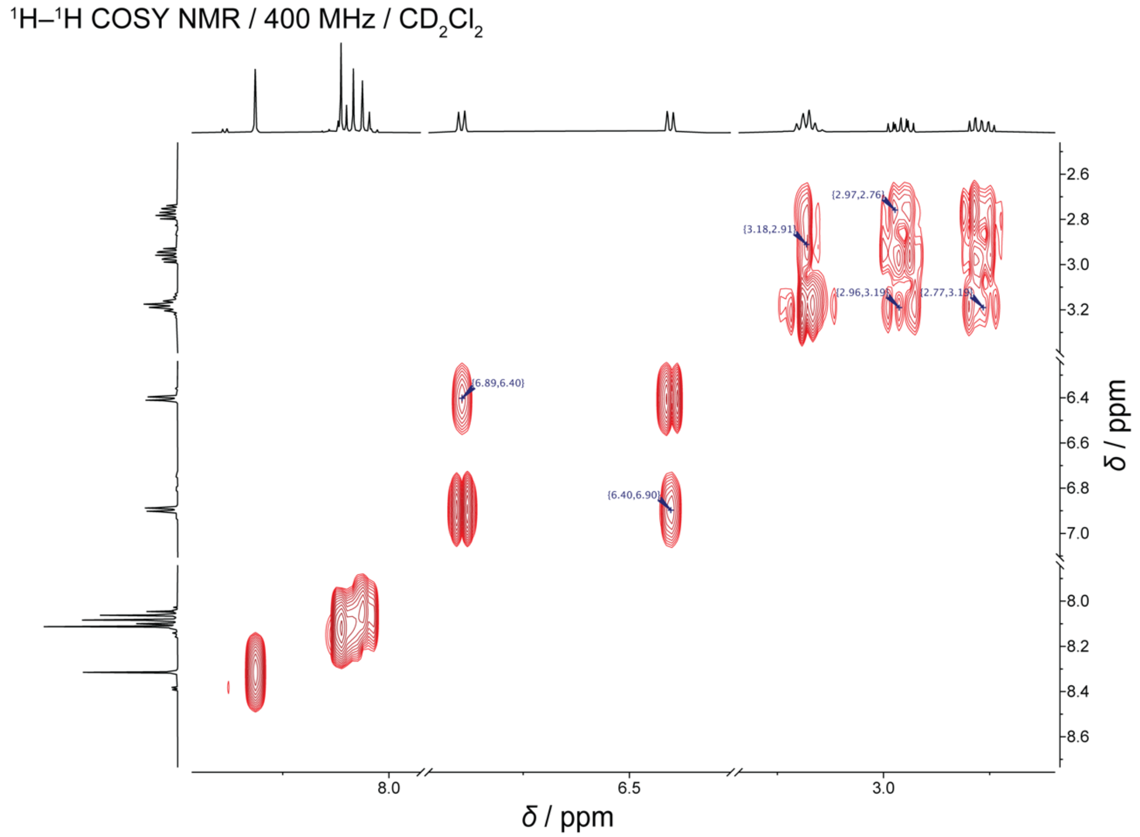

Figure S21: ^1H–^1H COSY spectrum of **5**.

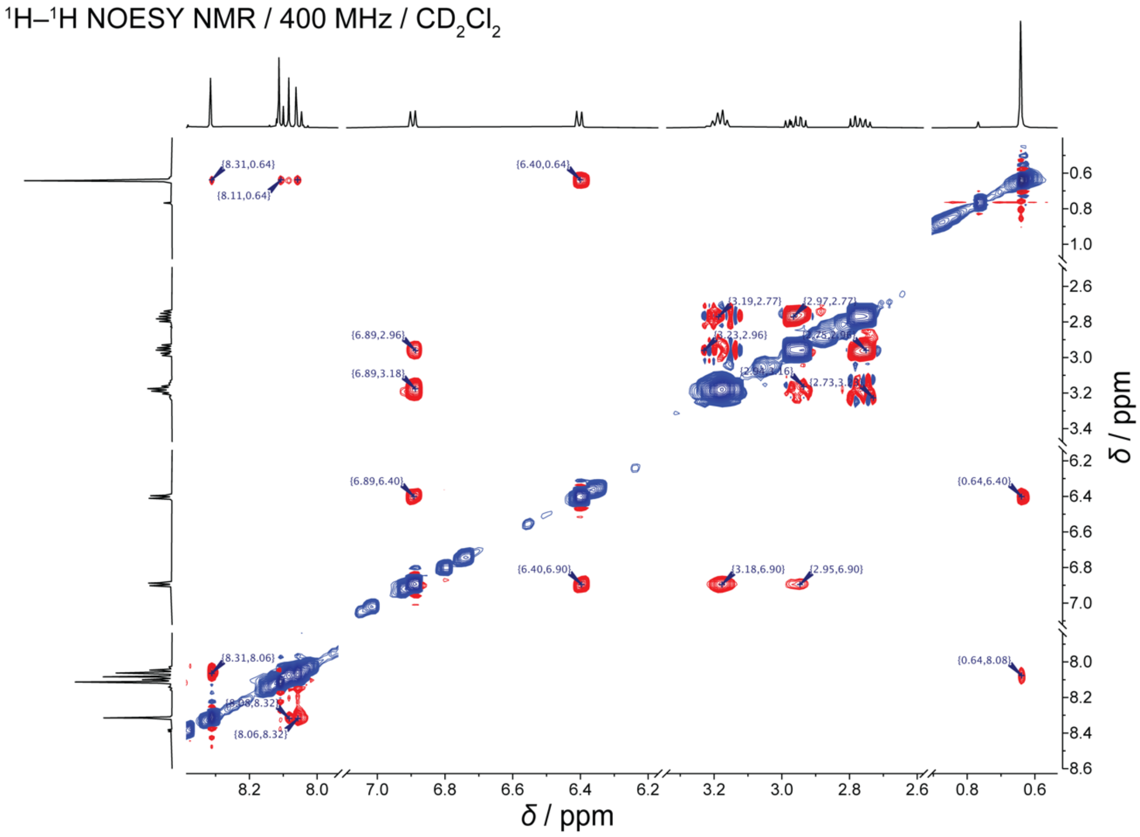

Figure S22: ^1H–^1H NOESY spectrum of **5**.

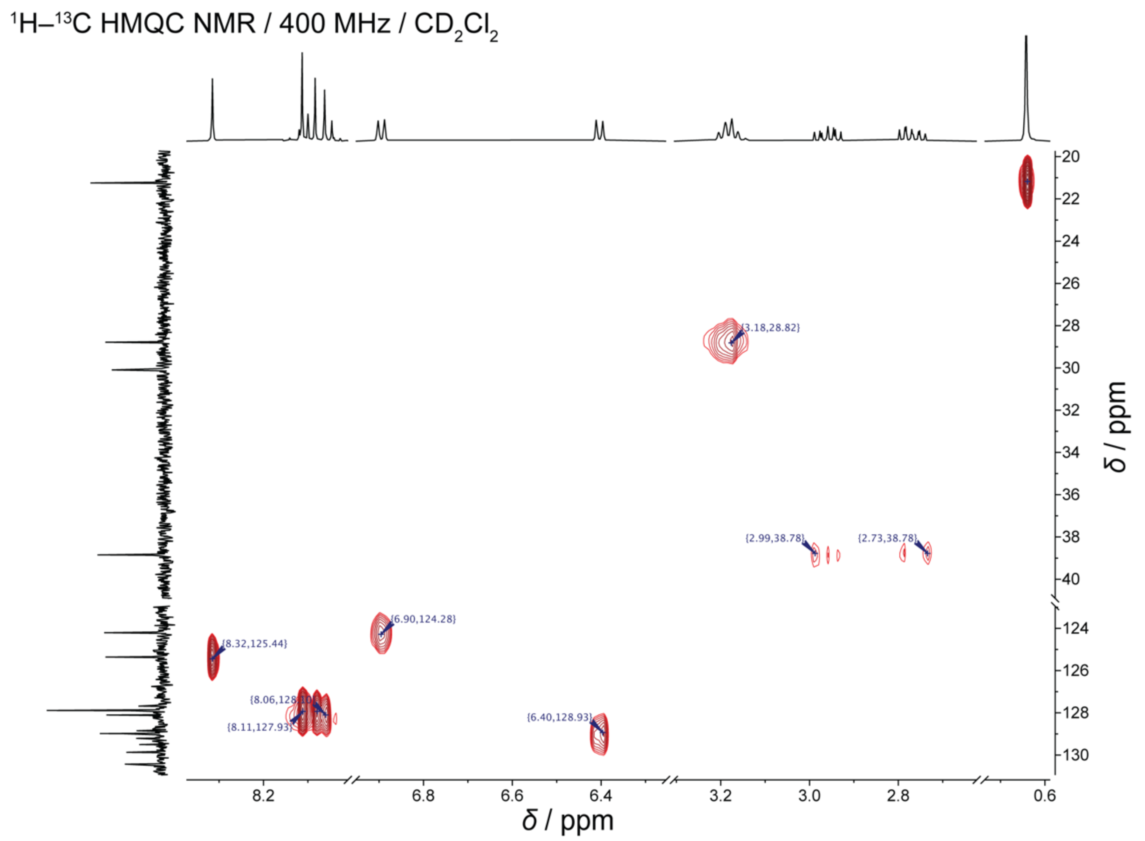

Figure S23: ^1H–^{13}C HMQC spectrum of **5**.

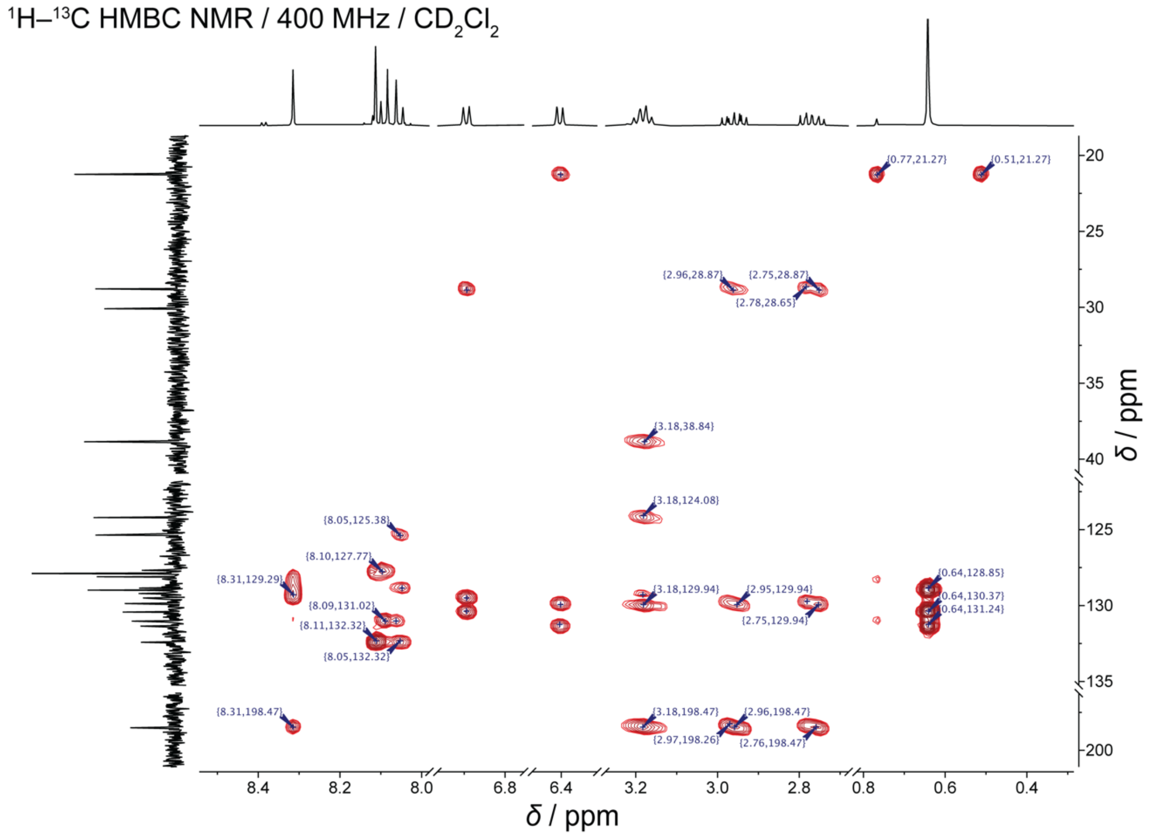

Figure S24: ^1H–^{13}C HMBC spectrum of **5**.

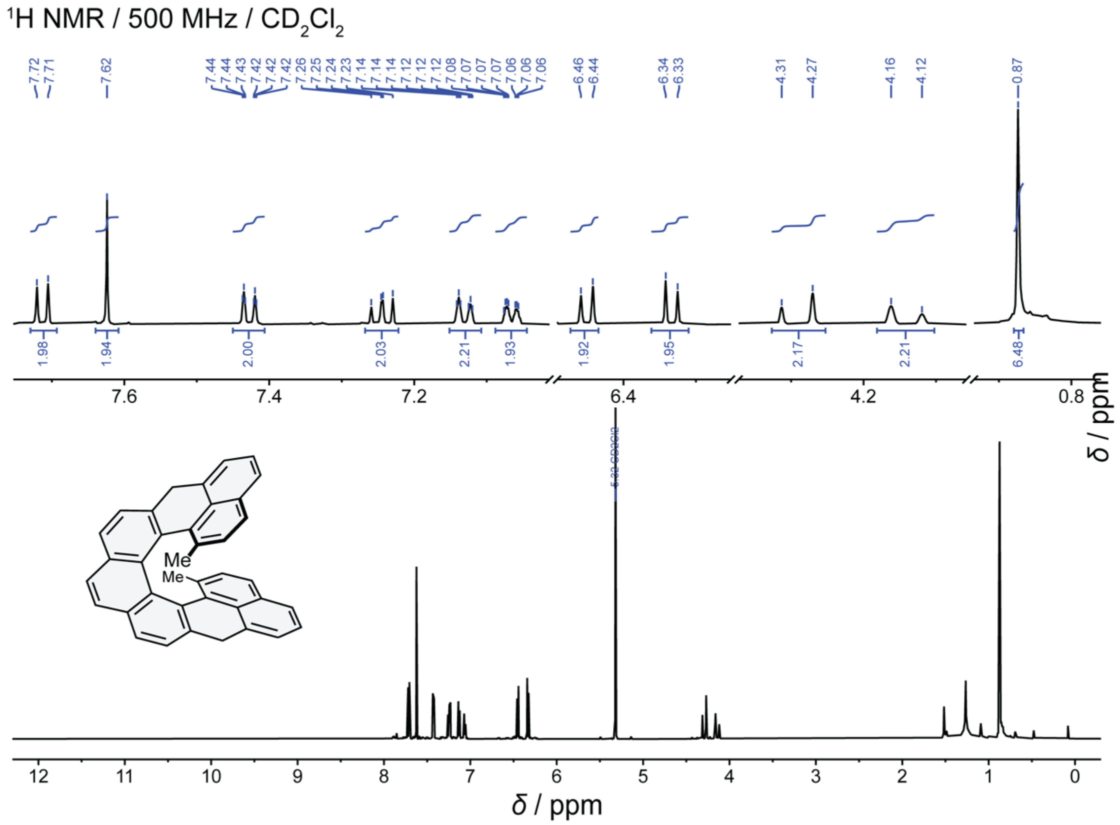

Figure S25: ^{1}H NMR spectrum of 2*H*-**DMNC**.

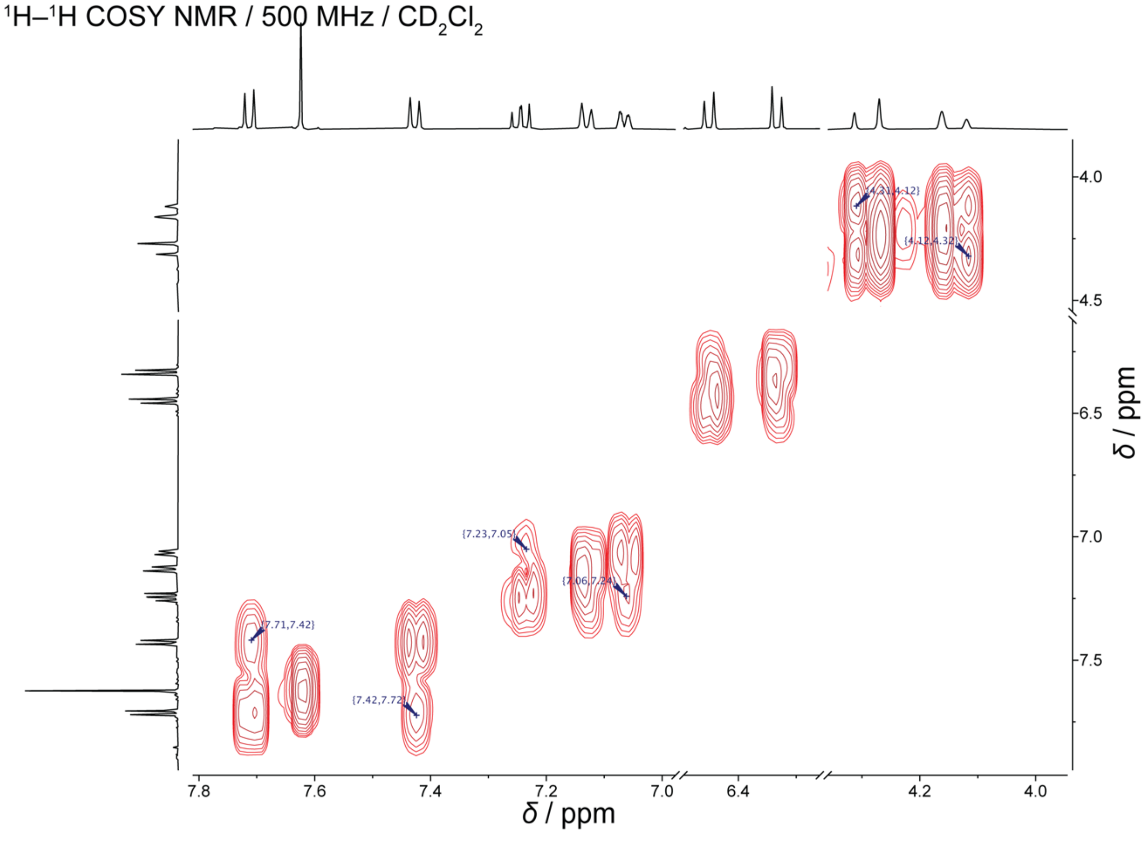

Figure S26: ^{1}H–^{1}H COSY spectrum of 2*H*-**DMNC**.

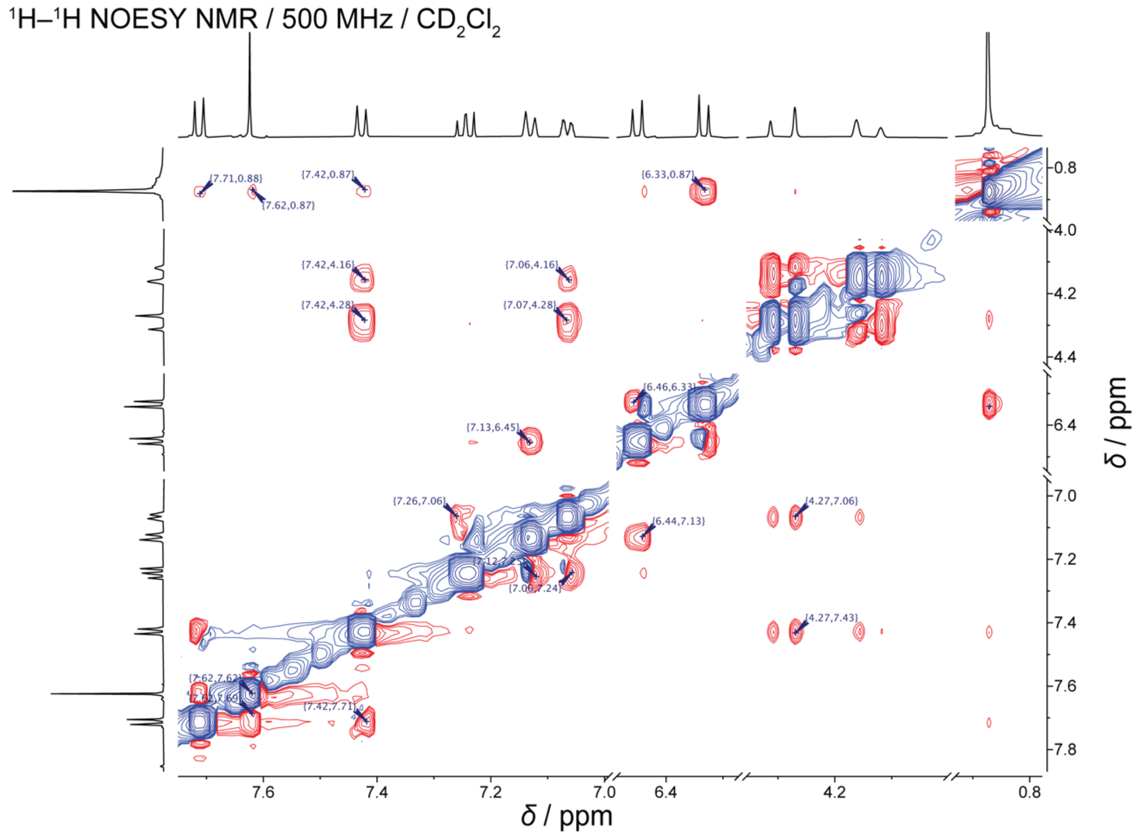

Figure S27: ^{1}H–^{1}H NOESY spectrum of 2*H*-**DMNC**.

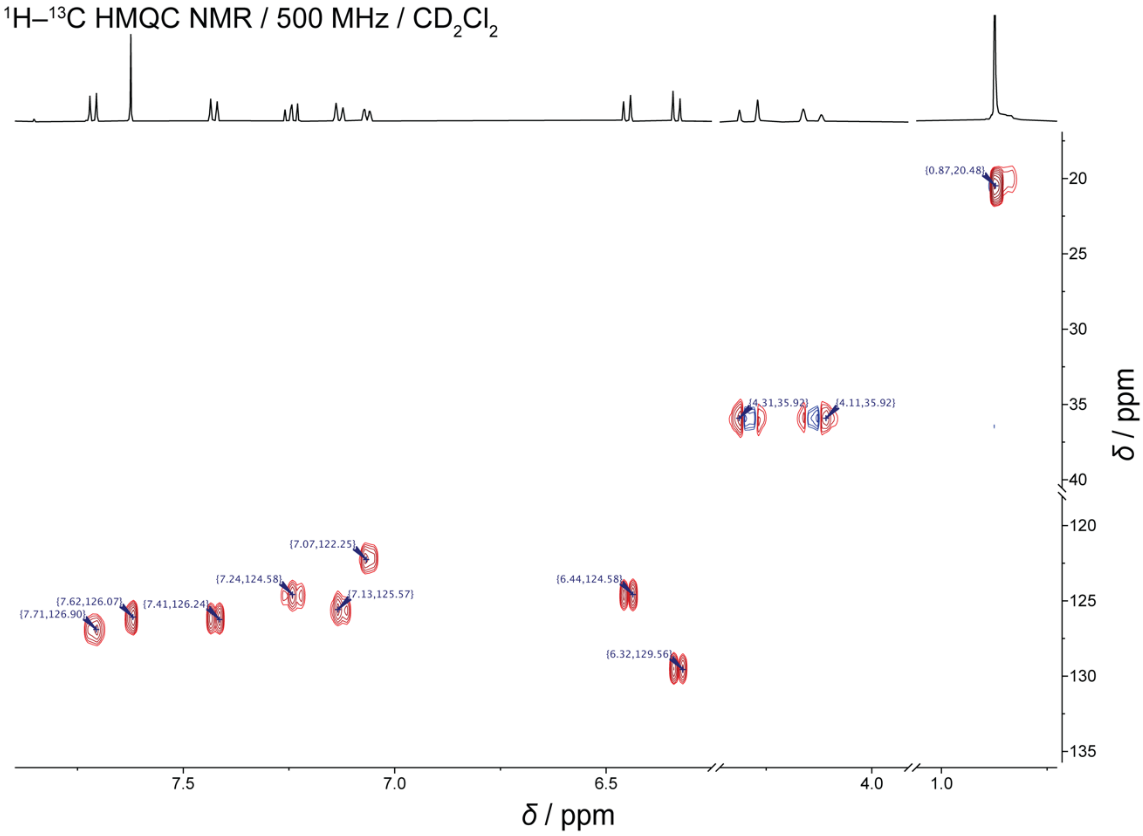

Figure S28: ^{1}H–^{13}C HMQC spectrum of 2*H*-**DMNC**.

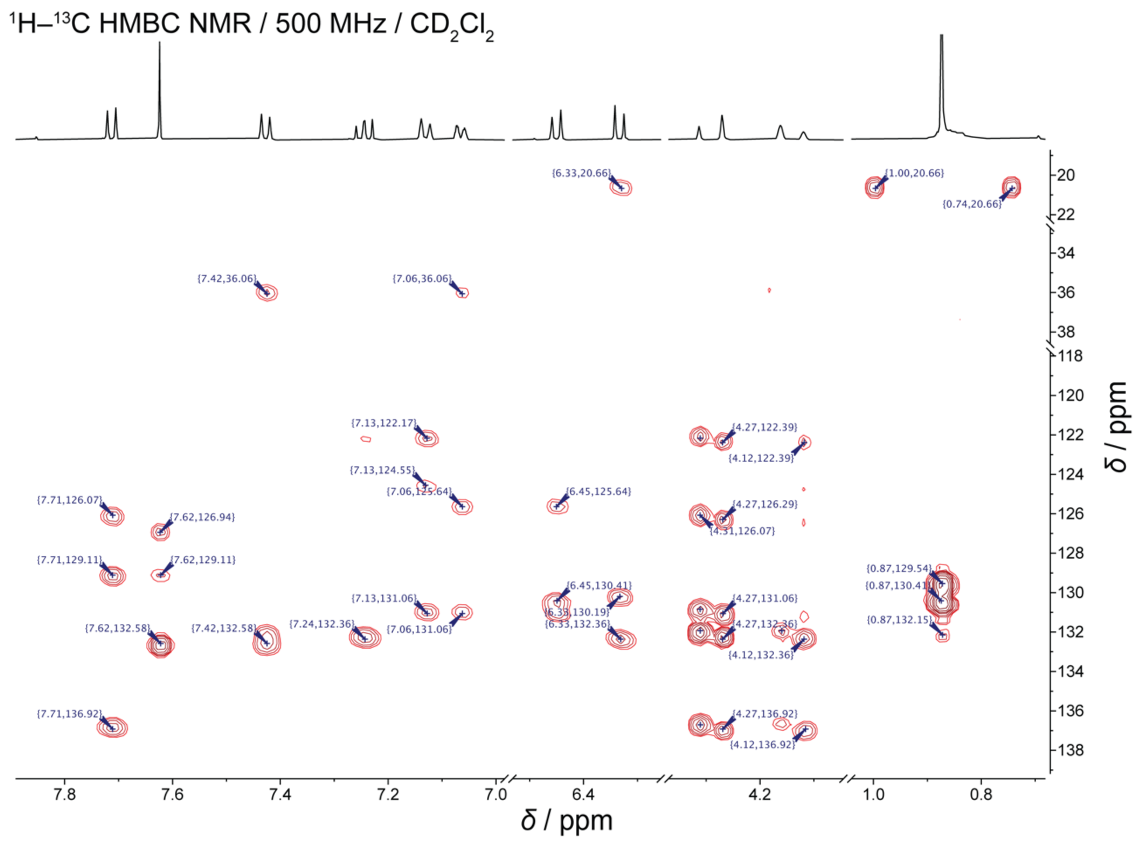

Figure S29: ^1H–^{13}C HMBC spectrum of 2*H*-**DMNC**.

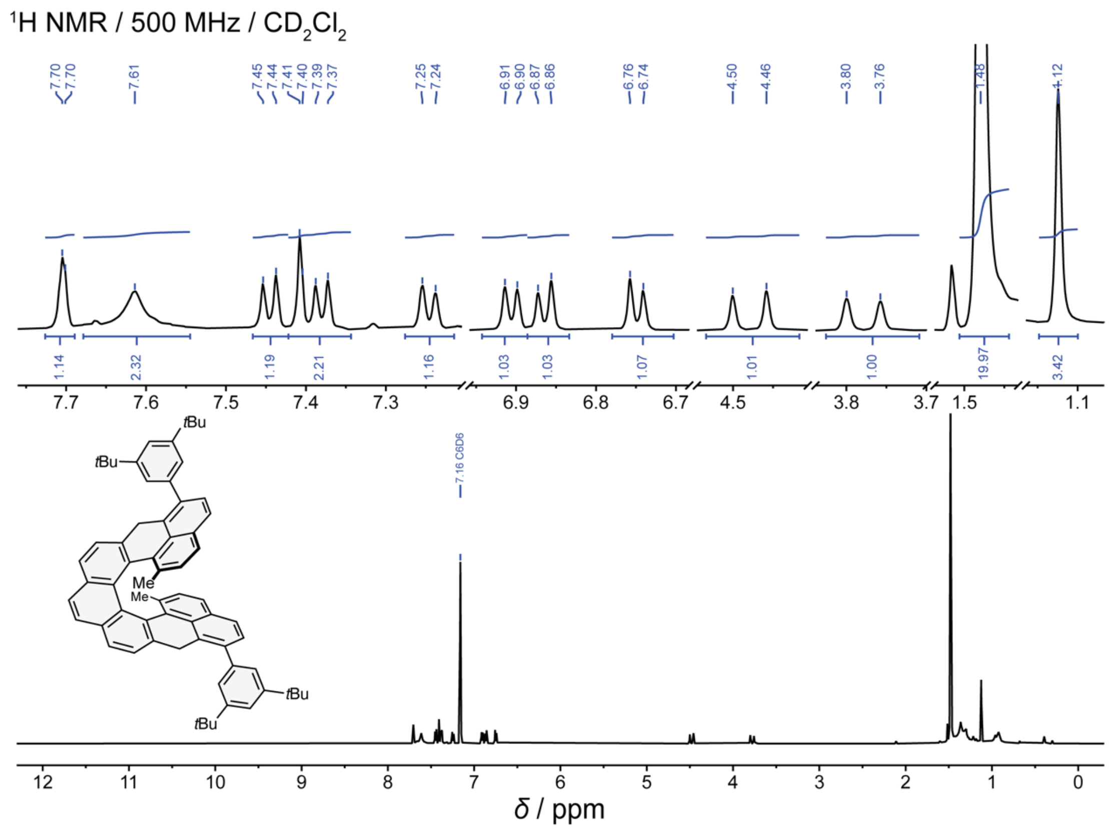

Figure S30: ^1H NMR spectrum of 2*H*-**DMNC-Ar**.

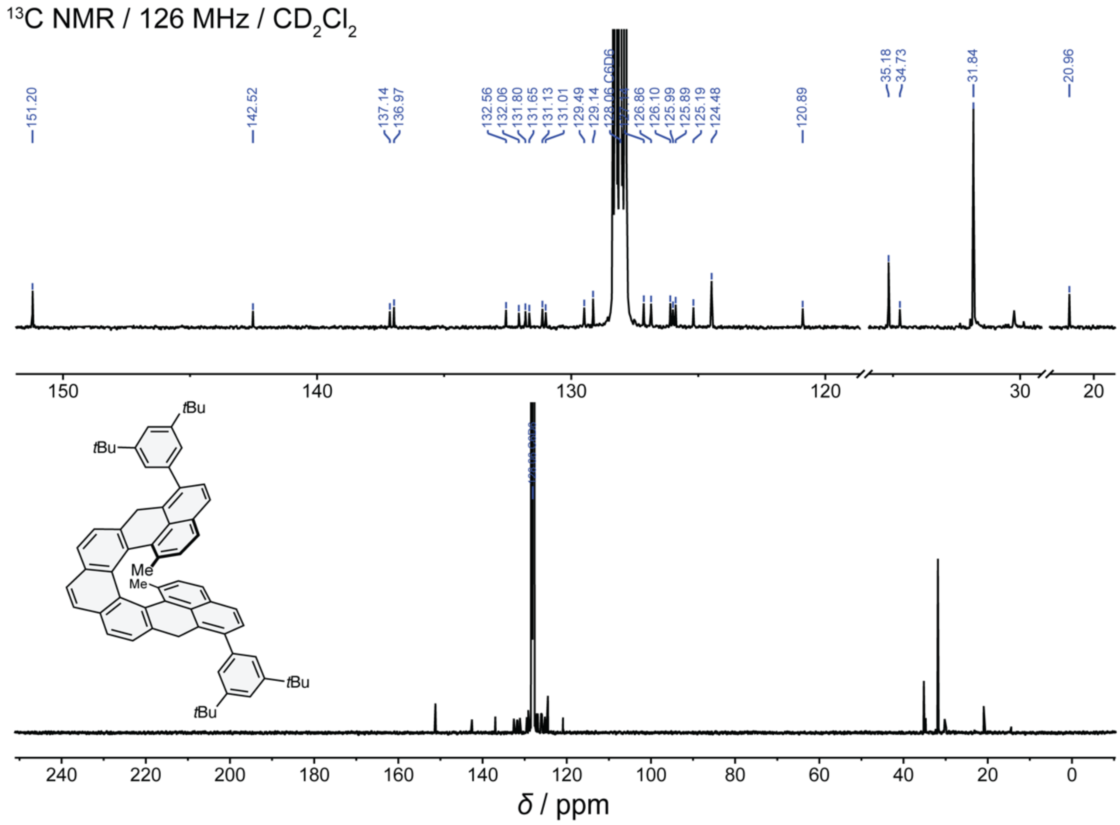

Figure S31: ^{13}C NMR spectrum of 2*H*-**DMNC-Ar**.

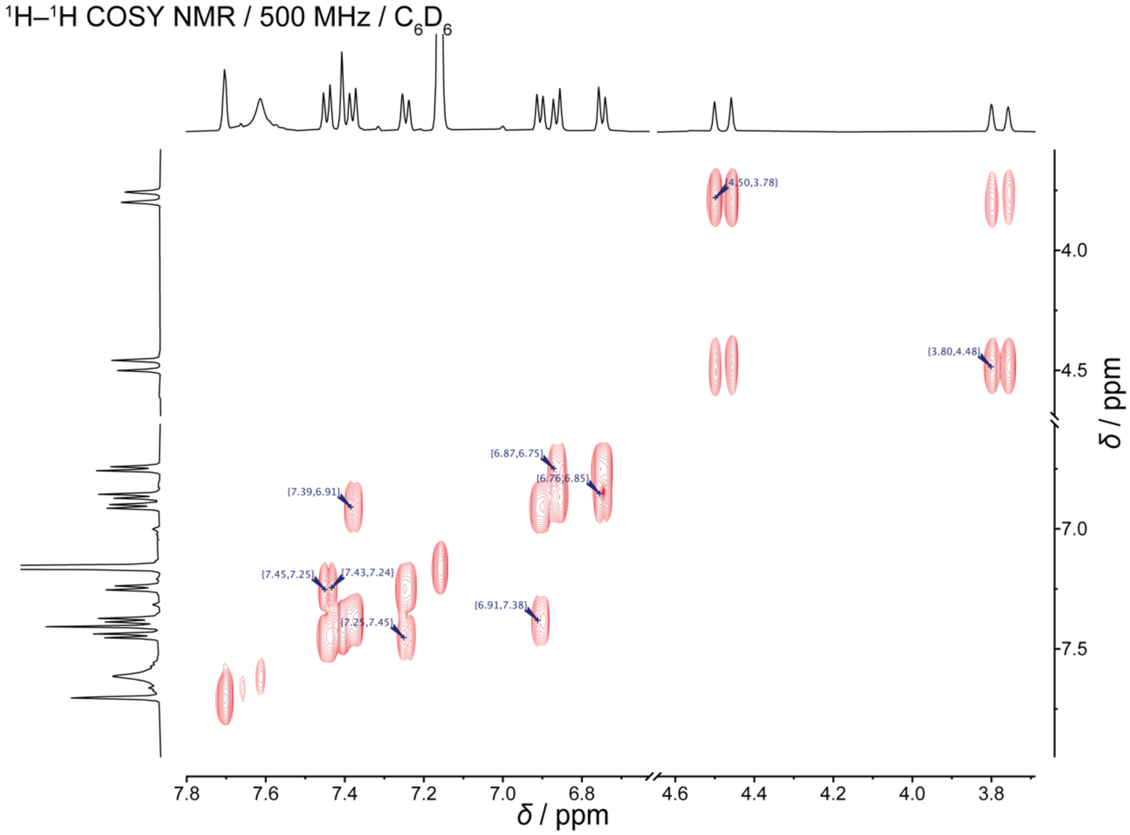

Figure S32: ^{1}H–^{1}H COSY NMR spectrum of 2*H*-**DMNC-Ar**.

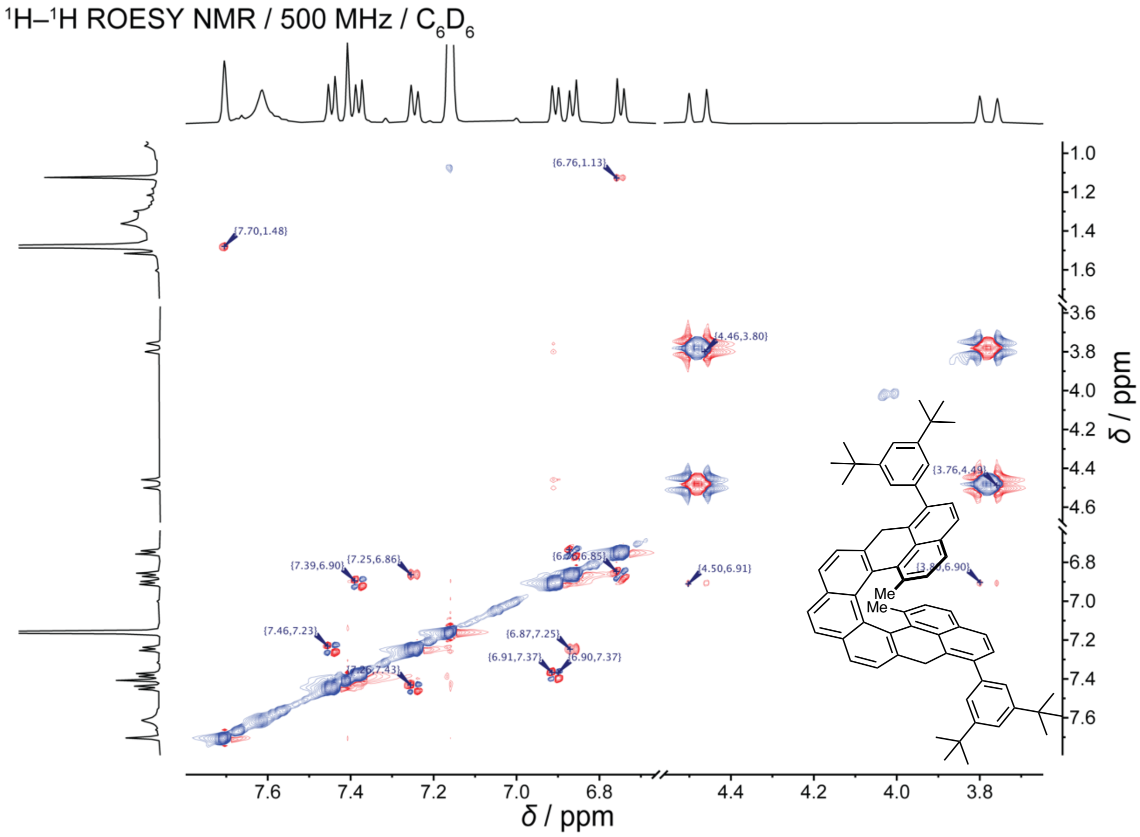

Figure S33: ^{1}H–^{1}H ROESY NMR spectrum of 2*H*-**DMNC-Ar**.

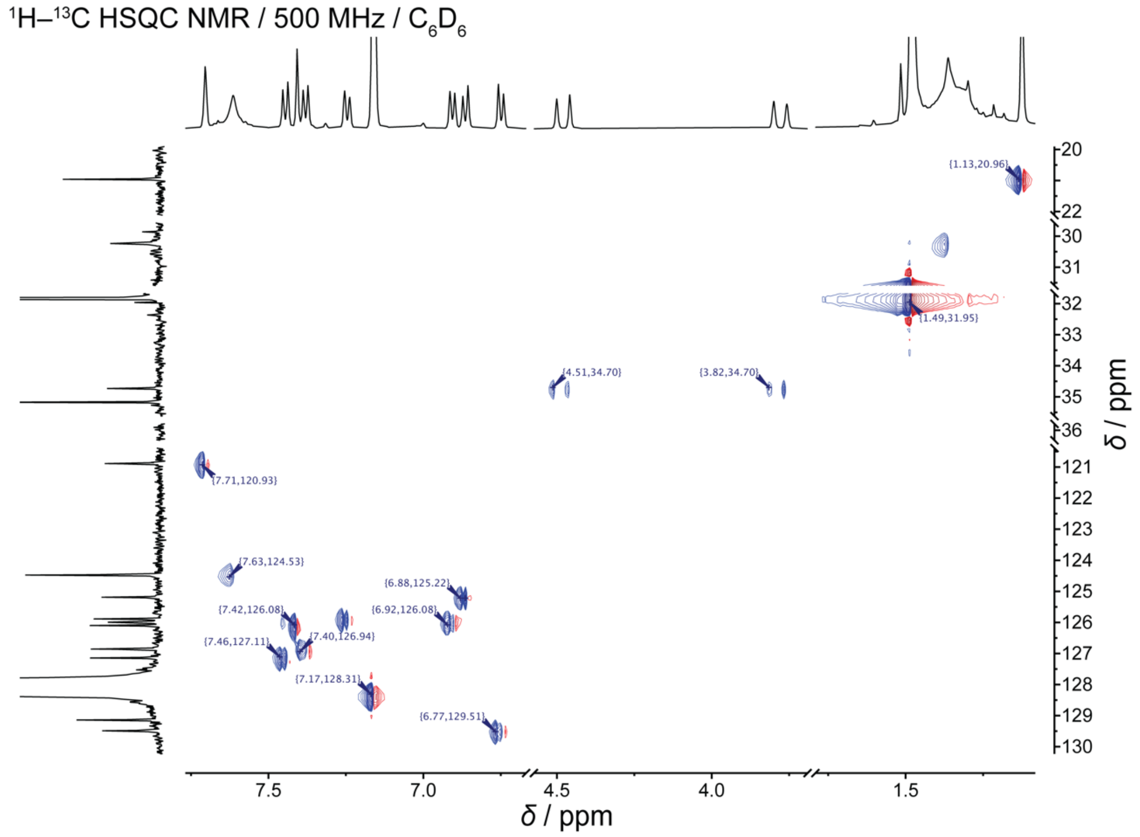

Figure S34: ^{1}H–^{13}C HSQC NMR spectrum of 2*H*-**DMNC-Ar**.

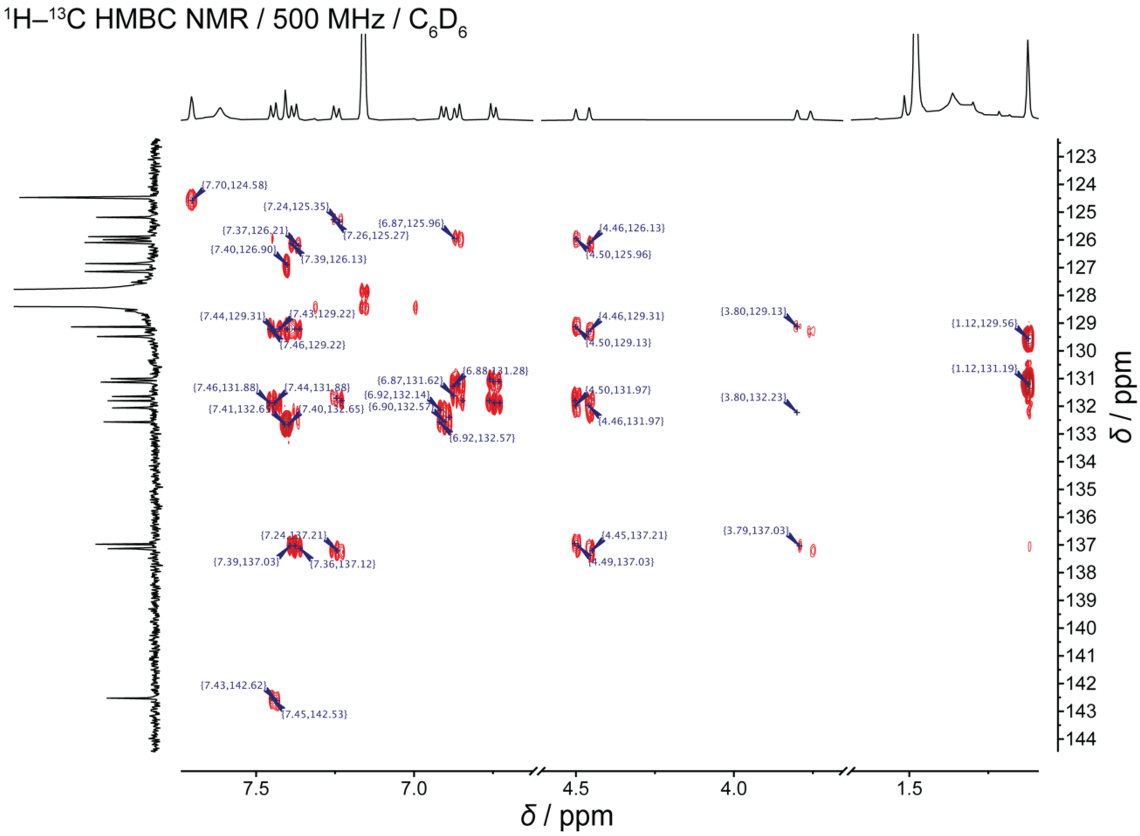

Figure S35: ^{1}H–^{13}C HMBC NMR spectrum of 2*H*-**DMNC-Ar**.

HR-MS Data

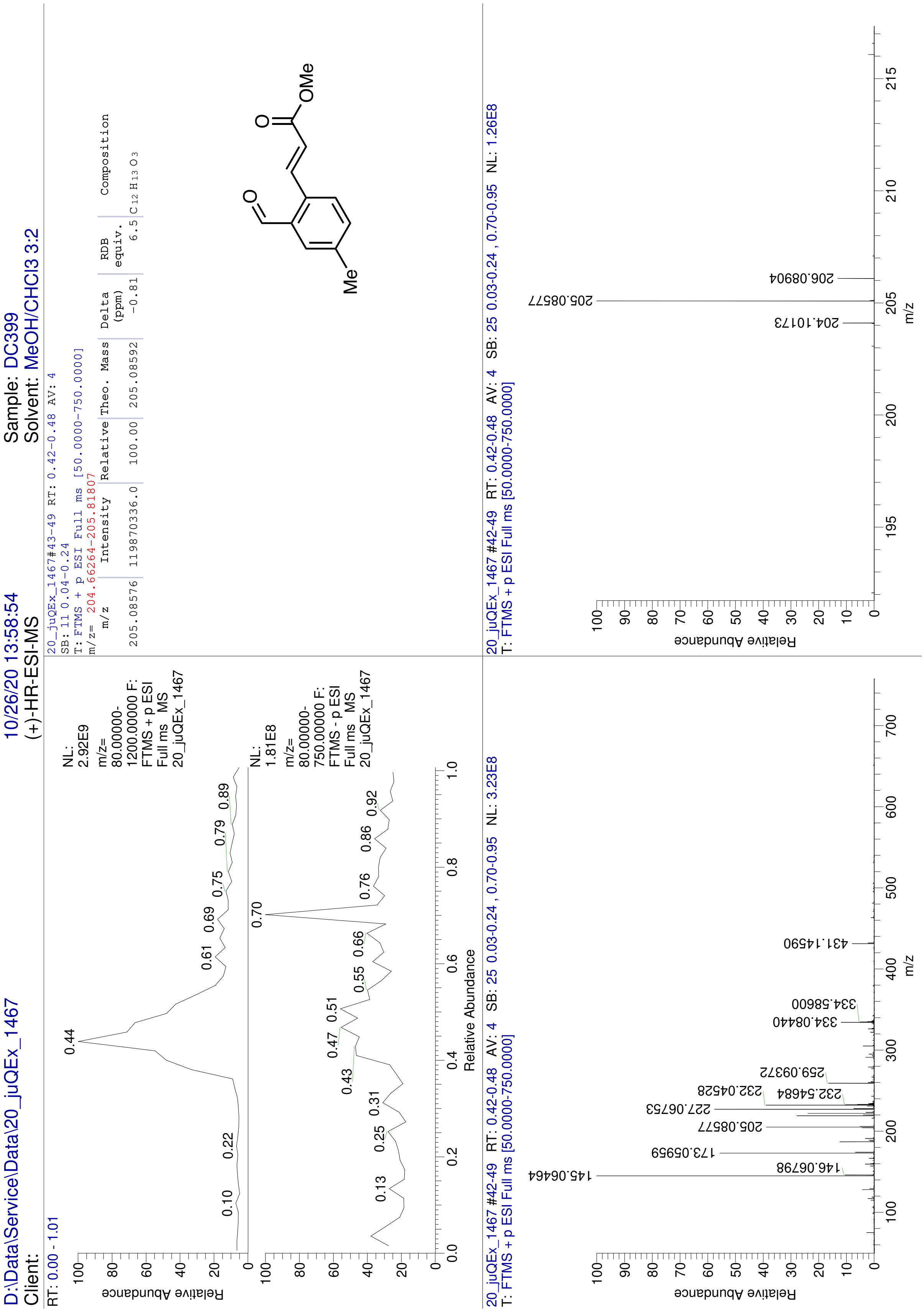

Figure S36: (+)-HR-ESI-MS of **6**.

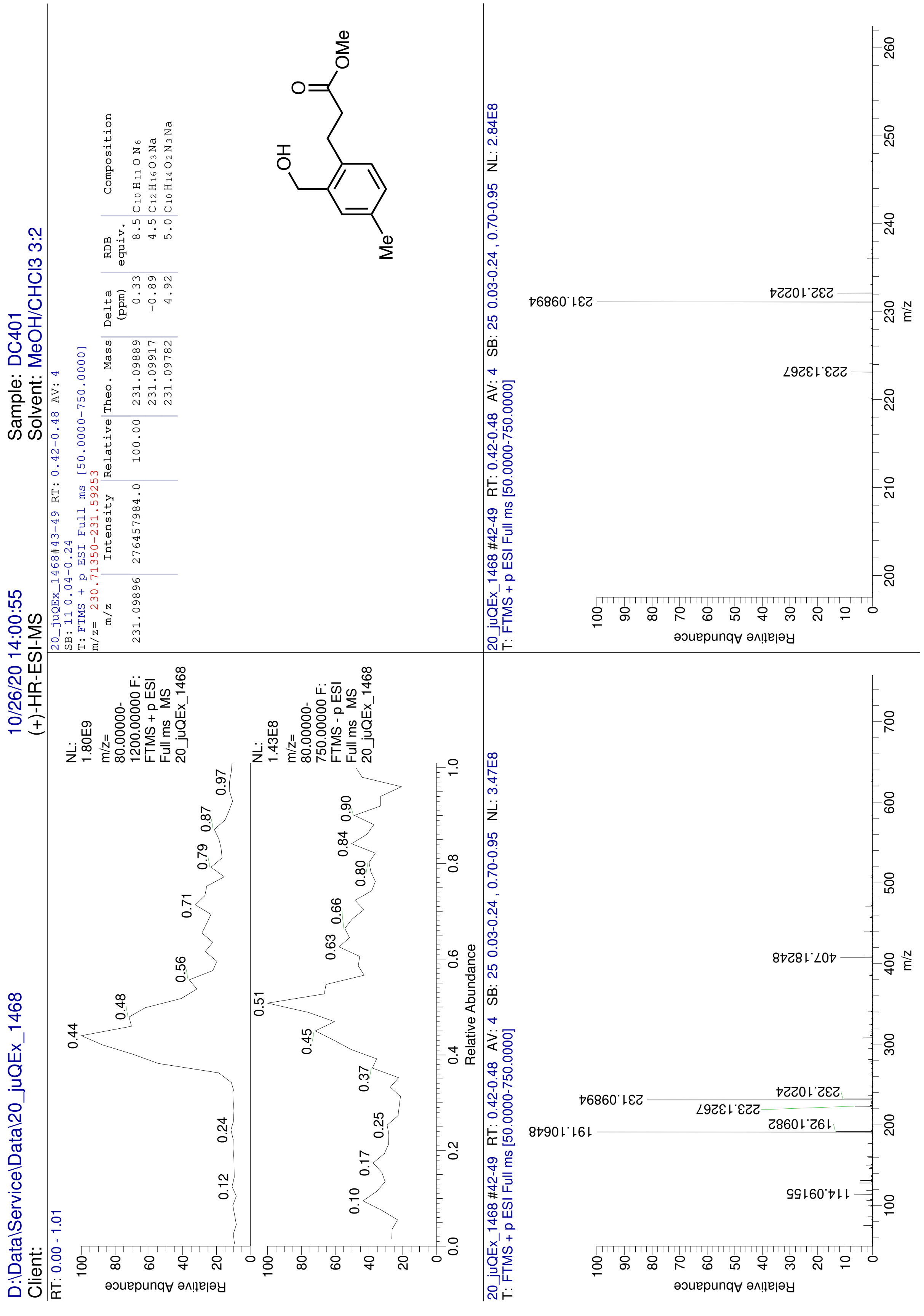

Figure S37: (+)-HR-ESI-MS of **7**.

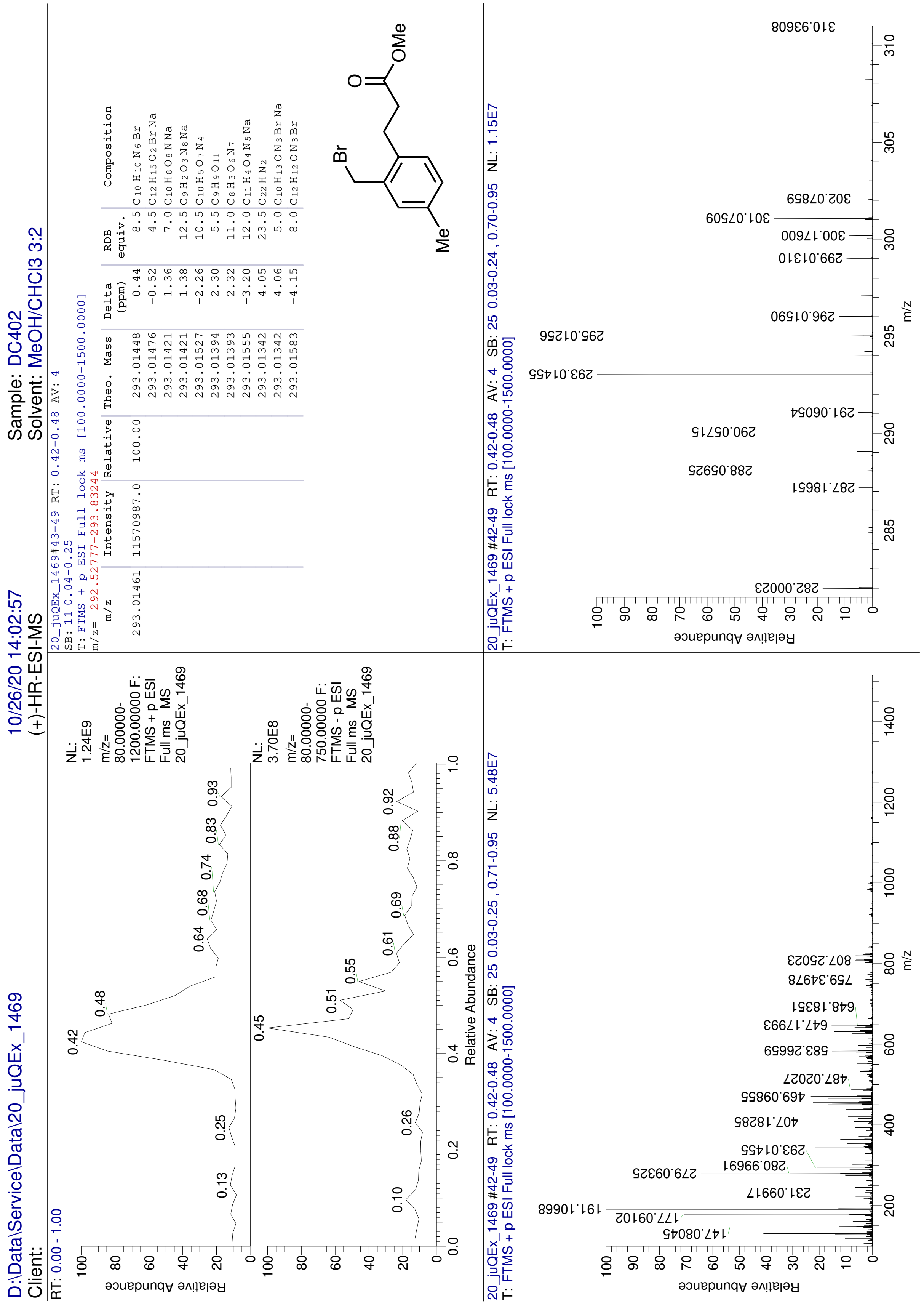

Figure S38: (+)-HR-ESI-MS of **8**.

HR-ESI Report

Analysis Info

Analysis Name	D:\Data\UZH_Data\Service\Data\20_juhres_0231.d
Method	Service_Syringe_Pump_Low_Mass_Range_pos.m
Sample Name	DC403
Comment	Solvent: MeOH + NaI<br>Client: Cavlovic

Acquisition Date	10/28/2020 3:33:25 PM	
Operator	Demo User	
Instrument	timsTOF Pro	1854399.00195

Acquisition Parameter

Source Type	ESI	Ion Polarity	Positive	Set Nebulizer	0.4 Bar
Focus	Active			Set Dry Heater	180 °C
Scan Begin	50 m/z	Set Capillary	2000 V	Set Dry Gas	4.0 l/min
Scan End	2000 m/z	Set End Plate Offset	-500 V	Set Divert Valve	Source

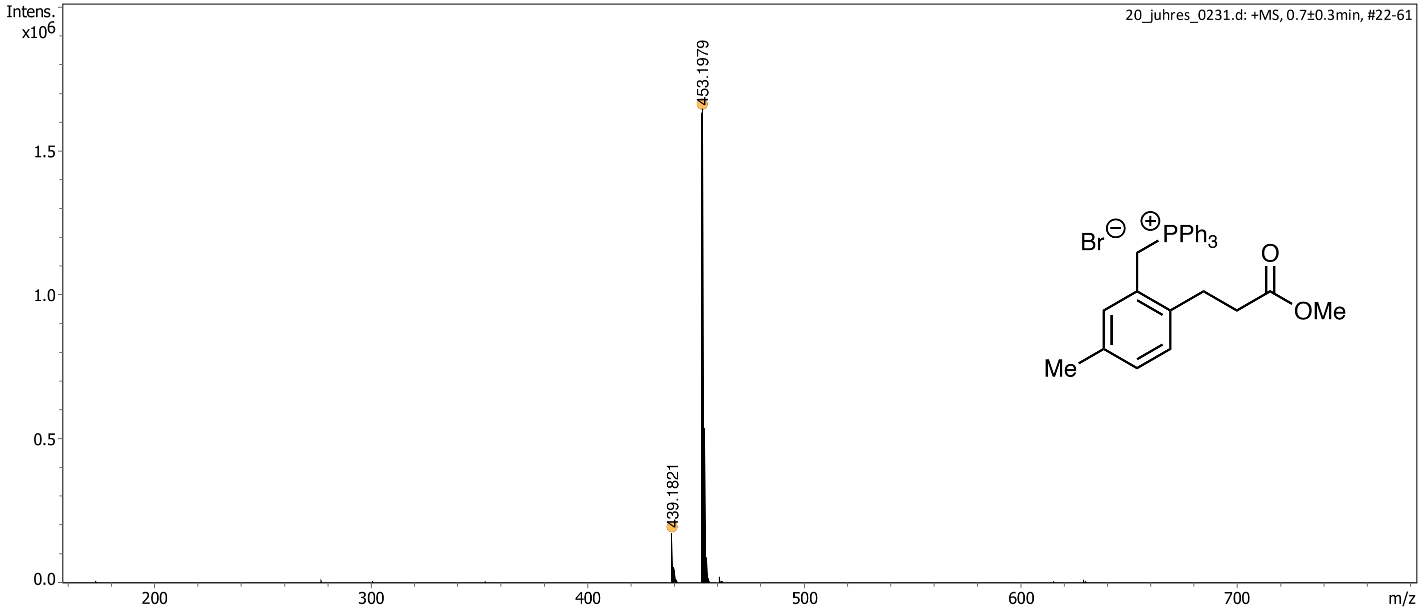

Bruker Compass DataAnalysis 5.3 printed: 10/28/2020 3:49:13 PM by: demo

Figure S39: (+)-HR-ESI-MS of **2**.

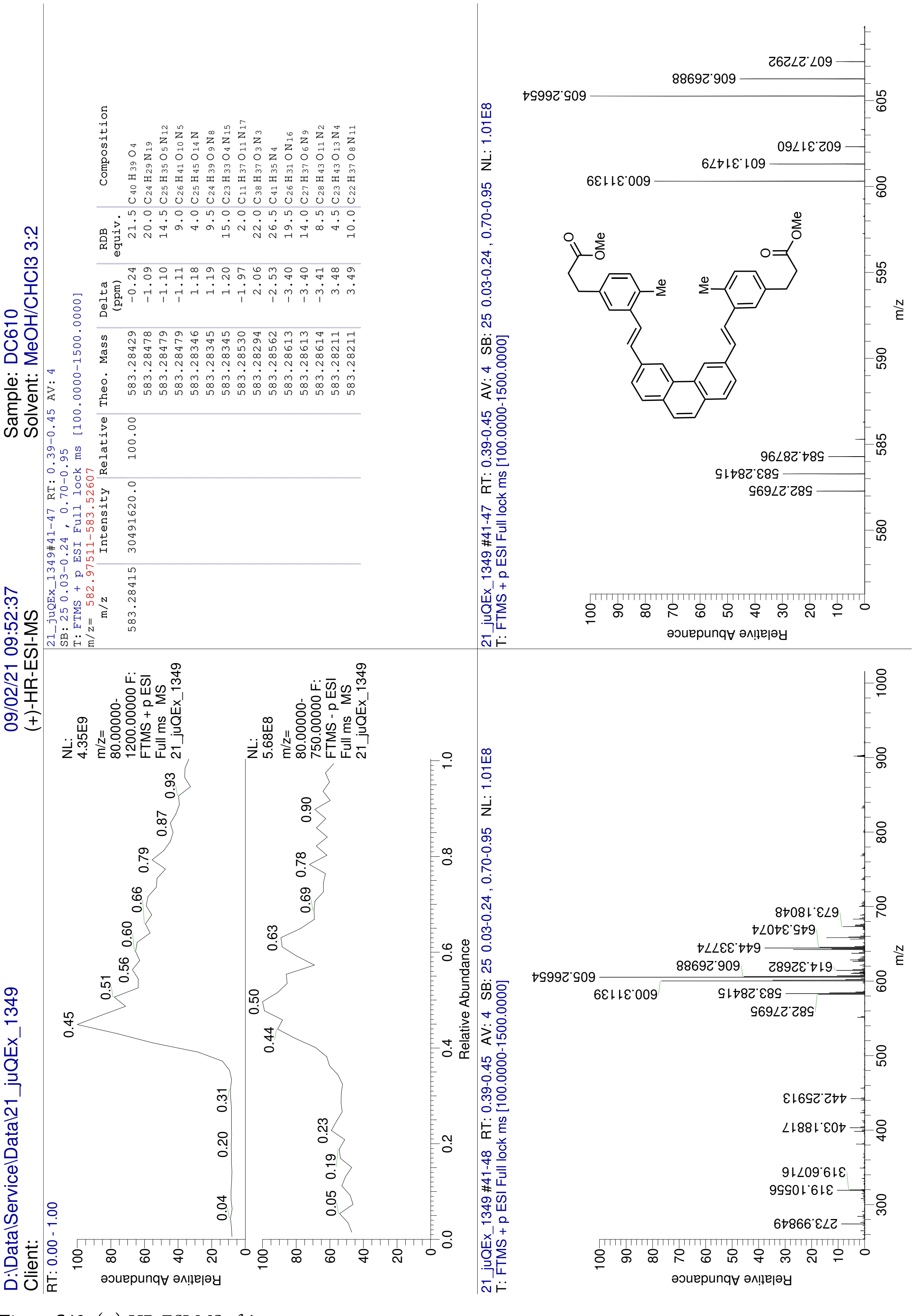

m/z	Intensity	Relative	Theo. Mass	Delta (ppm)	RDB equiv.	Composition
583.28415	30491620.0	100.00	583.28429	-0.24	21.5	$C_{40}H_{39}O_4$
			583.28478	-1.09	20.0	$C_{24}H_{29}N_{19}$
			583.28479	-1.10	14.5	$C_{25}H_{35}O_5N_{12}$
			583.28479	-1.11	9.0	$C_{26}H_{41}O_{10}N_5$
			583.28346	1.18	4.0	$C_{25}H_{45}O_{14}N$
			583.28345	1.19	9.5	$C_{24}H_{39}O_9N_8$
			583.28345	1.20	15.0	$C_{23}H_{33}O_4N_{15}$
			583.28530	-1.97	2.0	$C_{11}H_{37}O_{11}N_{17}$
			583.28294	2.06	22.0	$C_{38}H_{37}O_3N_3$
			583.28562	-2.53	26.5	$C_{41}H_{35}N_4$
			583.28613	-3.40	19.5	$C_{26}H_{31}ON_{16}$
			583.28613	-3.40	14.0	$C_{27}H_{37}O_6N_9$
			583.28614	-3.41	8.5	$C_{28}H_{43}O_{11}N_2$
			583.28211	3.48	4.5	$C_{23}H_{43}O_{13}N_4$
			583.28211	3.49	10.0	$C_{22}H_{37}O_8N_{11}$

Figure S40: (+)-HR-ESI-MS of **4**.

Mass Spectrum SmartFormula Report

Analysis Info

Acquisition Date 23.12.2016 14:56:50

Analysis Name	E:\new acq data for data analysis\PR249 002.d
Method	hn Direct_Infusion_pos mode_75-1700 mid 4eV.m
Sample Name	Prince Ravat
Comment	PR249. ca. 50 ug/ml MeCN

Operator	hn
Instrument / Ser#	maXis 4G 21243

Acquisition Parameter

Source Type	ESI	Ion Polarity	Positive	Set Nebulizer	0.4 Bar
Focus	Not active	Set Capillary	3600 V	Set Dry Heater	180 °C
Scan Begin	75 m/z	Set End Plate Offset	-500 V	Set Dry Gas	4.0 l/min
Scan End	1700 m/z	Collision Energy	8.0 eV	Set Ion Energy (MS only)	4.0 eV

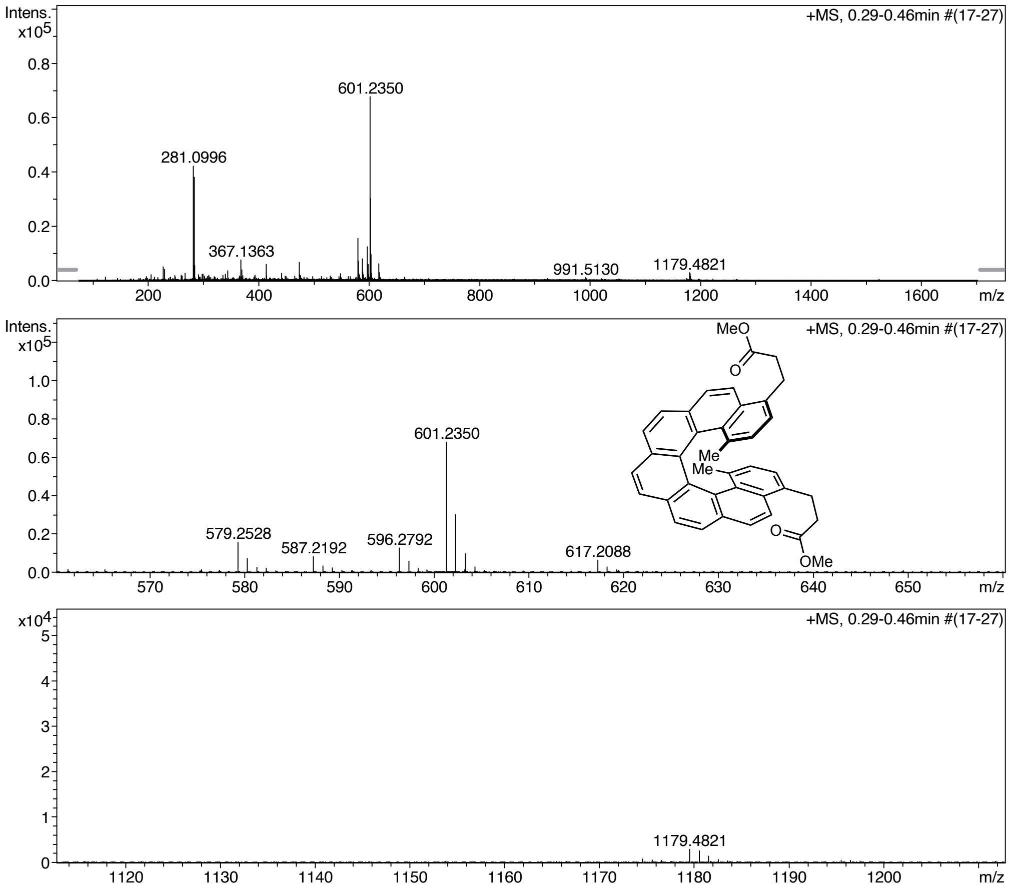

Meas. m/z	#	Formula	Score	m/z	err [mDa]	err [ppm]	mSigma	rdb	e⁻ Conf	z
579.2528	1	C 40 H 35 O 4	100.00	579.2530	0.2	0.3	61.4	23.5	even	1+
596.2792	1	C 40 H 38 N O 4	100.00	596.2795	0.3	0.6	40.9	22.5	even	
601.2350	1	C 40 H 34 Na O 4	100.00	601.2349	-0.1	-0.1	23.6	23.5	even	
617.2088	1	C 40 H 34 K O 4	100.00	617.2089	0.1	0.1	18.8	23.5	even	
1179.4821	1	C 80 H 68 Na O 8	100.00	1179.4806	-1.4	-1.2	66.5	46.5	even	

Bruker Compass DataAnalysis 4.0 printed: 23.12.2016 15:04:47 Page 1 of 1

Figure S41: (+)-HR-ESI-MS of **9**.

Mass Spectrum SmartFormula Report

Analysis Info

Analysis Name	E:\new acq data for data analysis\PR250 001.d	Acquisition Date	23.12.2016 13:27:11
Method	hn Direct_Infusion_pos mode_75-1700 mid 4eV.m	Operator	hn
Sample Name	Prince Ravat	Instrument / Ser#	maXis 4G 21243
Comment	PR250. ca. 50 ug/ml MeCN		

Acquisition Parameter

Source Type	ESI	Ion Polarity	Positive	Set Nebulizer	0.4 Bar
Focus	Not active	Set Capillary	3600 V	Set Dry Heater	180 °C
Scan Begin	75 m/z	Set End Plate Offset	-500 V	Set Dry Gas	4.0 l/min
Scan End	1700 m/z	Collision Energy	8.0 eV	Set Ion Energy (MS only)	4.0 eV

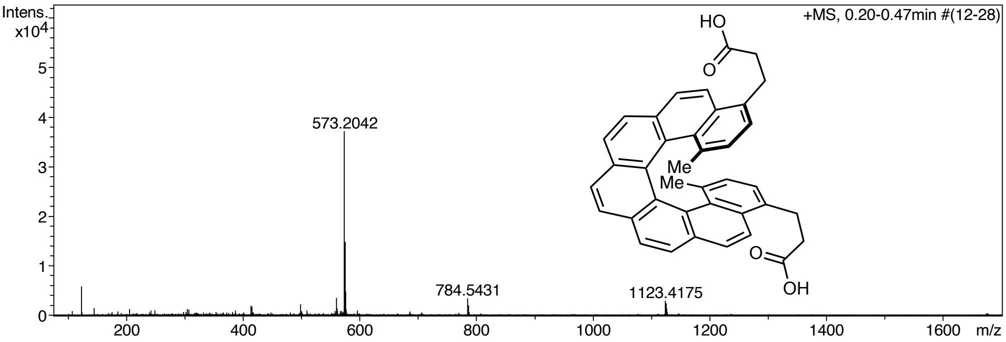

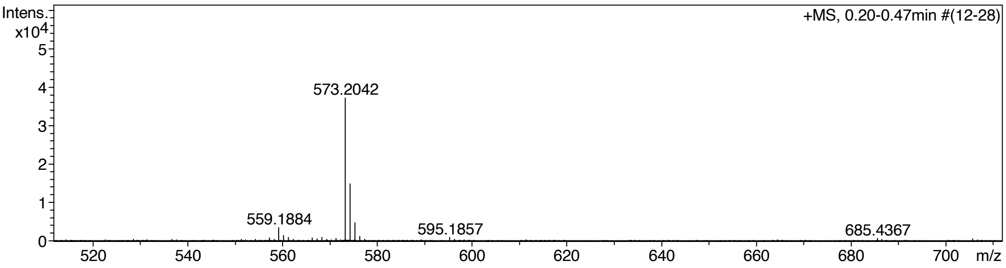

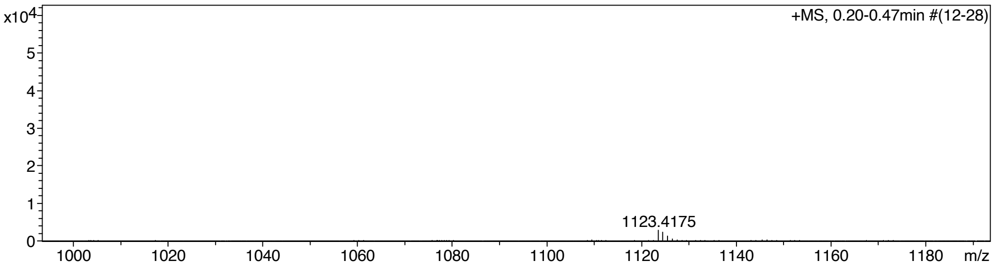

Meas. m/z	#	Formula	Score	m/z	err [mDa]	err [ppm]	mSigma	rdb	e⁻ Conf	z
573.2042	1	C 38 H 30 Na O 4	100.00	573.2036	-0.6	-1.1	20.6	23.5	even	1+
595.1857	1	C 38 H 29 Na 2 O 4	100.00	595.1856	-0.1	-0.2	55.0	23.5	even	
1123.4175	1	C 76 H 60 Na O 8	100.00	1123.4180	0.6	0.5	64.5	46.5	even	

Bruker Compass DataAnalysis 4.0 printed: 23.12.2016 13:39:54 Page 1 of 1

Figure S42: (+)-HR-ESI-MS of **10**.

Mass Spectrum SmartFormula Report

Analysis Info

Analysis Name	E:\new acq data for data analysis\PR251 002.d	Acquisition Date	03.01.2017 17:27:40
Method	hn Direct_Infusion_pos mode_75-2000 higher 4eV.m	Operator	hn
Sample Name	Prince Ravat	Instrument / Ser#	maXis 4G 21243
Comment	PR251, ca. 50 ug /ml MeCN, with MeCN/TFA		

Acquisition Parameter

Source Type	ESI	Ion Polarity	Positive	Set Nebulizer	0.4 Bar
Focus	Not active	Set Capillary	3600 V	Set Dry Heater	180 °C
Scan Begin	75 m/z	Set End Plate Offset	-500 V	Set Dry Gas	4.0 l/min
Scan End	2000 m/z	Collision Energy	8.0 eV	Set Ion Energy (MS only)	4.0 eV

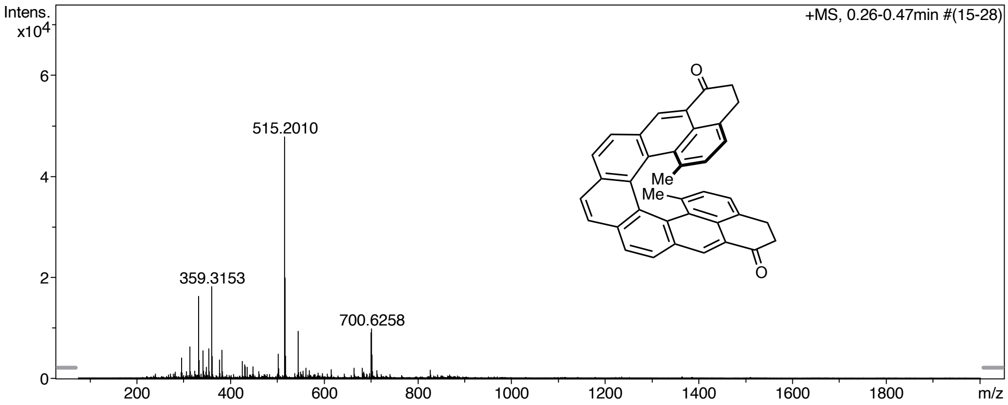

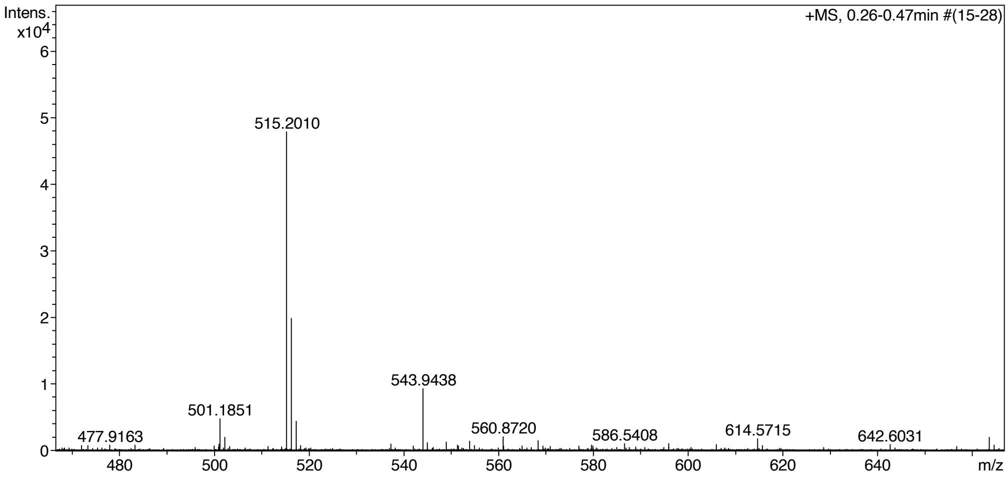

Meas. m/z	#	Formula	Score	m/z	err [mDa]	err [ppm]	mSigma	rdb	e⁻ Conf	z
515.2010	1	C 38 H 27 O 2	100.00	515.2006	-0.5	-0.9	3.5	25.5	even	1+

Bruker Compass DataAnalysis 4.0 printed: 03.01.2017 17:33:04 Page 1 of 1

Figure S43: (+)-HR-ESI-MS of **5**.

Mass Spectrum SmartFormula Report

Analysis Info

Analysis Name	E:\new acq data for data analysis\PR252 001.d	Acquisition Date	03.01.2017 17:52:29
Method	hn Direct_Infusion_pos mode_75-2000 higher 4eV.m	Operator	hn
Sample Name	Prince Ravat	Instrument / Ser#	maXis 4G 21243
Comment	PR252, ca. 50 ug /ml MeCN		

Acquisition Parameter

Source Type	ESI	Ion Polarity	Positive	Set Nebulizer	0.4 Bar
Focus	Not active	Set Capillary	3600 V	Set Dry Heater	180 °C
Scan Begin	75 m/z	Set End Plate Offset	-500 V	Set Dry Gas	4.0 l/min
Scan End	2000 m/z	Collision Energy	88.0 eV	Set Ion Energy (MS only)	4.0 eV

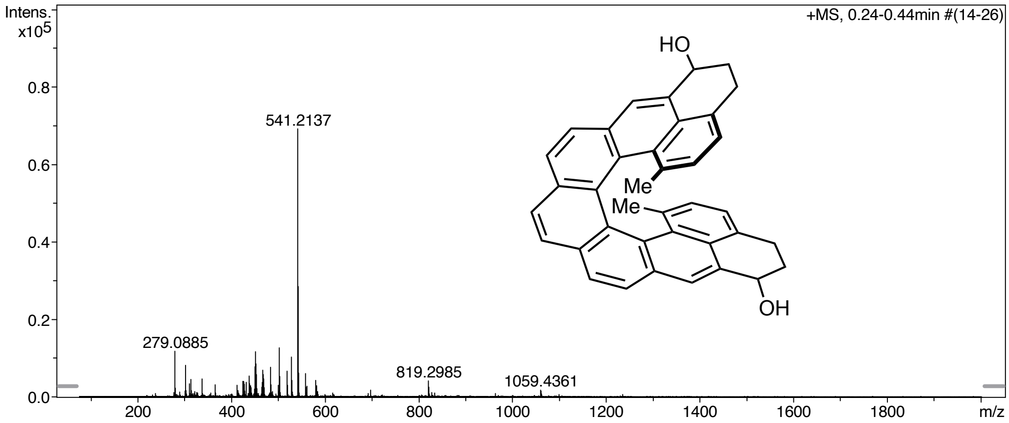

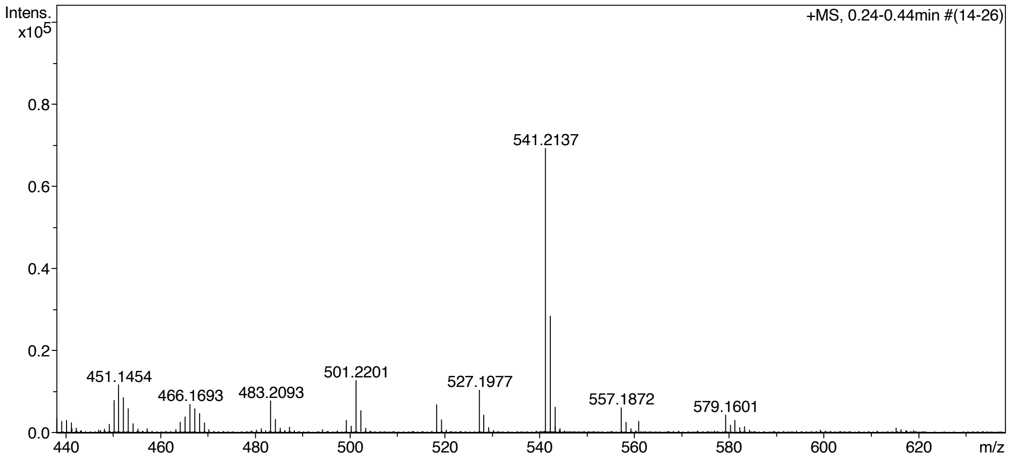

Meas. m/z	#	Formula	Score	m/z	err [mDa]	err [ppm]	mSigma	rdb	e⁻ Conf	z
541.2137	1	C 38 H 30 Na O 2	100.00	541.2138	0.1	0.1	1.3	23.5	even	1+
557.1872	1	C 38 H 30 K O 2	100.00	557.1877	0.5	0.9	8.1	23.5	even	

Bruker Compass DataAnalysis 4.0 printed: 03.01.2017 18:01:15 Page 1 of 1

Figure S44: (+)-HR-ESI-MS of **11**.

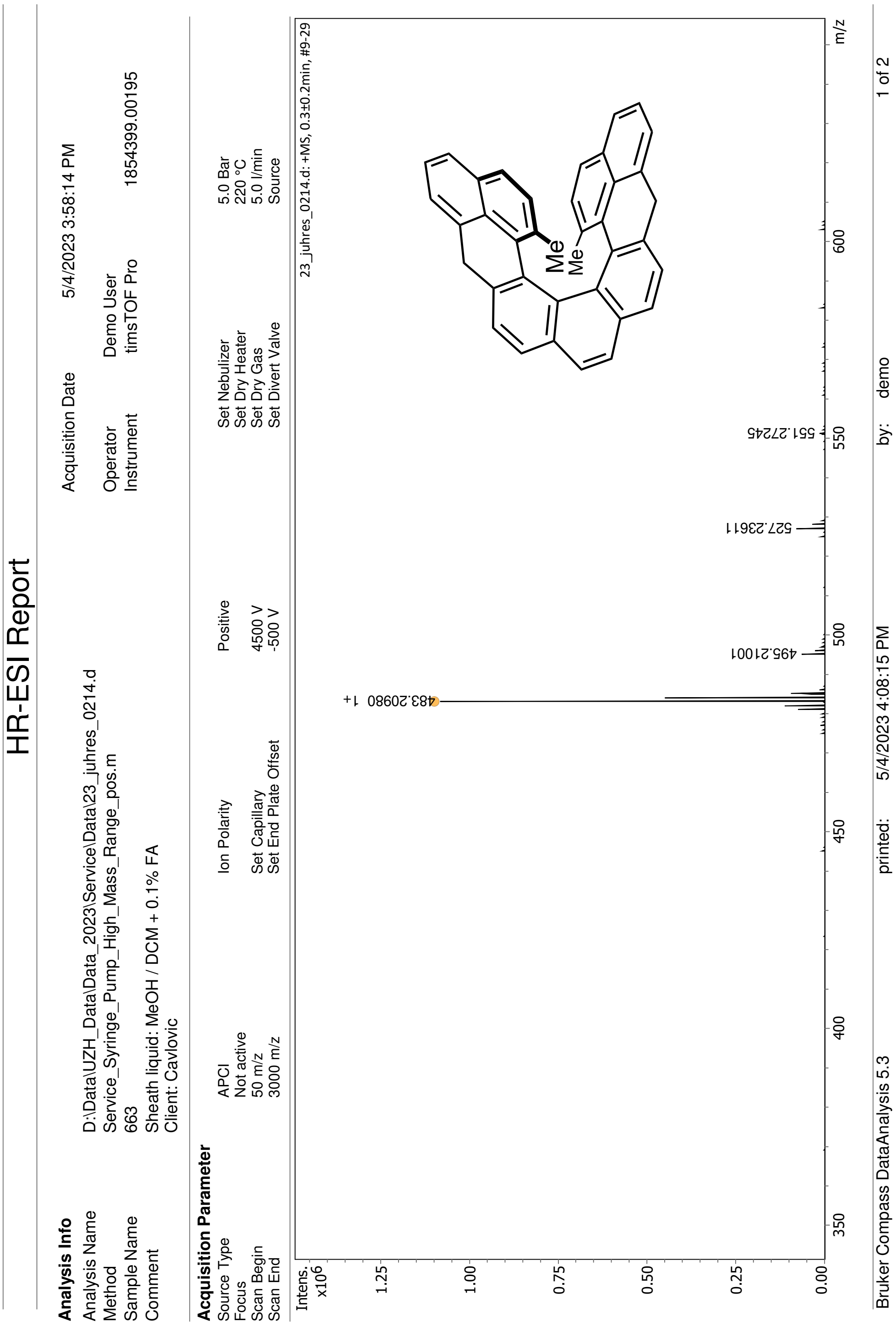

Figure S45: (+)-HR-ESI-MS of 2*H*-**DMNC**.

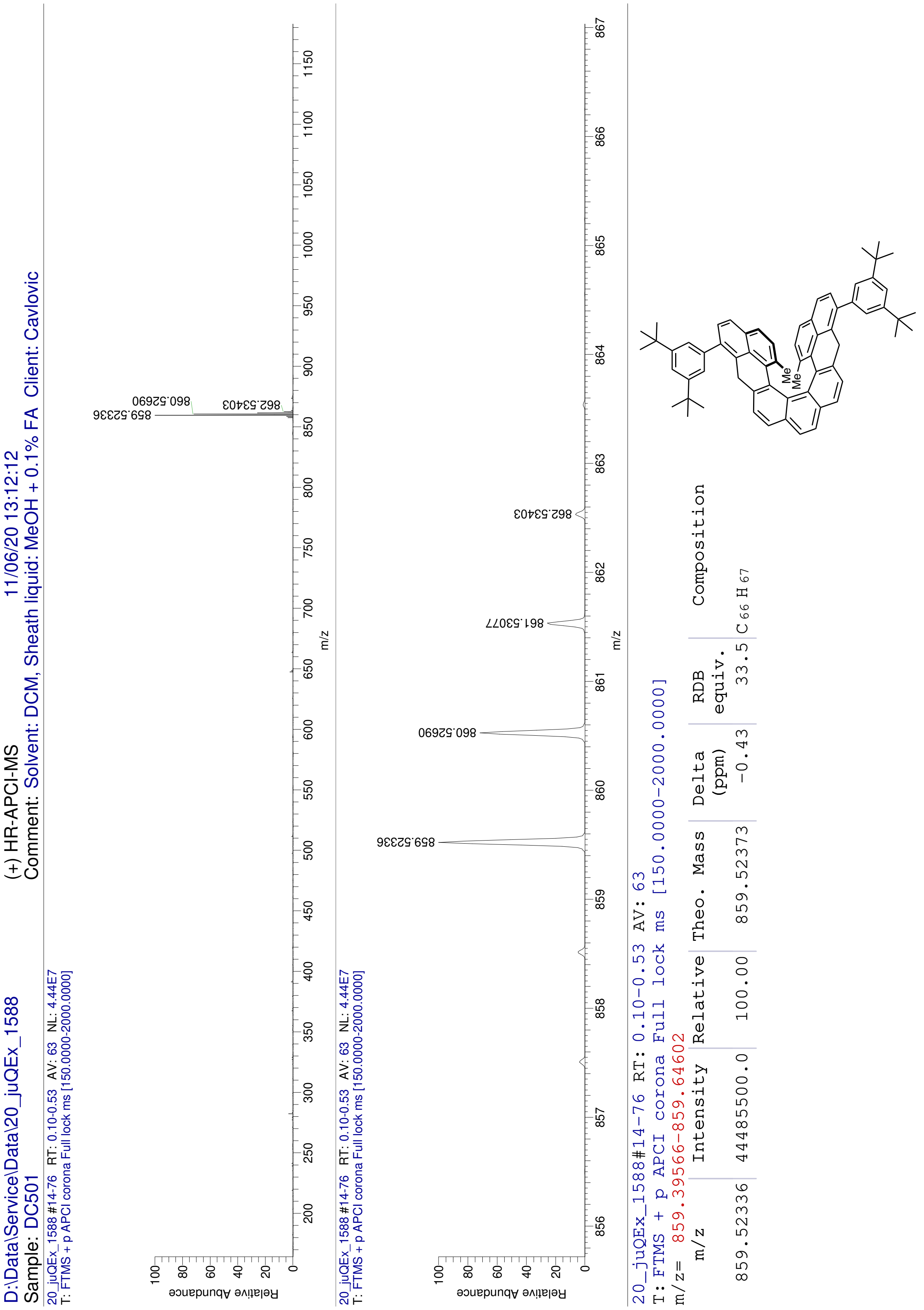

Figure S46: (+)-HR-APCI-MS of 2*H*-**DMNC-Ar**.

D:\Data\Service\Data\22_juQEx_1846 02/09/22 10:29:18
Sample: DC625-ccF1 (+)-HR-APCI-MS Solvent: DCM, Sheath liquid: MeOH /DCM 1:1 + 0.1% FA

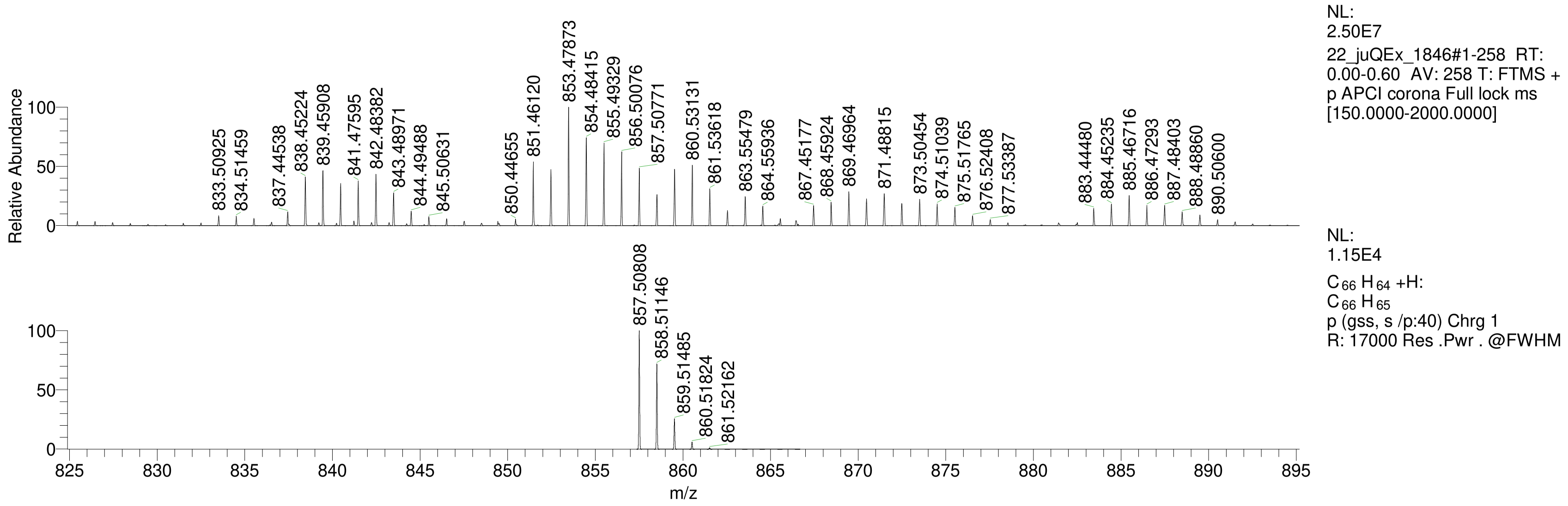

22_juQEx_1846#1-258 RT: 0.00-0.60 AV: 258
T: FTMS + p APCI corona Full lock ms [150.0000-2000.0000]
m/z= 852.88503-857.93754

m/z	Intensity	Relative	Theo. Mass	Delta (ppm)	RDB equiv.	Composition
853.47873	25200868.0	100.00	853.47678	2.29	36.5	$C_{66}H_{61}$
854.48415	18821584.0	74.69	854.48460	-0.53	36.0	$C_{66}H_{62}$
855.49329	17540720.0	69.60	855.49243	1.01	35.5	$C_{66}H_{63}$
856.50076	15929071.0	63.21	856.50025	0.59	35.0	$C_{66}H_{64}$
857.50771	12330738.0	48.93	857.50808	-0.43	34.5	$C_{66}H_{65}$

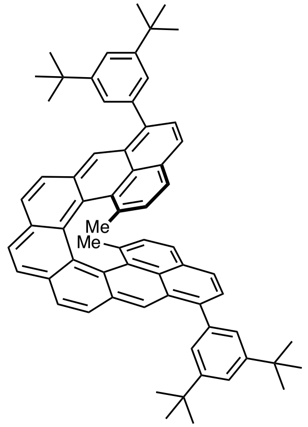

Figure S47: (+)-HR-APCI-MS of **DMNC-Ar**.

EPR Spectroscopy

The EPR spectra (Fig. S47) were recorded in a nitrogen-saturated benzene solution (left), as a solid powder (middle), or in a solid matrix (right) on an X-band bench-top EPR spectrometer (9.48 GHz) using the following instrumental parameters: magnetic field 336–341 mT, scan time 1800 s, modulation amplitude 0.2 mT, microwave power 10 mW. The raw EPR data is available free of charge on a public repository Zenodo (DOI:10.5281/zenodo.7733689).

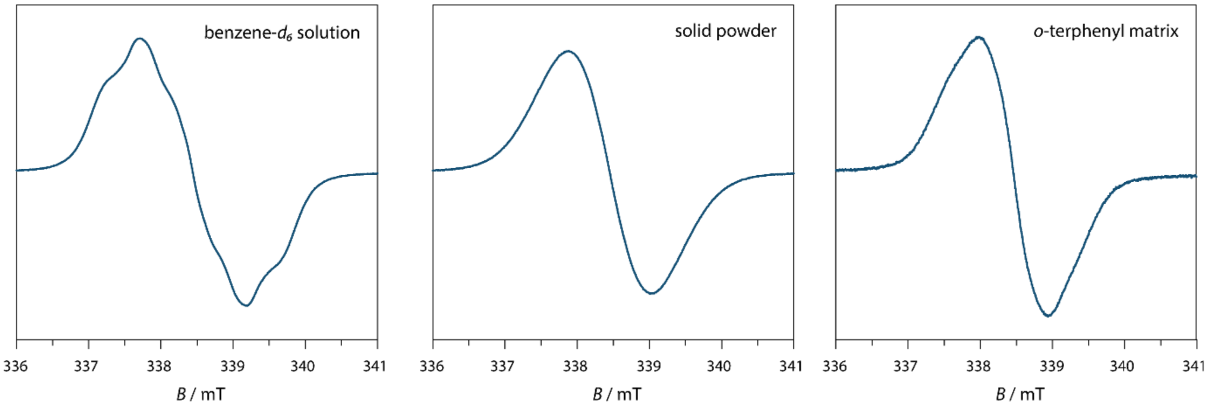

Figure S48: EPR spectra of **DMNC-Ar** in 8.7 mM benzene-d_6 solution (left), as a solid powder (middle), and in an *o*-terphenyl matrix (right).

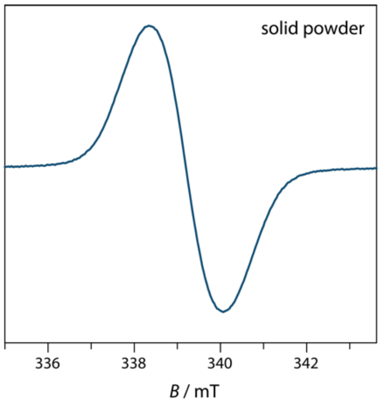

Figure S49: EPR spectrum of insoluble solid from attempted preparation of **DMNC**. Radical impurities cannot be excluded.

Variable-temperature EPR measurements on **DMNC-Ar** at X-band (9.38 GHz) were carried out using a Bruker ELEXSYS E580 CW EPR spectrometer equipped with an Oxford Instruments helium cryostat (ESR900) and a MercuryiTC temperature controller.

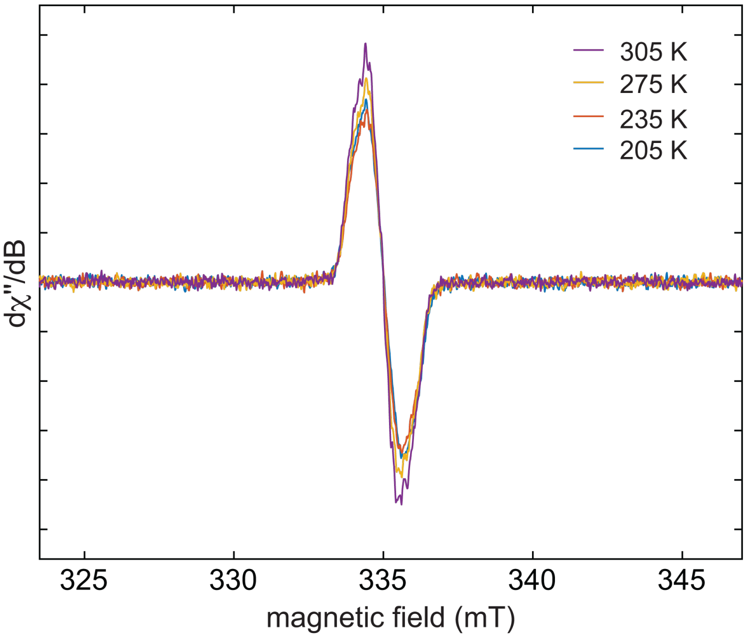

Figure S50: EPR spectra of **DMNC-Ar** in toluene solution at different temperatures. The signal is centered at g = 2.0026 and has a peak-to-peak linewidth (DB_{pp}) of 1.2 mT.

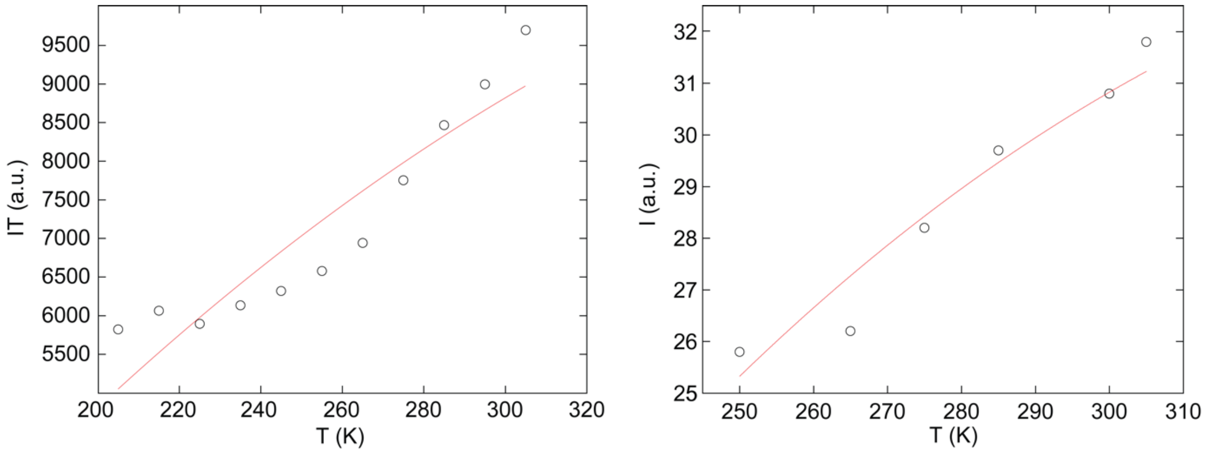

Figure S51: Temperature dependence of the double integral EPR intensity (I or IT) of **DMNC-Ar** in toluene solution of all measured data points (left) and of all points above 230 K (right). Circles (◯) represent the experimental data points and the red lines corresponds to the fit with the Bleaney–Bowers equation.

Fitting of the double-integral intensity of the signal against temperature using the Bleaney–Bowers equation gives a singlet–triplet gap of ΔE_{ST} = 4.16 kJ mol^{-1}(0.994 kcal mol^{-1}) when all data points are included and a singlet–triplet gap of ΔE_{ST} = 5.82 kJ mol^{-1} (1.39 kcal mol^{-1}) when only points above 230 K are considered, both suggesting a singlet ground state. At temperatures below 230 K, a plateau of the signal intensity was observed, which is likely caused by the presence of monoradical impurities whose signal starts to dominate at lower temperatures.

UV–vis–NIR Spectroscopy

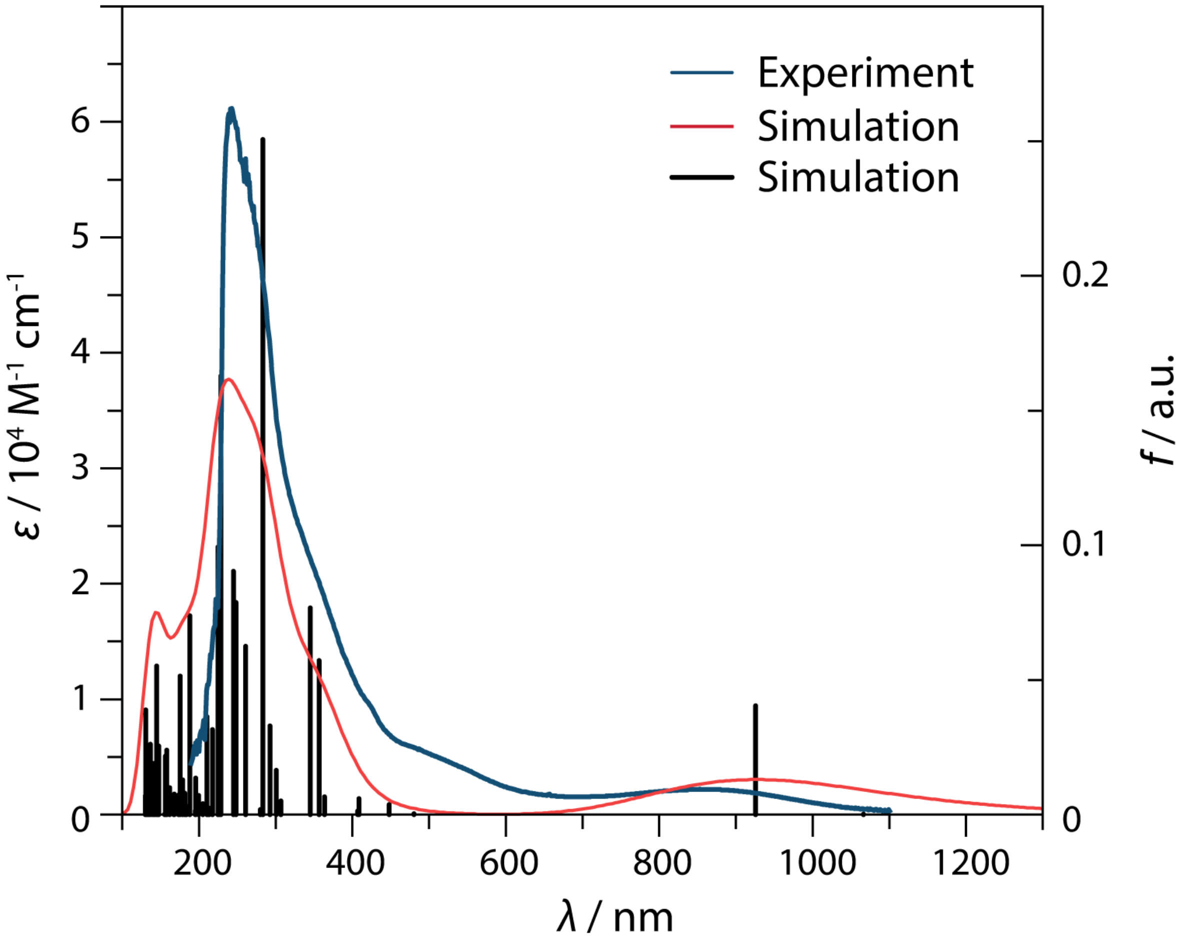

Figure S52: Comparison of the experimental UV–vis–NIR absorption spectrum of **DMNC-Ar** (blue line) in CH_2Cl_2 with the spectrum simulated by TD-DFT calculations (red line and black vertical lines; B3LYP/6-31G(d,p)(CH_2Cl_2)). The calculated spectra are shifted by 0.21 eV. *f* = oscillator strength.

The raw UV–vis data (experimental and calculated) is available free of charge on a public repository Zenodo (DOI:10.5281/zenodo.7733689).

X-Ray Crystallography

Single-crystal X-ray diffraction data were collected at 160(1) K on a Rigaku OD SuperNova/Atlas area-detector diffractometer using Cu K_α radiation (λ = 1.54184 Å) from a micro-focus X-ray source and an Oxford Instruments Cryojet XL cooler. The selected suitable single crystals were mounted using polybutene oil on a flexible loop fixed on a goniometer head and immediately transferred to the diffractometer. Pre-experiments, data collections, data reductions and analytical absorption corrections[4] were performed with the program suite *CrysAlisPro*.[5] Using *Olex2*,[6] the structure was solved with the *SHELXT*[7] small-molecule structure solution program and refined with the *SHELXL2018/3* program package[8] by full-matrix least-squares minimization on F^2. *PLATON*[9] was used to check the results of the X-ray analyses. For more details about the data collection and refinement parameters, see the CIF file.

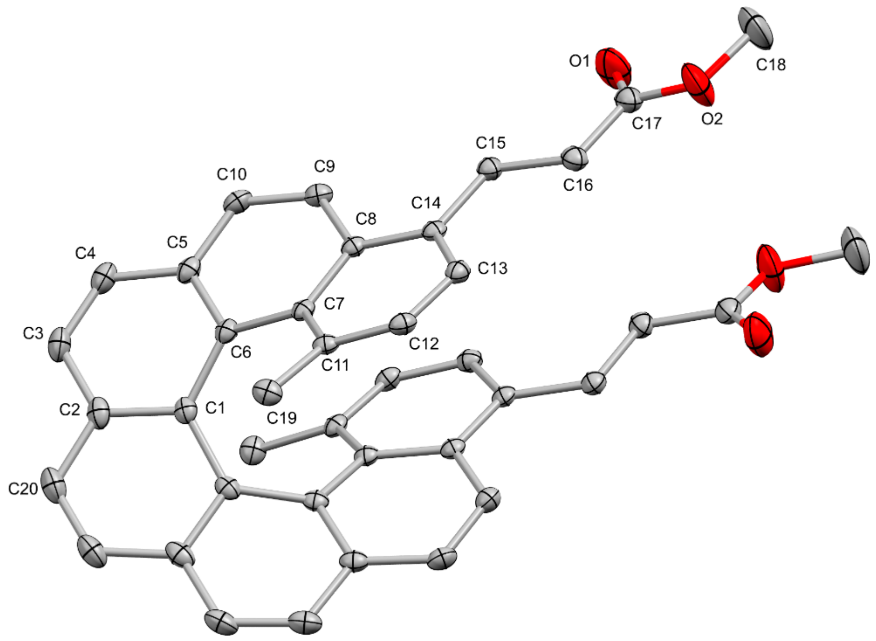

Figure S53: Solid-state structure of **9**. Hydrogen atoms are omitted for clarity.

Yellow crystals were obtained by slow evaporation from $CHCl_3$ at 25 °C for the SC-XRD measurement.

Special features

The molecule lies on a two-fold axis.

Table S1: Crystal data and structure refinement for **9**.

Identification code	**9** (CCDC Number: 2233868)
Empirical formula	$C_{41}H_{36}Cl_2O_4$
Formula weight	663.60
Temperature / K	160(1)
Crystal system	monoclinic
Space group	*I*2/*a*
a / Å	8.02420(10)
b / Å	30.1775(4)
c / Å	13.9999(2)
α / °	90
β / °	105.072(2)
γ / °	90
Volume / Å^3	3273.46(8)
Z	4
ρ_{calc} / g/cm^3	1.346
μ / mm^{-1}	2.127
F(000)	1392.0
Crystal size / mm^3	0.25 × 0.2 × 0.11
Radiation	Cu Kα (λ = 1.54184)
2Θ range for data collection / °	5.858 to 148.892
Index ranges	$-10 \leq h \leq 10$, $-37 \leq k \leq 36$, $-16 \leq l \leq 17$
Reflections collected	16896
Independent reflections	3346 [R_{int} = 0.0167, R_{sigma} = 0.0099]
Data/restraints/parameters	3346/13/228
Goodness-of-fit on F^2	1.078
Final *R* indexes [$I \geq 2\sigma(I)$]	R_1 = 0.0651, wR_2 = 0.1836
Final *R* indexes [all data]	R_1 = 0.0669, wR_2 = 0.1853
Largest diff. peak/hole / e Å^{-3}	1.12/–0.79

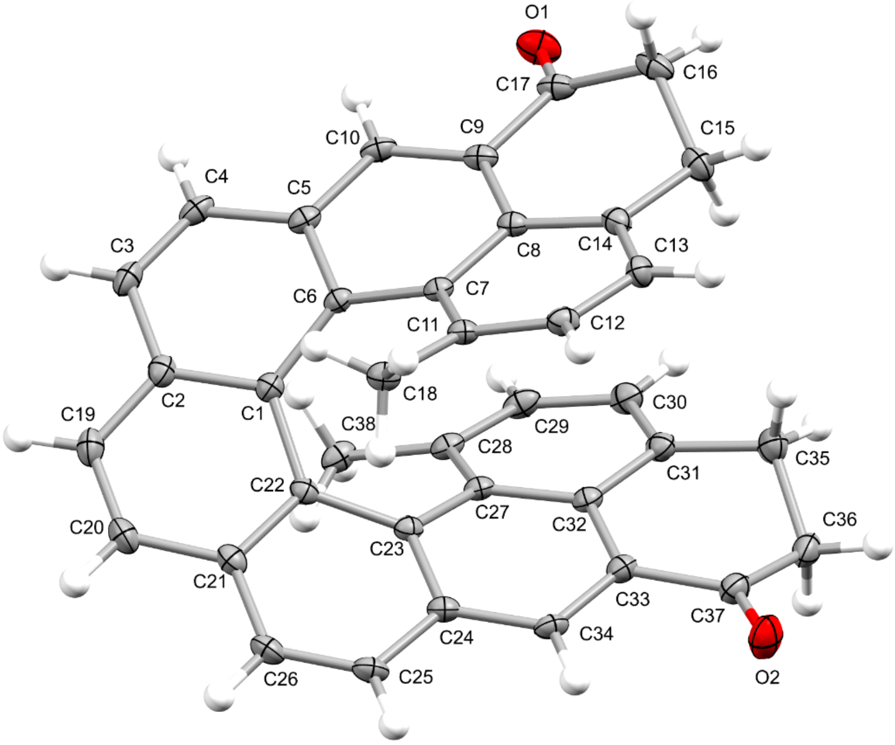

Figure S54: Solid-state structure of **5**.

Yellow crystals were obtained by slow evaporation from $CHCl_3$ at 25 °C for the SC-XRD measurement.

Table S2: Crystal data and structure refinement for **5**.

Identification code	**5** (CCDC Number: 2233869)
Empirical formula	$C_{38}H_{26}O_2$
Formula weight	514.59
Temperature / K	160(1)
Crystal system	triclinic
Space group	*P*-1
a / Å	8.1425(3)
b / Å	12.3731(4)
c / Å	13.4276(5)
α / °	71.820(3)
β / °	73.113(3)
γ / °	74.791(3)
Volume / Å^3	1207.72(8)
Z	2
ρ_{calc} / g/cm^3	1.415
μ / mm^{-1}	0.669
F(000)	540.0
Crystal size / mm^3	0.21 × 0.1 × 0.06
Radiation	Cu Kα (λ = 1.54184)
2Θ range for data collection / °	7.116 to 148.986
Index ranges	$-10 \le h \le 10$, $-15 \le k \le 14$, $-16 \le l \le 16$
Reflections collected	23349
Independent reflections	4924 [R_{int} = 0.0243, R_{sigma} = 0.0200]
Data/restraints/parameters	4924/0/363
Goodness-of-fit on F^2	1.068
Final R indexes [$I \ge 2\sigma(I)$]	R_1 = 0.0427, wR_2 = 0.1190
Final R indexes [all data]	R_1 = 0.0483, wR_2 = 0.1231
Largest diff. peak/hole / e Å^{-3}	0.33/–0.18

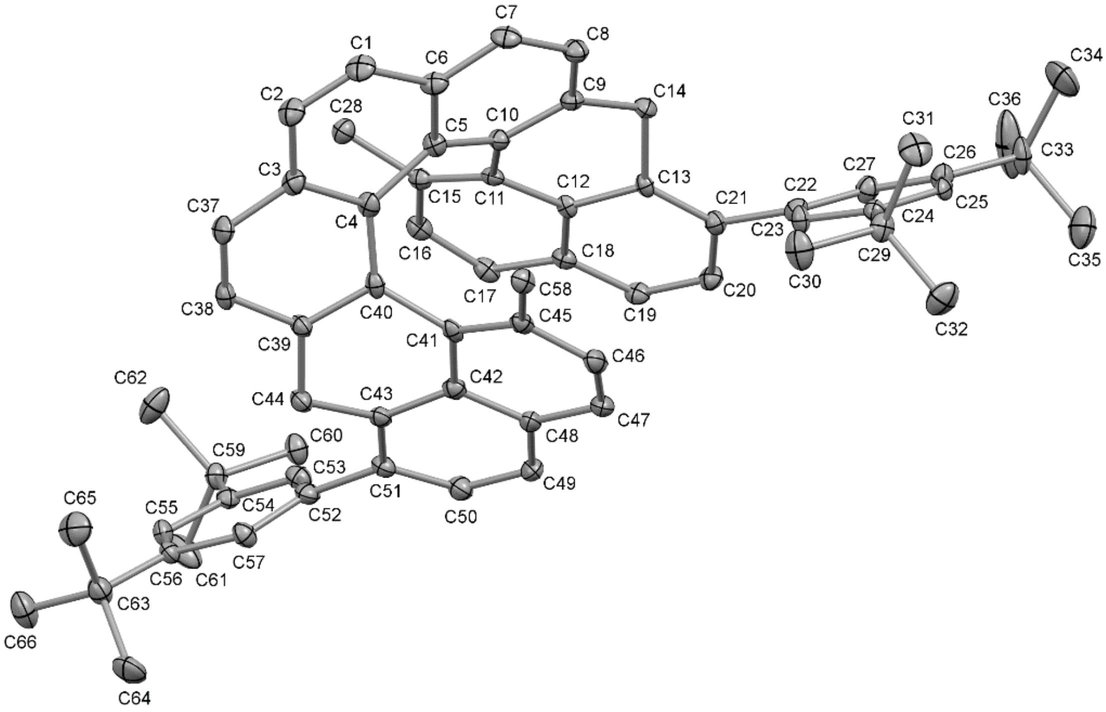

Figure S55: Solid-state structure of 2*H*-**DMNC-Ar**. Hydrogen atoms are omitted for clarity.

Yellow crystals were obtained by slow evaporation from $CHCl_3$ at 25 °C for the SC-XRD measurement.

Table S3: Crystal data and structure refinement for 2*H*-**DMNC-Ar**.

Identification code	2*H*-**DMNC-Ar** (CCDC Number: 2233870)
Empirical formula	$C_{78}H_{78}$
Formula weight	1015.40
Temperature / K	160(1)
Crystal system	monoclinic
Space group	*Pc*
a / Å	18.35075(14)
b / Å	9.98785(8)
c / Å	16.08593(13)
α / °	90
β / °	91.0918(7)
γ / °	90
Volume / Å^3	2947.77(4)
Z	2
ρ_{calc} / g/cm^3	1.144
μ / mm^{-1}	0.479
F(000)	1092.0
Crystal size / mm^3	0.19 × 0.16 × 0.06
2Θ range for data collection / °	4.816 to 148.968°
Index ranges	$-22 \leq h \leq 22$, $-12 \leq k \leq 12$, $-19 \leq l \leq 20$
Reflections collected	54916
Independent reflections	11491[R(int) = 0.0305]
Data/restraints/parameters	11491/2/717
Goodness-of-fit on F^2	1.046
Final R indexes [$I \geq 2\sigma(I)$]	R_1 = 0.0865, wR_2 = 0.2150
Final R indexes [all data]	R_1 = 0.0908, wR_2 = 0.2284
Largest diff. peak/hole / e Å^{-3}	0.98/–0.27
Flack parameter	0.3(4)

Quantum Chemical Calculations

DFT calculations were performed using Gaussian 16 suite.[10] The multireference CASSCF calculations were performed using ORCA 5.0.3.[11] Geometries were optimized using the functionals listed in Tables Table S5–Table S7 and Def2SVP basis set in the gas phase. The frequency analysis was performed to verify the stationary-state geometry, where no imaginary frequency was found. TD-DFT calculations were performed on the ωB97XD/Def2SVP-optimized geometries at the ωB97XD/Def2SVP level of theory. The effect of the solvent was accounted for using CPCM (with CH_2Cl_2 as solvent). Chemcraft software was used to generate graphical images of the frontier molecular orbitals.

The *tert*-butyl substituents were approximated as methyl groups for lower computational cost. For **DMNC-Ar**, the singlet geometries were optimized with spin-restricted and spin-unrestricted broken-symmetry wavefunctions, whereas the triplet geometry was optimized with spin-unrestricted wavefunctions. The B3LYP functional was used for the HOMO–LUMO energy gap calculations because the obtained gap of cethrene was close to the experimental optical energy gap and resulted in a HOMO–LUMO energy gap of 0.94 eV for **DMNC-Ar**. The hyperfine coupling constants were calculated by using unrestricted B3LYP functional EPR-III basis set and solvation effects were taken into account by using CPCM (with benzene as solvent). The isotropic EPR was simulated using WinSim software and the used parameters are shown in Table S8.[12] The diradical character y_0 was calculated via Yamaguchi's formula where the orbital occupations of the HOMO and LUMO from multireference CASSCF(2,2) calculations were used instead of natural orbital occupations from DFT.[13] The adiabatic singlet–triplet energy gap was calculated using the CASSCF(2,2)/NEVPT2/Def2TZVPP approach on DFT-optimized singlet and triplet geometries. The nucleus independent chemical shifts (NICS)[14] were calculated at the B3LYP/6-311+G(2d,p) levels of theory using gauge-independent atomic orbital method (NMR=GIAO keyword in Gaussian). ACID[15,16] plots were calculated at the HF/6-311G(d,p) level of theory for **DMNC-Ar** using the optimized geometry from DFT.

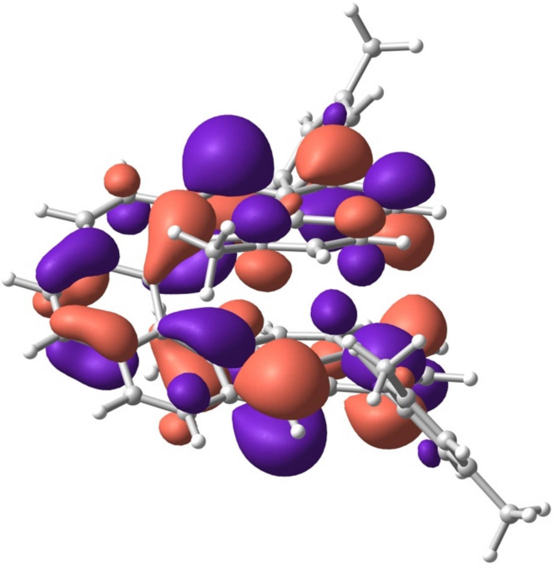

HOMO (1.41)

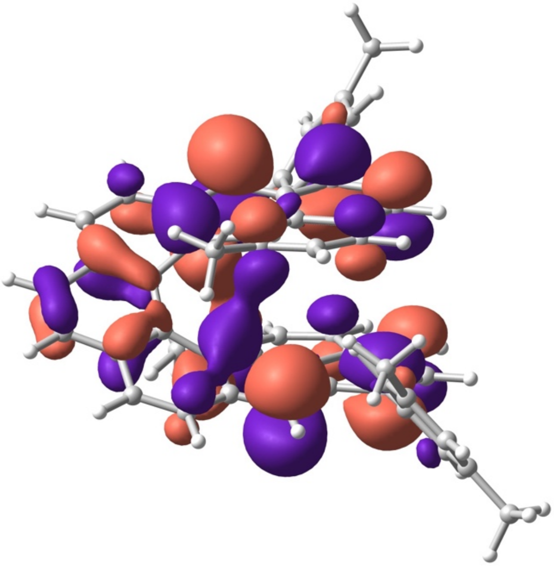

LUMO (0.59)

Figure S56: HOMO (left) and LUMO (right) of **DMNC-Ar** obtained from CAS(2,2)/NEVPT2/Def2TZVPP calculations (occupation numbers shown). Contour value = 0.022.

Table S4: A summary of Mulliken charges, spin densities, and isotropic fermi contact couplings for **DMNC-Ar**.

DMNC-Ar (triplet state)

Mulliken charges and spin densities

		1	2
1	C	-0.139936	0.000986
2	C	-0.140099	0.001002
3	C	0.177315	0.049796
4	C	0.176987	0.049758
5	C	-0.205683	0.028830
6	C	-0.205126	0.028818
7	C	-0.066931	-0.050231
8	C	0.026668	0.181265
9	C	-0.058921	-0.082802
10	C	-0.143748	0.056008
11	C	-0.031455	0.335660
12	C	-0.153829	-0.095961
13	C	0.138029	0.069151
14	C	-0.056513	-0.064473
15	C	-0.138555	-0.090218
16	C	-0.099475	0.171274
17	C	-0.074655	-0.086296
18	C	0.010001	0.178970
19	C	0.026652	0.181276
20	C	-0.058896	-0.082776
21	C	-0.143818	0.056003
22	C	-0.066631	-0.050213
23	C	-0.058638	-0.064548
24	C	0.137831	0.069159
25	C	-0.153270	-0.095965
26	C	-0.030996	0.335679
27	C	-0.139428	-0.090145
28	C	-0.091439	0.271847
29	C	-0.201331	-0.124777
30	C	0.159644	0.256905
31	C	-0.098853	0.171261
32	C	-0.074625	-0.086298
33	C	0.010601	0.178989
34	C	-0.091713	0.271883
35	C	-0.200000	-0.124799
36	C	0.158929	0.256979
37	C	0.305563	-0.026570
38	C	0.303759	-0.026537
39	C	-0.291709	0.031114
40	C	0.076718	-0.009688
41	C	-0.128235	0.027675
42	C	0.063513	-0.012282
43	C	-0.297372	0.025265
44	C	-0.364803	0.001501
45	C	-0.345759	0.001003
46	C	-0.295727	0.025315
47	C	0.061243	-0.012343
48	C	-0.136156	0.027771
49	C	0.081191	-0.009710
50	C	-0.283338	0.031029
51	C	-0.365602	0.001524
52	C	-0.345168	0.000986
53	C	-0.416385	-0.014483
54	C	-0.416856	-0.014492
55	H	0.097930	-0.002697
56	H	0.097906	-0.002697
57	H	0.095110	0.003160
58	H	0.093697	-0.001587

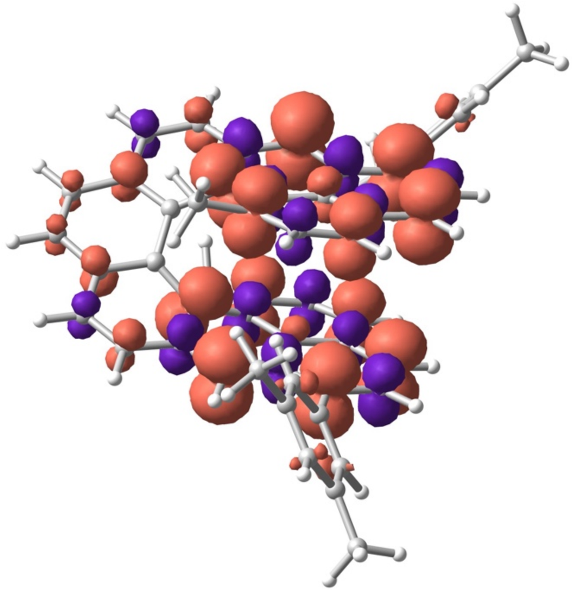

Contour value = 0.003

59	H	0.129919	-0.020595
60	H	0.103246	-0.012367
61	H	0.130072	0.004895
62	H	0.093695	-0.001587
63	H	0.095099	0.003160
64	H	0.129595	-0.020599
65	H	0.104613	-0.016146
66	H	0.091216	0.005477
67	H	0.103418	-0.012367
68	H	0.130148	0.004903
69	H	0.104569	-0.016144
70	H	0.091276	0.005476
71	H	0.108809	-0.001536
72	H	0.084540	-0.001708
73	H	0.137148	-0.002449
74	H	0.122231	-0.000131
75	H	0.127029	-0.000425
76	H	0.113879	-0.000014
77	H	0.121761	-0.000301
78	H	0.107010	-0.000089
79	H	0.128510	-0.000393
80	H	0.137360	-0.002443
81	H	0.084760	-0.001710
82	H	0.108187	-0.001545
83	H	0.114399	-0.000025
84	H	0.121804	-0.000119
85	H	0.127113	-0.000444
86	H	0.106803	-0.000087
87	H	0.128456	-0.000389
88	H	0.121879	-0.000306
89	H	0.135442	0.003839
90	H	0.189172	0.010999
91	H	0.127206	0.002534
92	H	0.135465	0.003843
93	H	0.189277	0.011012
94	H	0.127278	0.002529

Sum of Mulliken charges = -0.00000 2.00000

Isotropic Fermi Contact Couplings

	Atom	a.u.	MegaHertz	Gauss	10(-4) cm-1
1	C(13)	-0.00725	-4.07698	-1.45477	-1.35994
2	C(13)	-0.00725	-4.07696	-1.45476	-1.35993
3	C(13)	0.00613	3.44730	1.23008	1.14989
4	C(13)	0.00613	3.44723	1.23006	1.14987
5	C(13)	-0.00009	-0.05217	-0.01861	-0.01740
6	C(13)	-0.00009	-0.05229	-0.01866	-0.01744
7	C(13)	-0.00559	-3.14169	-1.12103	-1.04795
8	C(13)	0.01714	9.63578	3.43829	3.21415
9	C(13)	-0.02005	-11.26871	-4.02096	-3.75884
10	C(13)	0.00782	4.39688	1.56892	1.46664
11	C(13)	0.02662	14.96165	5.33869	4.99067
12	C(13)	-0.02193	-12.32608	-4.39825	-4.11154
13	C(13)	0.01770	9.94821	3.54977	3.31836
14	C(13)	-0.01693	-9.51476	-3.39510	-3.17378
15	C(13)	-0.01509	-8.47973	-3.02578	-2.82853
16	C(13)	0.01412	7.93688	2.83208	2.64746
17	C(13)	-0.01357	-7.62780	-2.72179	-2.54436
18	C(13)	0.01889	10.61753	3.78860	3.54163
19	C(13)	0.01714	9.63598	3.43836	3.21422
20	C(13)	-0.02005	-11.26876	-4.02097	-3.75885
21	C(13)	0.00782	4.39701	1.56896	1.46669
22	C(13)	-0.00559	-3.14164	-1.12102	-1.04794
23	C(13)	-0.01693	-9.51467	-3.39507	-3.17375
24	C(13)	0.01770	9.94940	3.55019	3.31876
25	C(13)	-0.02193	-12.32617	-4.39828	-4.11157
26	C(13)	0.02662	14.96173	5.33872	4.99070
27	C(13)	-0.01508	-8.47911	-3.02556	-2.82833

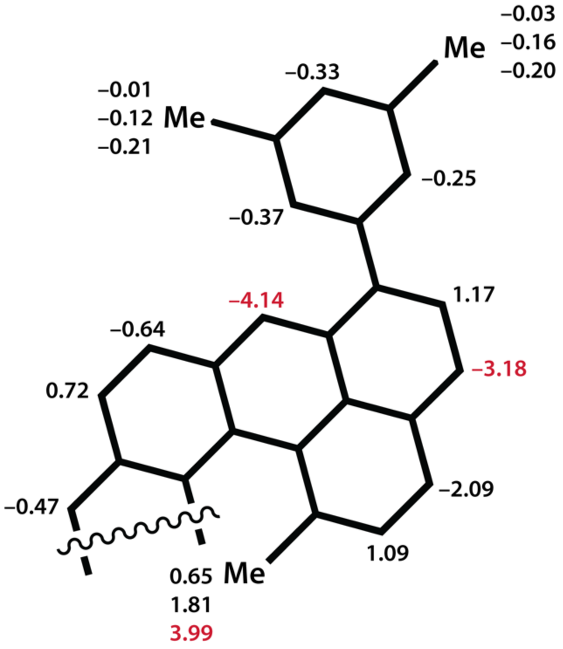

Hyperfine coupling constants of the hydrogen atoms (in Gauss), the largest values were used for the simulation (red)

28	C(13)	0.02057	11.56318	4.12603	3.85706
29	C(13)	-0.01887	-10.60793	-3.78517	-3.53843
30	C(13)	0.02360	13.26560	4.73350	4.42493
31	C(13)	0.01412	7.93642	2.83191	2.64731
32	C(13)	-0.01357	-7.62740	-2.72165	-2.54423
33	C(13)	0.01889	10.61698	3.78840	3.54144
34	C(13)	0.02057	11.56417	4.12638	3.85739
35	C(13)	-0.01887	-10.60803	-3.78521	-3.53846
36	C(13)	0.02360	13.26530	4.73339	4.42483
37	C(13)	-0.00989	-5.56054	-1.98414	-1.85480
38	C(13)	-0.00989	-5.56174	-1.98457	-1.85520
39	C(13)	0.00907	5.09592	1.81835	1.69982
40	C(13)	-0.00113	-0.63612	-0.22698	-0.21219
41	C(13)	0.00212	1.19013	0.42467	0.39699
42	C(13)	-0.00155	-0.87231	-0.31126	-0.29097
43	C(13)	0.00594	3.33661	1.19059	1.11297
44	C(13)	0.00041	0.23074	0.08233	0.07697
45	C(13)	0.00055	0.31006	0.11064	0.10343
46	C(13)	0.00594	3.33886	1.19139	1.11372
47	C(13)	-0.00156	-0.87634	-0.31270	-0.29232
48	C(13)	0.00213	1.19507	0.42643	0.39863
49	C(13)	-0.00114	-0.63852	-0.22784	-0.21299
50	C(13)	0.00905	5.08685	1.81511	1.69679
51	C(13)	0.00041	0.23156	0.08263	0.07724
52	C(13)	0.00055	0.31014	0.11066	0.10345
53	C(13)	-0.00650	-3.65356	-1.30368	-1.21870
54	C(13)	-0.00650	-3.65332	-1.30359	-1.21861
55	H(1)	-0.00059	-1.32552	-0.47298	-0.44214
56	H(1)	-0.00059	-1.32545	-0.47295	-0.44212
57	H(1)	0.00090	2.02094	0.72112	0.67411
58	H(1)	-0.00080	-1.79562	-0.64072	-0.59896
59	H(1)	-0.00519	-11.60382	-4.14053	-3.87062
60	H(1)	-0.00262	-5.85716	-2.08998	-1.95374
61	H(1)	0.00137	3.06002	1.09189	1.02071
62	H(1)	-0.00080	-1.79548	-0.64067	-0.59891
63	H(1)	0.00090	2.02095	0.72112	0.67411
64	H(1)	-0.00519	-11.60355	-4.14044	-3.87053
65	H(1)	-0.00399	-8.91414	-3.18079	-2.97344
66	H(1)	0.00147	3.27836	1.16980	1.09354
67	H(1)	-0.00262	-5.85672	-2.08982	-1.95359
68	H(1)	0.00137	3.05998	1.09188	1.02070
69	H(1)	-0.00399	-8.91509	-3.18112	-2.97375
70	H(1)	0.00147	3.27842	1.16982	1.09356
71	H(1)	-0.00047	-1.05169	-0.37527	-0.35081
72	H(1)	-0.00041	-0.92592	-0.33039	-0.30885
73	H(1)	-0.00032	-0.71052	-0.25353	-0.23700
74	H(1)	-0.00015	-0.34609	-0.12349	-0.11544
75	H(1)	-0.00026	-0.58034	-0.20708	-0.19358
76	H(1)	-0.00001	-0.02167	-0.00773	-0.00723
77	H(1)	-0.00019	-0.42907	-0.15310	-0.14312
78	H(1)	-0.00003	-0.07523	-0.02684	-0.02509
79	H(1)	-0.00025	-0.55444	-0.19784	-0.18494
80	H(1)	-0.00032	-0.71315	-0.25447	-0.23788
81	H(1)	-0.00042	-0.92920	-0.33156	-0.30995
82	H(1)	-0.00047	-1.05071	-0.37492	-0.35048
83	H(1)	-0.00001	-0.03142	-0.01121	-0.01048
84	H(1)	-0.00015	-0.32686	-0.11663	-0.10903
85	H(1)	-0.00027	-0.59593	-0.21264	-0.19878
86	H(1)	-0.00003	-0.07284	-0.02599	-0.02430
87	H(1)	-0.00025	-0.55062	-0.19648	-0.18367
88	H(1)	-0.00020	-0.43666	-0.15581	-0.14565
89	H(1)	0.00227	5.06977	1.80902	1.69109
90	H(1)	0.00501	11.18971	3.99277	3.73248
91	H(1)	0.00081	1.82042	0.64957	0.60723
92	H(1)	0.00227	5.07134	1.80958	1.69162
93	H(1)	0.00501	11.18826	3.99225	3.73200
94	H(1)	0.00081	1.81879	0.64899	0.60668

For a better overview, three different functionals, some of which include long- and short-range London dispersive forces, were screened (Table S5 and Table S6). The energies of the open (o) and electrocyclized or closed (c) forms of cethrenes are summarized in Table S7. The S–T gaps and Δ*E* (open–closed) were calculated as triplet energy minus singlet energy and energy of closed minus energy of open, respectively.

Table S5: Energies, energy gaps, and orbital occupation numbers, and diradical characters for **DMNC-Ar** obtained from DFT and CASSCF calculations.

	Energies from DFT [kcal/mol][a]			
functional	c-**DMNC-Ar**	o-**DMNC-Ar** (singlet)	o-**DMNC-Ar** (triplet)	Δ*E* (open–closed)
B3LYP-D3	−1305073.891	−1305086.016	−1305084.321	**12.125**
PBE0	−1303481.808	−1303486.462	−1303485.019	**4.654**
ωB97DX	−1304592.318	−1304598.561	−1304596.684	**6.243**

[a]Using Def2SVP basis set including zero-point vibrational energy correction.

	Energies from CASSCF/NEVPT2 [kcal/mol][b]		orbital occupation[b,c]			S–T gap [kcal/mol]	
functional	o-**DMNC-Ar** (singlet)	o-**DMNC-Ar** (triplet)	HOMO	LUMO	y_0	DFT[a]	CASSCF[b]
B3LYP-D3	−1304298.644	−1304296.691	1.49	0.51	0.56	1.695	**1.953**
PBE0	−1304294.107	−1304293.819	1.33	0.67	0.63	1.443	**0.288**
ωB97DX	−1304299.379	−1304298.474	1.41	0.59	0.59	1.878	**0.905**

[a]Using Def2SVP basis set including zero-point vibrational energy correction. [b]Using Def2-TZVPP basis set. [c]From CASSCF.

Table S6: Energies, energy gaps, and orbital occupation numbers, and diradical characters for **NC** obtained from DFT and CASSCF calculations.

	Energies from DFT [kcal/mol][a]	
functional	o-**NC** (singlet)	o-**NC** (triplet)
B3LYP-D3	−867569.7144	−867568.9231
PBE0	−866522.3588	−866521.3856
ωB97DX[b]	−867239.5772	−867238.0373

[a]Using Def2SVP basis set including zero-point vibrational energy correction. [b]Values taken from reference[17].

	Energies from CASSCF/NEVPT2 [kcal/mol][c]		orbital occupation[c,d]			S–T gap [kcal/mol]	
functional	o-**NC** (singlet)	o-**NC** (triplet)	HOMO	LUMO	y_0	DFT[a]	CASSCF[c]
B3LYP-D3	−867021.4074	−867021.4894	1.09	0.91	0.85	0.791	**−0.082**
PBE0	−867018.299	−867018.3574	1.06	0.94	0.89	0.973	**−0.058**
ωB97DX[b]	n.a	n.a	1.1	0.9	0.83	1.540	**0.17**

[a]Using Def2SVP basis set including zero-point vibrational energy correction. [b]Values taken from reference[17]. [c]Using Def2-TZVPP basis set. [d]From CASSCF.

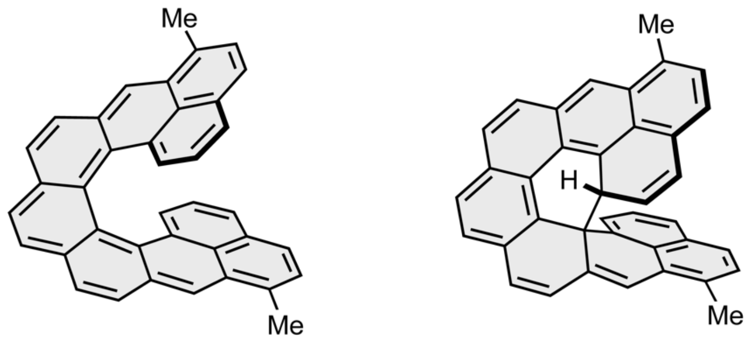

Figure S57: Structures of the open (left) and closed (right) form of **DMNC***.

Table S7: Energies for different cethrenes obtained from DFT calculations. Vertical comparisons indicate the strain which is induced by the methyl groups in the fjord region.

	Energies from DFT [kcal/mol][a]		
functional	closed form	open form	Δ*E* (open–closed)
Cethrene	–674692.1609	–674677.4426	**14.72**
Dimethylcethrene (**DMC**)	–723951.3175	–723934.669	**16.65**
Nonacethrene (**NC**)	–867240.1658	–867239.5772	**0.589**
Dimethylnonacethrene (**DMNC**)	–916492.1807	–916497.2698	**–5.09**
Dimethylnonacethrene*(**DMNC***)[b]	–916501.3932	–916501.3838	**0.00941**

[a]Using ωB97DX functional and Def2SVP basis set including zero-point vibrational energy correction. [b]Methyl groups at different positions (see Figure S57).

Table S8: Summary of used parameters for the EPR simulation using WinSim.[12]

parameter	value
Field center	338.25 mT
Mod. Amp.	0.1 mT
g-shift	2.0055
Lineshape	0
Simple LW	2.8
HCC-1[a]	2x 5.382
HCC-2[a]	2x 5.187
HCC-3[a]	2x 4.137

[a]The values of the hyperfine coupling constants (HCC) from calculations (Table S4) were scaled by 130%.

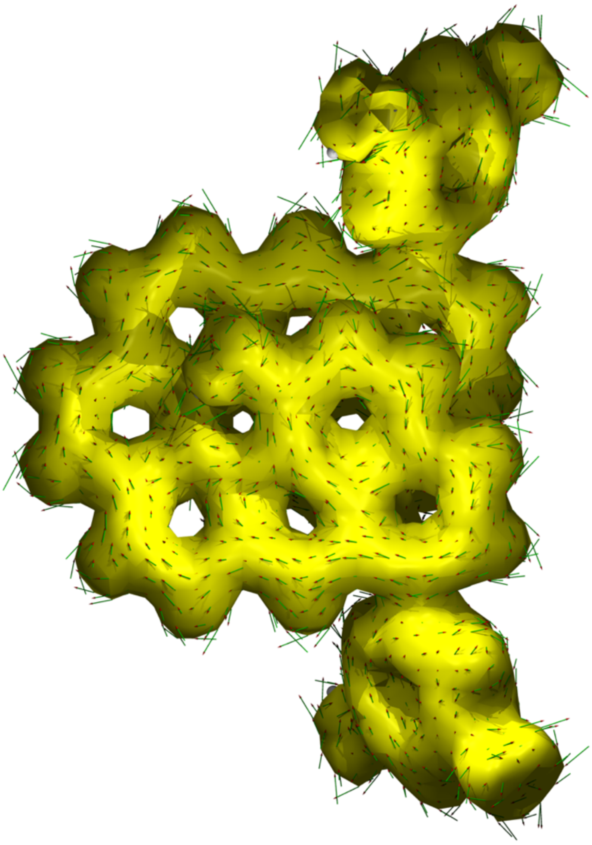

Figure S58: Plot of anisotropy of induced current density (ACID) for **DMNC-Ar**. The current-density vectors plotted onto the ACID isosurface indicate a diatropic ring current around the whole periphery of the backbone and within the individual benzene rings. No paratropic ring current could be found, therefore, no anti-aromatic character could be attributed to **DMNC-Ar**, which is further supported by calculations of the nucleus independent chemical shift (see Table S9 below).

Table S9: Out-of-plane ($NICS_{ZZ}$) nucleus independent chemical shifts for **DMNC-Ar**. The dummy atoms placed above the rings (red dots) indicate that all rings are aromatic even though ring **C** displays much weaker aromaticity. If the dummy atoms are placed below (blue dots), this trend is not observable anymore because of the intramolecular through-space shielding. The NICS values for red dummy atoms indicate a high diradical character because in the diradical structure the main contributing resonance structure is one with two naphthalene (rings **A** and **B**) and one phenanthrene (rings **D** and **E**) aromatic units and unpaired electrons localized in rings **C**.

	$NICS(1)_{ZZ}$ (above, red dots)	$NICS(1)_{ZZ}$ (below, blue dots)
**A**	–20.26	–23.81
**B**	–23.81	–35.12
**C**	–6.83	–25.50
**D**	–27.13	–19.09
**E**	–35.12	–22.85

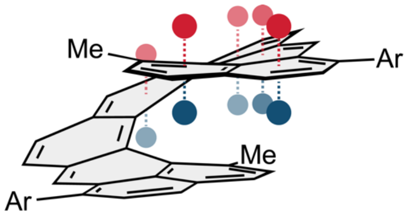

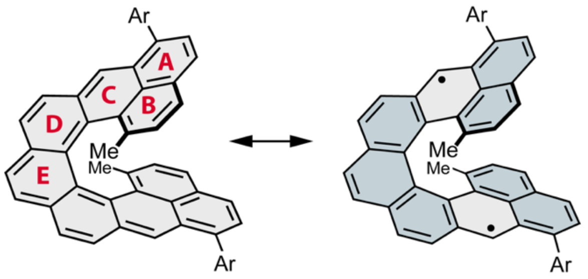

Cartesian Coordinates

The cartesian coordinates are shown for geometries optimized at the ωB97DX/Def2SVP level of DFT in the gas phase. The unrestricted broken-symmetry approach was utilized to adjust for the diradicaloid nature of the open forms of cethrenes, which resulted in the lowest-energy configuration.

Table S10: Cartesian coordinates.

c-DMNC-Ar

Thermochemistry

Temperature: 298.150 Kelvin; Pressure: 1.00000 Atm.
Zero-point correction = 0.775427 (Hartree/Particle)
Thermal correction to Energy = 0.818114
Thermal correction to Enthalpy = 0.819058
Thermal correction to Gibbs Free Energy = 0.696861
Sum of electronic and zero-point Energies= −2078.999750
Sum of electronic and thermal Energies = −2078.957063
Sum of electronic and thermal Enthalpies = −2078.956119
Sum of electronic and thermal Free Energies = −2079.078316
Charge = 0 Multiplicity = 1

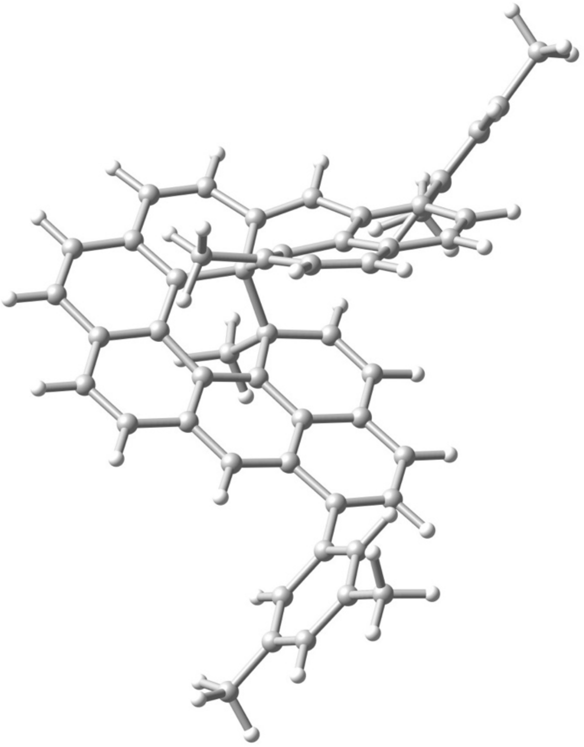

C	-0.861833000	3.114746000	-0.099807000
C	0.541910000	3.101281000	0.022966000
C	-1.585485000	4.314650000	0.058971000
C	1.228946000	4.316505000	0.023859000
C	0.502848000	5.525977000	0.104457000
C	-0.873652000	5.525295000	0.175463000
C	-3.024373000	4.253184000	0.166399000
C	-1.576996000	1.875854000	-0.339048000
C	-2.987710000	1.834326000	-0.158191000
C	-3.685106000	3.070836000	0.116191000
C	-3.656420000	0.622964000	-0.290923000
C	-2.969279000	-0.556095000	-0.626846000
C	-1.560221000	-0.485327000	-0.880975000
C	-0.881635000	0.746182000	-0.766252000
C	-0.838054000	-1.681535000	-1.214388000
C	0.587226000	-1.573273000	-1.498291000
C	1.242079000	-0.410382000	-1.426321000
C	0.593682000	0.911118000	-1.118638000
C	1.258138000	1.750621000	0.092606000
C	2.770298000	1.890778000	-0.049572000
C	3.377553000	3.192200000	-0.271694000
C	2.676769000	4.333206000	-0.156742000
C	1.001460000	0.925424000	1.384395000
C	1.765126000	-0.292606000	1.454071000
C	3.022220000	-0.405263000	0.767483000
C	3.557949000	0.820653000	0.197095000
C	1.337910000	-1.369524000	2.267636000
C	2.061752000	-2.588871000	2.243165000
C	3.221640000	-2.701994000	1.520254000
C	3.743293000	-1.596851000	0.801284000
C	0.216661000	-1.177507000	3.112446000
C	-0.340809000	0.065165000	3.207933000
C	0.055570000	1.157355000	2.379340000
C	-1.502912000	-2.882861000	-1.256343000
C	-2.895165000	-2.954358000	-1.008660000
C	-3.634979000	-1.833308000	-0.719274000
C	-5.103236000	-1.959015000	-0.501544000
C	5.050330000	-1.751151000	0.107802000
C	6.152002000	-2.276905000	0.795636000
C	7.387362000	-2.446184000	0.167662000
C	7.505552000	-2.089130000	-1.180069000
C	6.425372000	-1.571570000	-1.898833000
C	5.204120000	-1.406299000	-1.239232000
C	-5.595324000	-2.744616000	0.541336000
C	-6.972508000	-2.899554000	0.746981000
C	-7.851142000	-2.249742000	-0.119222000
C	-7.389201000	-1.458251000	-1.179861000
C	-6.013353000	-1.321100000	-1.357460000
C	-8.366225000	-0.796017000	-2.116995000
C	-7.476995000	-3.742119000	1.890133000
C	6.556179000	-1.232480000	-3.361360000
C	8.578757000	-2.965291000	0.930724000
C	0.729274000	1.709242000	-2.443671000
C	-0.585746000	2.477821000	2.746108000
H	1.053312000	6.469800000	0.124523000
H	-1.422808000	6.462002000	0.297228000
H	-3.570903000	5.185439000	0.329079000
H	-4.769824000	3.035353000	0.243080000
H	-4.734919000	0.591490000	-0.129010000
H	1.120397000	-2.482614000	-1.786389000

H	2.292943000	-0.369325000	-1.709875000
H	4.457801000	3.218922000	-0.435923000
H	3.173153000	5.302895000	-0.240558000
H	4.641234000	0.908932000	0.085487000
H	1.688785000	-3.434710000	2.825919000
H	3.769400000	-3.646792000	1.505016000
H	-0.145076000	-2.005019000	3.726955000
H	-1.138701000	0.243870000	3.933584000
H	-0.951461000	-3.795229000	-1.496045000
H	-3.400700000	-3.920877000	-1.072306000
H	6.043843000	-2.540138000	1.851538000
H	8.468520000	-2.219604000	-1.683778000
H	4.348229000	-1.012187000	-1.792319000
H	-4.887739000	-3.236611000	1.214758000
H	-8.929599000	-2.360646000	0.031502000
H	-5.631270000	-0.714379000	-2.183128000
H	-9.168364000	-0.286240000	-1.562339000
H	-8.845571000	-1.539300000	-2.774016000
H	-7.870413000	-0.054447000	-2.758749000
H	-7.072665000	-4.764688000	1.837134000
H	-8.573701000	-3.811055000	1.888697000
H	-7.166393000	-3.319628000	2.858548000
H	7.596912000	-1.002767000	-3.630638000
H	5.933480000	-0.366812000	-3.629890000
H	6.230908000	-2.077827000	-3.989236000
H	8.271280000	-3.548538000	1.810141000
H	9.206508000	-2.133112000	1.288564000
H	9.212087000	-3.605286000	0.299236000
H	0.202181000	2.670921000	-2.416336000
H	0.313301000	1.113660000	-3.267966000
H	1.791756000	1.902541000	-2.658739000
H	-1.613607000	2.565698000	2.362755000
H	-0.016397000	3.348151000	2.407007000
H	-0.647623000	2.530924000	3.843383000

o-**DMNC-Ar** (broken-symmetry unrestricted singlet)

C	-0.652226000	3.203486000	-0.313170000
C	0.652218000	3.203489000	0.313156000
C	-1.370634000	4.425260000	-0.356785000
C	1.370613000	4.425270000	0.356779000
C	0.673809000	5.645117000	0.127604000
C	-0.673837000	5.645112000	-0.127618000
C	-2.775903000	4.392496000	-0.624350000
C	-1.261427000	2.029086000	-0.866184000
C	-2.679901000	1.975997000	-0.892910000
C	-3.420082000	3.201747000	-0.789530000
C	-3.321339000	0.725767000	-0.950784000
C	-2.583679000	-0.478083000	-0.990427000
C	-1.181572000	-0.395819000	-1.304306000
C	-0.531077000	0.873751000	-1.371996000
C	-0.456766000	-1.586502000	-1.587401000
C	0.878423000	-1.481463000	-2.032807000
C	1.426154000	-0.239778000	-2.275505000
C	0.744997000	0.954592000	-1.982235000
C	1.261414000	2.029096000	0.866194000
C	2.679887000	1.976023000	0.892957000
C	3.420061000	3.201778000	0.789583000
C	2.775875000	4.392519000	0.624375000
C	0.531069000	0.873753000	1.372002000
C	1.181578000	-0.395810000	1.304318000
C	2.583689000	-0.478059000	0.990447000
C	3.321337000	0.725799000	0.950838000
C	0.456782000	-1.586503000	1.587404000
C	1.099154000	-2.842090000	1.430763000
C	2.421289000	-2.913849000	1.039175000
C	3.186781000	-1.756316000	0.817592000
C	-0.878416000	-1.481482000	2.032787000
C	-1.426170000	-0.239805000	2.275469000
C	-0.745026000	0.954573000	1.982210000
C	-1.099120000	-2.842097000	-1.430748000
C	-2.421254000	-2.913872000	-1.039153000

Thermochemistry

Temperature: 298.150 Kelvin; Pressure: 1.00000 Atm.

Zero-point correction = 0.771642 (Hartree/Particle)

Thermal correction to Energy = 0.814689

Thermal correction to Enthalpy = 0.815634

Thermal correction to Gibbs Free Energy = 0.694382

Sum of electronic and zero-point Energies= −2079.009699

Sum of electronic and thermal Energies = −2078.966652

Sum of electronic and thermal Enthalpies = −2078.965707

Sum of electronic and thermal Free Energies = −2079.086959

Charge = 0 Multiplicity = 1

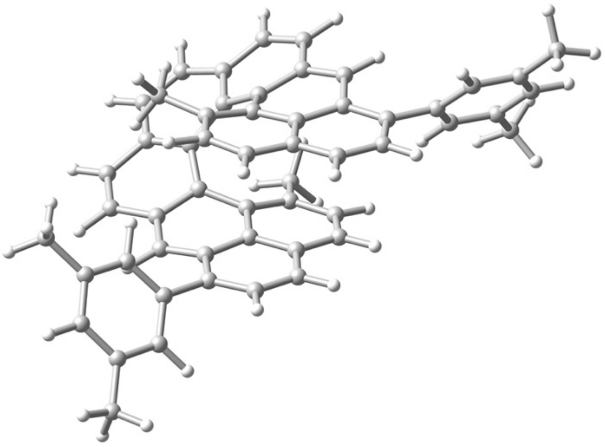

C	-3.186756000	-1.756346000	-0.817575000
C	-4.597273000	-1.899459000	-0.376706000
C	4.597293000	-1.899426000	0.376714000
C	-5.481133000	-2.729765000	-1.071323000
C	-6.803571000	-2.902570000	-0.645841000
C	-7.227029000	-2.231798000	0.502262000
C	-6.364815000	-1.398813000	1.227358000
C	-5.055993000	-1.241071000	0.774959000
C	-7.748060000	-3.772360000	-1.434994000
C	-6.842521000	-0.712867000	2.481380000
C	5.055997000	-1.241083000	-0.775001000
C	6.364804000	-1.398830000	-1.227403000
C	7.227035000	-2.231810000	-0.502297000
C	6.803596000	-2.902543000	0.645819000
C	5.481159000	-2.729706000	1.071330000
C	7.748003000	-3.772462000	1.434927000
C	6.842620000	-0.712761000	-2.481316000
C	1.393026000	2.241699000	-2.426307000
C	-1.393084000	2.241668000	2.426267000
H	1.222125000	6.586723000	0.208984000
H	-1.222162000	6.586714000	-0.208991000
H	-3.332391000	5.332831000	-0.628145000
H	-4.507996000	3.163041000	-0.883262000
H	-4.411349000	0.691693000	-0.912649000
H	1.451216000	-2.389753000	-2.232254000
H	2.421788000	-0.172079000	-2.722382000
H	4.507973000	3.163082000	0.883336000
H	3.332356000	5.332859000	0.628174000
H	4.411348000	0.691740000	0.912737000
H	0.526084000	-3.753752000	1.614084000
H	2.890821000	-3.888777000	0.888386000
H	-1.451202000	-2.389780000	2.232218000
H	-2.421810000	-0.172117000	2.722332000
H	-0.526043000	-3.753752000	-1.614084000
H	-2.890780000	-3.888804000	-0.888370000
H	-5.134737000	-3.239678000	-1.974831000
H	-8.258060000	-2.360467000	0.846537000
H	-4.363182000	-0.604460000	1.330828000
H	-7.270599000	-4.720315000	-1.724438000
H	-8.061431000	-3.268400000	-2.363363000
H	-8.655222000	-4.008220000	-0.861233000
H	-7.808150000	-0.210860000	2.318396000
H	-6.121506000	0.040574000	2.828195000
H	-6.986454000	-1.439348000	3.297022000
H	4.363171000	-0.604515000	-1.330901000
H	8.258056000	-2.360500000	-0.846598000
H	5.134787000	-3.239595000	1.974862000
H	8.655975000	-4.006854000	0.861851000
H	7.271042000	-4.721189000	1.722685000
H	8.059920000	-3.269388000	2.364261000
H	6.120423000	0.038864000	-2.829599000
H	6.989263000	-1.439496000	-3.296237000
H	7.806926000	-0.208497000	-2.317401000
H	1.867707000	2.086303000	-3.406720000
H	2.181170000	2.574765000	-1.733996000
H	0.671054000	3.061586000	-2.524942000
H	-1.867806000	2.086253000	3.406659000
H	-2.181206000	2.574743000	1.733932000
H	-0.671120000	3.061555000	2.524944000

o-**DMNC-Ar** (unrestricted triplet)

C	-0.657192000	3.192937000	-0.303134000
C	0.657069000	3.192980000	0.303010000
C	-1.370731000	4.412055000	-0.342319000
C	1.370573000	4.412121000	0.342100000
C	0.672191000	5.637192000	0.120028000
C	-0.672389000	5.637163000	-0.120326000
C	-2.779706000	4.387995000	-0.592305000
C	-1.285563000	2.013522000	-0.840974000
C	-2.701048000	1.973069000	-0.855310000
C	-3.433210000	3.202069000	-0.744634000
C	-3.359553000	0.728212000	-0.922360000
C	-2.636887000	-0.479037000	-0.973527000
C	-1.233550000	-0.408760000	-1.288304000
C	-0.570811000	0.853892000	-1.354143000
C	-0.521268000	-1.606527000	-1.576064000
C	0.815918000	-1.514417000	-2.017020000
C	1.377079000	-0.277442000	-2.257434000
C	0.705810000	0.922761000	-1.968976000
C	1.285482000	2.013623000	0.840934000
C	2.700969000	1.973208000	0.855248000
C	3.433092000	3.202222000	0.744476000
C	2.779551000	4.388119000	0.592072000
C	0.570777000	0.854016000	1.354219000
C	1.233545000	-0.408626000	1.288461000
C	2.636876000	-0.478895000	0.973650000
C	3.359505000	0.728372000	0.922385000
C	0.521293000	-1.606383000	1.576332000
C	1.177995000	-2.855122000	1.426823000
C	2.503487000	-2.916284000	1.039808000
C	3.256314000	-1.754320000	0.811685000
C	-0.815898000	-1.514269000	2.017272000
C	-1.377095000	-0.277290000	2.257582000
C	-0.705837000	0.922903000	1.969060000
C	-1.177937000	-2.855268000	-1.426454000
C	-2.503441000	-2.916426000	-1.039464000
C	-3.256298000	-1.754462000	-0.811480000
C	-4.667326000	-1.883833000	-0.368999000
C	4.667300000	-1.883704000	0.369099000
C	-5.562765000	-2.696407000	-1.069819000
C	-6.886366000	-2.857258000	-0.643279000
C	-7.299359000	-2.192571000	0.512122000
C	-6.425466000	-1.377612000	1.243760000
C	-5.115722000	-1.231275000	0.790191000
C	-7.842468000	-3.708426000	-1.438730000
C	-6.891311000	-0.700114000	2.506812000
C	5.115640000	-1.231146000	-0.790018000
C	6.425290000	-1.377916000	-1.243955000
C	7.298992000	-2.193280000	-0.512726000
C	6.886089000	-2.857852000	0.642893000
C	5.562697000	-2.696710000	1.069665000
C	7.843513000	-3.707161000	1.438735000
C	6.891350000	-0.700051000	-2.506742000
C	1.352747000	2.203193000	-2.435870000
C	-1.352793000	2.203352000	2.435885000
H	1.223860000	6.577162000	0.196870000
H	-1.224086000	6.577109000	-0.197238000
H	-3.329444000	5.332258000	-0.591297000
H	-4.522306000	3.169491000	-0.825702000
H	-4.449719000	0.707720000	-0.881390000
H	1.379956000	-2.428174000	-2.216373000
H	2.373833000	-0.219849000	-2.703345000
H	4.522190000	3.169684000	0.825530000
H	3.329262000	5.332397000	0.590990000
H	4.449670000	0.707905000	0.881398000
H	0.613605000	-3.772089000	1.611019000
H	2.982218000	-3.887792000	0.895898000
H	-1.379913000	-2.428027000	2.216690000
H	-2.373861000	-0.219682000	2.703466000
H	-0.613518000	-3.772237000	-1.610554000
H	-2.982162000	-3.887929000	-0.895487000
H	-5.224352000	-3.201329000	-1.979141000

Thermochemistry

Temperature: 298.150 Kelvin; Pressure: 1.00000 Atm.

Zero-point correction = 0.771812 (Hartree/Particle)

Thermal correction to Energy = 0.814918

Thermal correction to Enthalpy = 0.815862

Thermal correction to Gibbs Free Energy = 0.693540

Sum of electronic and zero-point Energies= –2079.006707

Sum of electronic and thermal Energies = –2078.963601

Sum of electronic and thermal Enthalpies = –2078.962657

Sum of electronic and thermal Free Energies = –2079.084979

Charge = 0 Multiplicity = 3

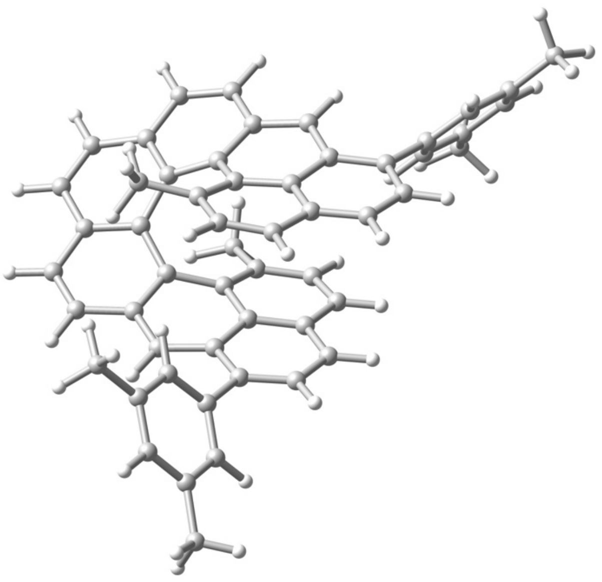

H	-8.331349000	-2.311553000	0.856959000
H	-4.414375000	-0.608460000	1.351207000
H	-7.381814000	-4.665915000	-1.724203000
H	-8.139029000	-3.199099000	-2.369645000
H	-8.758090000	-3.926015000	-0.871258000
H	-7.864628000	-0.208141000	2.360398000
H	-6.172917000	0.059700000	2.845101000
H	-7.014607000	-1.430571000	3.322388000
H	4.414323000	-0.608251000	-1.350973000
H	8.330752000	-2.312920000	-0.858074000
H	5.224192000	-3.201866000	1.978807000
H	8.743349000	-3.954027000	0.858057000
H	7.372693000	-4.649155000	1.756838000
H	8.168620000	-3.180854000	2.350580000
H	6.168422000	0.053051000	-2.850297000
H	7.024533000	-1.431577000	-3.319761000
H	7.859919000	-0.199686000	-2.357059000
H	1.806074000	2.036272000	-3.424512000
H	2.156524000	2.539107000	-1.763396000
H	0.632811000	3.025344000	-2.528703000
H	-1.805979000	2.036520000	3.424608000
H	-2.156673000	2.539104000	1.763451000
H	-0.632917000	3.025573000	2.528516000

o-cethrene (broken-symmetry unrestricted singlet)

C	2.781032000	2.124357000	0.373237000
C	1.406355000	2.112128000	0.148764000
C	0.711190000	0.867787000	-0.045754000
C	1.535381000	-0.295644000	-0.386425000
C	2.937317000	-0.264975000	-0.118540000
C	3.564071000	0.942241000	0.331370000
C	0.667644000	3.346733000	0.112391000
C	-0.667639000	3.346734000	-0.112385000
C	-1.406351000	2.112129000	-0.148757000
C	-0.711188000	0.867788000	0.045765000
C	-2.781028000	2.124358000	-0.373238000
C	-3.564067000	0.942244000	-0.331375000
C	-2.937316000	-0.264973000	0.118539000
C	-1.535382000	-0.295644000	0.386431000
C	-3.743121000	-1.418390000	0.356658000
C	-3.151670000	-2.559537000	0.947180000
C	-1.822987000	-2.531873000	1.330149000
C	-1.026527000	-1.415067000	1.062175000
C	-4.938095000	0.940163000	-0.624526000
C	-5.703765000	-0.220558000	-0.464580000
C	-5.126931000	-1.376476000	0.028323000
C	3.743119000	-1.418393000	-0.356662000
C	3.151664000	-2.559540000	-0.947180000
C	1.822978000	-2.531877000	-1.330139000
C	1.026520000	-1.415069000	-1.062162000
H	-0.015677000	-1.423802000	-1.379029000
H	1.383279000	-3.389392000	-1.843695000
H	-3.766975000	-3.443023000	1.132900000
H	-1.383295000	-3.389387000	1.843712000
H	0.015666000	-1.423799000	1.379054000
H	-3.274262000	3.076447000	-0.585827000
H	-5.409101000	1.864998000	-0.965853000
H	-6.768631000	-0.203216000	-0.706197000
H	-5.732300000	-2.271519000	0.189618000
H	3.766968000	-3.443027000	-1.132900000
C	5.126930000	-1.376479000	-0.028336000
H	1.218209000	4.284223000	0.219110000
H	-1.218204000	4.284224000	-0.219107000
C	4.938100000	0.940160000	0.624513000
H	3.274269000	3.076446000	0.585821000
C	5.703769000	-0.220562000	0.464564000
H	5.409110000	1.864994000	0.965837000
H	6.768636000	-0.203220000	0.706175000
H	5.732297000	-2.271523000	-0.189633000

Thermochemistry

Temperature: 298.150 Kelvin; Pressure: 1.00000 Atm.
Zero-point correction = 0.349000 (Hartree/Particle)
Thermal correction to Energy = 0.366964
Thermal correction to Enthalpy = 0.367908
Thermal correction to Gibbs Free Energy = 0.304020
Sum of electronic and zero-point Energies= −1075.166713
Sum of electronic and thermal Energies = −1075.148749
Sum of electronic and thermal Enthalpies = −1075.147804
Sum of electronic and thermal Free Energies = −1075.211693
Charge = 0 Multiplicity = 1

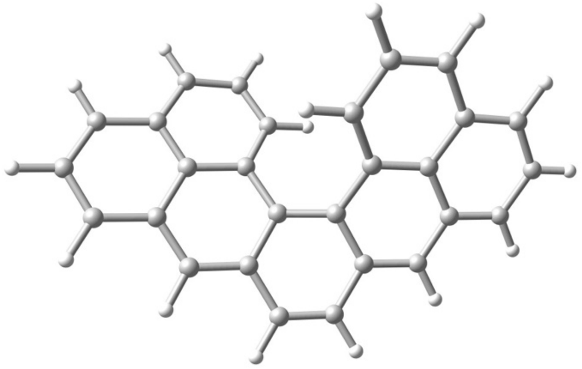

c-cethrene

C	0.003928000	5.655769000	-0.206982000
C	-0.084403000	4.951909000	-1.434604000
C	-0.137911000	3.576208000	-1.459450000
C	-0.102077000	2.840989000	-0.227460000
C	0.011732000	3.549523000	1.005872000
C	0.054260000	4.975927000	0.981142000
C	0.059332000	2.820708000	2.209879000
C	0.047192000	1.434547000	2.211249000
C	-0.011732000	0.729189000	0.969847000
C	-0.145244000	1.427576000	-0.227600000
C	0.011732000	-0.729189000	0.969847000
C	-0.047192000	-1.434547000	2.211249000
C	-0.038495000	-0.673917000	3.446941000
C	0.038495000	0.673917000	3.446941000
C	0.145244000	-1.427576000	-0.227600000
C	0.418968000	-0.647333000	-1.499933000
C	-0.418968000	0.647333000	-1.499933000
C	-0.291753000	1.487959000	-2.739050000
C	-0.192637000	2.822150000	-2.710915000
C	0.102077000	-2.840989000	-0.227460000
C	-0.011732000	-3.549523000	1.005872000
C	-0.059332000	-2.820708000	2.209879000
C	0.137911000	-3.576208000	-1.459450000
C	0.084403000	-4.951909000	-1.434604000
C	-0.003928000	-5.655769000	-0.206982000
C	-0.054260000	-4.975927000	0.981142000
C	0.192637000	-2.822150000	-2.710915000
C	0.291753000	-1.487959000	-2.739050000
H	-0.355412000	0.982313000	-3.704673000
H	0.152918000	-3.388744000	-3.644989000
H	0.355412000	-0.982313000	-3.704673000
H	-0.100390000	-3.361380000	3.159215000
H	-0.130668000	-5.515526000	1.928115000
H	-0.039752000	-6.747197000	-0.215757000
H	0.103498000	-5.507409000	-2.375544000
H	-0.152918000	3.388744000	-3.644989000
H	0.069893000	1.226077000	4.389367000
H	-0.069893000	-1.226077000	4.389367000
H	0.100390000	3.361380000	3.159215000
H	0.130668000	5.515526000	1.928115000
H	0.039752000	6.747197000	-0.215757000
H	-0.103498000	5.507409000	-2.375544000
H	1.481590000	-0.321689000	-1.449347000
H	-1.481590000	0.321689000	-1.449347000

Thermochemistry

Temperature: 298.150 Kelvin; Pressure: 1.00000 Atm.

Zero-point correction = 0.353493 (Hartree/Particle)

Thermal correction to Energy = 0.370606

Thermal correction to Enthalpy = 0.371550

Thermal correction to Gibbs Free Energy = 0.310387

Sum of electronic and zero-point Energies= −1075.190168

Sum of electronic and thermal Energies = −1075.173055

Sum of electronic and thermal Enthalpies = −1075.172111

Sum of electronic and thermal Free Energies = −1075.233274

Charge = 0 Multiplicity = 1

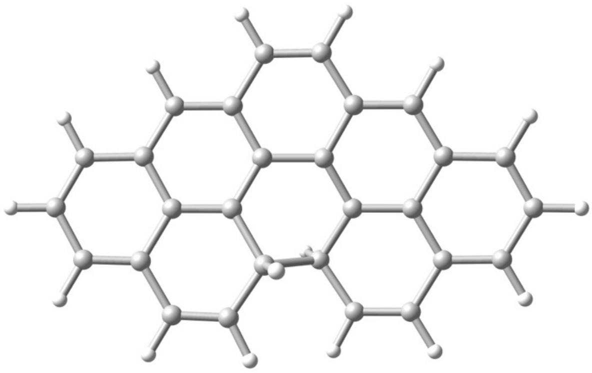

o-**DMC** (broken-symmetry unrestricted singlet)

C	-0.470893000	-5.610203000	-0.206858000
C	0.008665000	-5.009370000	-1.353480000
C	0.394221000	-3.642273000	-1.348090000
C	0.222473000	-2.867648000	-0.168157000
C	-0.222641000	-3.521915000	1.026572000
C	-0.561648000	-4.878720000	0.985454000
C	-0.222473000	-2.779868000	2.246873000
C	-0.023725000	-1.412643000	2.267567000
C	0.136058000	-0.690960000	1.021821000
C	0.549566000	-1.471466000	-0.151388000
C	-0.136058000	0.690960000	1.021821000
C	0.023725000	1.412643000	2.267567000
C	0.046882000	0.674908000	3.506533000
C	-0.046882000	-0.674908000	3.506533000
C	0.994299000	-3.026891000	-2.474153000
C	1.479253000	-1.744959000	-2.376769000
C	1.302206000	-0.948609000	-1.220894000
C	-0.549566000	1.471466000	-0.151388000
C	-0.222473000	2.867648000	-0.168157000
C	0.222641000	3.521915000	1.026572000
C	0.222473000	2.779868000	2.246873000
C	-0.394221000	3.642273000	-1.348090000
C	-0.008665000	5.009370000	-1.353480000
C	0.470893000	5.610203000	-0.206858000

Thermochemistry

Temperature: 298.150 Kelvin; Pressure: 1.00000 Atm.

Zero-point correction = 0.405024 (Hartree/Particle)

Thermal correction to Energy = 0.425832

Thermal correction to Enthalpy = 0.426776

Thermal correction to Gibbs Free Energy = 0.358470

Sum of electronic and zero-point Energies= −1153.663083

Sum of electronic and thermal Energies = −1153.642274

Sum of electronic and thermal Enthalpies = −1153.641330

Sum of electronic and thermal Free Energies = −1153.709636

Charge = 0 Multiplicity = 1

C	0.561648000	4.878720000	0.985454000
C	-0.994299000	3.026891000	-2.474153000
C	-1.479253000	1.744959000	-2.376769000
C	-1.302206000	0.948609000	-1.220894000
C	1.997038000	0.387927000	-1.213802000
H	2.027760000	-1.313894000	-3.218569000
H	-1.124476000	3.602277000	-3.393803000
H	-2.027760000	1.313894000	-3.218569000
C	-1.997038000	-0.387927000	-1.213802000
H	0.417280000	3.307430000	3.184285000
H	0.889574000	5.370693000	1.904364000
H	0.754336000	6.664721000	-0.218432000
H	-0.121039000	5.583901000	-2.276015000
H	1.124476000	-3.602277000	-3.393803000
H	-0.106883000	-1.235177000	4.442547000
H	0.106883000	1.235177000	4.442547000
H	-0.417280000	-3.307430000	3.184285000
H	-0.889574000	-5.370693000	1.904364000
H	-0.754336000	-6.664721000	-0.218432000
H	0.121039000	-5.583901000	-2.276015000
H	2.992938000	0.278122000	-1.668665000
H	1.450316000	1.140771000	-1.801842000
H	2.124571000	0.789618000	-0.201241000
H	-2.992938000	-0.278122000	-1.668665000
H	-1.450316000	-1.140771000	-1.801842000
H	-2.124571000	-0.789618000	-0.201241000

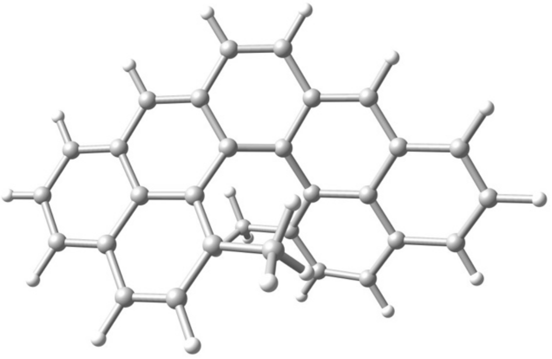

c-DMC

Thermochemistry

Temperature: 298.150 Kelvin; Pressure: 1.00000 Atm.

Zero-point correction = 0.409281 (Hartree/Particle)

Thermal correction to Energy = 0.429258

Thermal correction to Enthalpy = 0.430203

Thermal correction to Gibbs Free Energy = 0.364071

Sum of electronic and zero-point Energies= −1153.689614

Sum of electronic and thermal Energies = −1153.669637

Sum of electronic and thermal Enthalpies = −1153.668693

Sum of electronic and thermal Free Energies = −1153.734824

Charge = 0 Multiplicity = 1

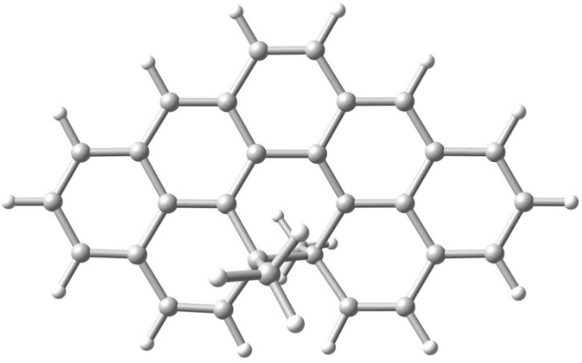

C	-0.000461000	5.664770000	-0.064325000
C	-0.022101000	4.964864000	-1.297057000
C	-0.065702000	3.589075000	-1.330154000
C	-0.090109000	2.845871000	-0.102642000
C	-0.024673000	3.551580000	1.135849000
C	0.005343000	4.978532000	1.120805000
C	0.000550000	2.818915000	2.336559000
C	0.011728000	1.432964000	2.330373000
C	-0.024673000	0.730132000	1.086076000
C	-0.135380000	1.431692000	-0.111997000
C	0.024673000	-0.730132000	1.086076000
C	-0.011728000	-1.432964000	2.330373000
C	-0.017569000	-0.674342000	3.566566000
C	0.017569000	0.674342000	3.566566000
C	0.135380000	-1.431692000	-0.111997000
C	0.416402000	-0.661679000	-1.399508000
C	-0.416402000	0.661679000	-1.399508000
C	-0.135839000	1.508607000	-2.618774000
C	-0.027684000	2.840721000	-2.584238000
C	0.090109000	-2.845871000	-0.102642000
C	0.024673000	-3.551580000	1.135849000
C	-0.000550000	-2.818915000	2.336559000
C	0.065702000	-3.589075000	-1.330154000
C	0.022101000	-4.964864000	-1.297057000
C	0.000461000	-5.664770000	-0.064325000
C	-0.005343000	-4.978532000	1.120805000
C	0.027684000	-2.840721000	-2.584238000
C	0.135839000	-1.508607000	-2.618774000
C	-1.932626000	0.335590000	-1.427627000
H	-0.115287000	1.004950000	-3.587731000
H	-0.099608000	-3.407208000	-3.510428000
H	0.115287000	-1.004950000	-3.587731000
C	1.932626000	-0.335590000	-1.427627000
H	-0.015880000	-3.354733000	3.289359000
H	-0.039590000	-5.512643000	2.073352000
H	-0.026421000	-6.756470000	-0.066595000
H	-0.008036000	-5.523358000	-2.235966000
H	0.099608000	3.407208000	-3.510428000
H	0.032308000	1.228386000	4.508247000
H	-0.032308000	-1.228386000	4.508247000
H	0.015880000	3.354733000	3.289359000
H	0.039590000	5.512643000	2.073352000
H	0.026421000	6.756470000	-0.066595000

H	0.008036000	5.523358000	-2.235966000
H	-2.197162000	-0.202484000	-2.350378000
H	-2.218113000	-0.292117000	-0.570894000
H	-2.514255000	1.267082000	-1.388403000
H	2.197162000	0.202484000	-2.350378000
H	2.218113000	0.292117000	-0.570894000
H	2.514255000	-1.267082000	-1.388403000

o-**DMNC** (broken-symmetry unrestricted singlet)

C	2.254364000	-0.714184000	0.123880000
C	2.254250000	0.714378000	-0.124058000
C	3.479466000	-1.413049000	-0.032641000
C	3.479248000	1.413428000	0.032453000
C	4.699715000	0.682648000	0.062689000
C	4.699815000	-0.682075000	-0.063021000
C	3.457420000	-2.837592000	-0.161090000
C	1.084906000	-1.460425000	0.496484000
C	1.044881000	-2.838230000	0.142517000
C	2.273233000	-3.511500000	-0.170927000
C	-0.195116000	-3.493889000	0.053022000
C	-1.402493000	-2.817003000	0.327785000
C	-1.338392000	-1.524469000	0.949883000
C	-0.077164000	-0.893418000	1.171893000
C	-2.545002000	-0.905844000	1.382246000
C	-2.457570000	0.293752000	2.124156000
C	-1.224771000	0.780843000	2.501794000
C	-0.017648000	0.194915000	2.076501000
C	1.084626000	1.460488000	-0.496369000
C	1.044397000	2.838222000	-0.142164000
C	2.272668000	3.511647000	0.171259000
C	3.456973000	2.837946000	0.161139000
C	-0.077353000	0.893429000	-1.171859000
C	-1.338674000	1.524269000	-0.949828000
C	-1.402982000	2.816646000	-0.327425000
C	-0.195708000	3.493658000	-0.052492000
C	-2.545154000	0.905605000	-1.382492000
C	-3.790925000	1.512927000	-1.075852000
C	-3.842023000	2.733461000	-0.420816000
C	-2.457481000	-0.293774000	-2.124736000
C	-1.224571000	-0.780637000	-2.502323000
C	-0.017576000	-0.194702000	-2.076667000
C	-3.790667000	-1.513390000	1.075617000
C	-3.841554000	-2.734098000	0.420892000
C	-2.663462000	-3.397025000	0.073427000
C	1.255618000	0.713617000	2.696099000
C	1.255938000	-0.713170000	-2.695971000
H	5.640319000	1.233695000	0.135801000
H	5.640502000	-1.232979000	-0.136144000
H	4.401656000	-3.365413000	-0.314807000
H	2.242414000	-4.584254000	-0.375829000
H	-0.226789000	-4.532614000	-0.285926000
H	-3.373868000	0.797675000	2.439080000
H	-1.172099000	1.651755000	3.160421000
H	2.241689000	4.584362000	0.376346000
H	4.401135000	3.365900000	0.314854000
H	-0.227538000	4.532324000	0.286621000
H	-4.710328000	1.006950000	-1.379469000
H	-4.807681000	3.193233000	-0.199153000
H	-3.373678000	-0.797736000	-2.439888000
H	-1.171706000	-1.651406000	-3.161123000
H	-4.710164000	-1.007438000	1.378991000
H	-4.807135000	-3.194030000	0.199228000
H	1.083086000	0.898425000	3.767041000
H	1.582136000	1.666225000	2.252437000
H	2.085464000	0.002441000	2.604112000
H	1.083813000	-0.897631000	-3.767035000
H	1.582388000	-1.665901000	-2.252527000
H	2.085702000	-0.001961000	-2.603416000
H	-2.705826000	-4.381628000	-0.398424000
C	-2.664047000	3.396441000	-0.073043000
H	-2.706591000	4.380936000	0.399018000

Thermochemistry

Temperature: 298.150 Kelvin; Pressure: 1.00000 Atm.

Zero-point correction = 0.498623 (Hartree/Particle)

Thermal correction to Energy = 0.524538

Thermal correction to Enthalpy = 0.525482

Thermal correction to Gibbs Free Energy = 0.447064

Sum of electronic and zero-point Energies= −1460.531055

Sum of electronic and thermal Energies = −1460.505140

Sum of electronic and thermal Enthalpies = −1460.504196

Sum of electronic and thermal Free Energies = −1460.582614

Charge = 0 Multiplicity = 1

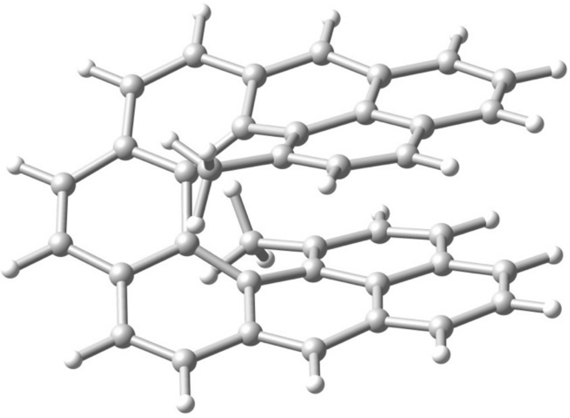

C	2.380429000	0.276972000	-0.069067000
C	1.960201000	-1.059977000	-0.219636000
C	3.719982000	0.576672000	0.254097000
C	2.928376000	-2.060075000	-0.326350000
C	4.283103000	-1.747940000	-0.073933000
C	4.666017000	-0.467703000	0.263570000
C	4.062871000	1.929784000	0.624297000
C	1.419109000	1.354777000	-0.204124000
C	1.768710000	2.666214000	0.231513000
C	3.127564000	2.909774000	0.661649000
C	0.810975000	3.672606000	0.187830000
C	-0.482350000	3.418621000	-0.296639000
C	-0.793143000	2.122113000	-0.815305000
C	0.179597000	1.102327000	-0.789937000
C	-2.109252000	1.870993000	-1.333311000
C	-2.391685000	0.543858000	-1.867455000
C	-1.479461000	-0.433885000	-1.873489000
C	-0.055035000	-0.269892000	-1.412725000
C	0.461405000	-1.351688000	-0.326971000
C	0.182346000	-2.786792000	-0.766963000
C	1.274677000	-3.689282000	-1.089995000
C	2.552153000	-3.387404000	-0.801930000
C	-0.363798000	-1.089585000	0.964520000
C	-1.746405000	-1.468738000	0.850459000
C	-2.140874000	-2.510396000	-0.051838000
C	-1.082971000	-3.258509000	-0.708514000
C	-2.736959000	-0.894989000	1.685660000
C	-4.102447000	-1.227550000	1.486343000
C	-4.466458000	-2.183907000	0.567231000
C	-3.473614000	-2.861133000	-0.167754000
C	-2.314701000	-0.031751000	2.728491000
C	-0.979136000	0.118892000	2.970014000
C	0.031936000	-0.431966000	2.125289000
C	-3.052324000	2.872113000	-1.313780000
C	-2.742510000	4.158372000	-0.799465000
C	-1.494315000	4.428264000	-0.308840000
C	0.776005000	-0.398941000	-2.717947000
C	1.440176000	-0.273723000	2.654917000
H	5.028002000	-2.544770000	-0.139787000
H	5.706976000	-0.247761000	0.512138000
H	5.096119000	2.141177000	0.910542000
H	3.395626000	3.919083000	0.983257000
H	1.067645000	4.679577000	0.527542000
H	-3.386821000	0.362332000	-2.280705000
H	-1.725023000	-1.388688000	-2.338757000
H	1.012229000	-4.677334000	-1.476662000
H	3.347211000	-4.118960000	-0.964646000
H	-1.304130000	-4.272920000	-1.052944000
H	-4.857929000	-0.731891000	2.101032000
H	-5.517335000	-2.449265000	0.434545000
H	-3.058508000	0.447351000	3.369164000
H	-0.651672000	0.699352000	3.836694000
H	-4.054438000	2.671893000	-1.700848000
H	-3.510741000	4.934744000	-0.799500000
H	1.844035000	-0.202587000	-2.561447000
H	0.399610000	0.320872000	-3.457713000
H	0.666821000	-1.411691000	-3.135693000
H	1.846342000	0.733154000	2.474839000
H	2.142763000	-1.006354000	2.246967000
H	1.410374000	-0.416785000	3.745488000
H	-1.251845000	5.418344000	0.084445000
H	-3.754486000	-3.678024000	-0.837431000

c-DMNC

Thermochemistry

Temperature: 298.150 Kelvin; Pressure: 1.00000 Atm.
Zero-point correction = 0.503732 (Hartree/Particle)
Thermal correction to Energy = 0.528851
Thermal correction to Enthalpy = 0.529795
Thermal correction to Gibbs Free Energy = 0.452585
Sum of electronic and zero-point Energies= −1460.522945
Sum of electronic and thermal Energies = −1460.497825
Sum of electronic and thermal Enthalpies = −1460.496881
Sum of electronic and thermal Free Energies = −1460.574091
Charge = 0 Multiplicity = 1

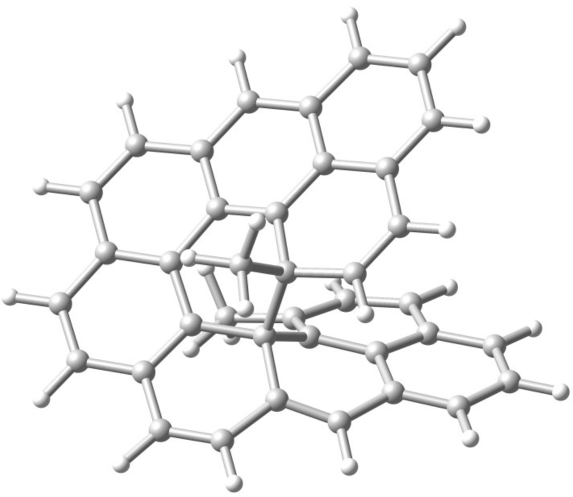

o-**DMNC*** (broken-symmetry unrestricted singlet)

C	-1.133781000	2.927224000	0.359165000
C	-1.075398000	1.628360000	0.969278000
C	0.084063000	3.554839000	0.031482000
C	1.312788000	2.865525000	0.077647000
C	1.337486000	1.493586000	0.435390000
C	0.169479000	0.955097000	1.109575000
C	2.545581000	3.519498000	-0.258445000
C	2.507501000	0.719418000	0.097585000
C	3.720420000	2.832780000	-0.242441000
C	3.733255000	1.409695000	-0.075343000
C	2.507540000	-0.719291000	-0.097576000
C	3.733330000	-1.409504000	0.075351000
C	4.954968000	-0.679053000	0.083347000
C	4.954932000	0.679310000	-0.083332000
C	1.337567000	-1.493519000	-0.435390000
C	3.720572000	-2.832591000	0.242437000
C	2.545771000	-3.519374000	0.258425000
C	1.312943000	-2.865464000	-0.077667000
C	0.169532000	-0.955085000	-1.109571000
C	0.084257000	-3.554849000	-0.031519000
C	-1.133621000	-2.927294000	-0.359191000
C	-1.075309000	-1.628416000	-0.969280000
C	-2.269057000	1.010965000	1.445840000
C	-2.184279000	-0.210944000	2.150022000
C	-0.948449000	-0.777454000	2.415809000
C	0.216161000	-0.202886000	1.907366000
C	0.216150000	0.202909000	-1.907348000
C	-0.948494000	0.777433000	-2.415765000
C	-2.184294000	0.210860000	-2.149968000
C	-2.269004000	-1.011067000	-1.445811000
C	-3.509605000	1.653863000	1.197928000
C	-3.558059000	2.881544000	0.557376000
C	-2.398061000	3.551581000	0.150067000
C	-3.509517000	-1.654034000	-1.197903000
C	-2.397868000	-3.551720000	-0.150096000
C	-3.557904000	-2.881732000	-0.557379000
H	0.090737000	4.592833000	-0.306109000
H	2.527132000	4.589466000	-0.479141000
H	4.669318000	3.346804000	-0.413358000
H	5.895081000	-1.228501000	0.172878000
H	5.895016000	1.228808000	-0.172861000
H	4.669497000	-3.346564000	0.413354000
H	2.527379000	-4.589345000	0.479109000
H	0.090988000	-4.592847000	0.306056000
H	-3.102144000	-0.685789000	2.503727000
H	-0.880706000	-1.692288000	3.007835000
H	1.177719000	-0.668969000	2.119978000
H	1.177683000	0.669042000	-2.119964000
H	-0.880802000	1.692279000	-3.007777000
H	-3.102186000	0.685666000	-2.503656000
H	-4.431205000	1.168911000	1.527980000
H	-4.528140000	3.353835000	0.380814000
H	-4.431145000	-1.169120000	-1.527934000
H	-4.527959000	-3.354075000	-0.380817000
C	-2.487767000	4.903610000	-0.500141000
H	-3.534007000	5.214547000	-0.623576000
H	-1.979253000	5.675928000	0.100129000
H	-2.014271000	4.905467000	-1.495201000
C	-2.487502000	-4.903766000	0.500086000
H	-3.533726000	-5.214748000	0.623542000
H	-1.978974000	-5.676051000	-0.100213000
H	-2.013981000	-4.905626000	1.495134000

Thermochemistry

Temperature: 298.150 Kelvin; Pressure: 1.00000 Atm.

Zero-point correction = 0.498515 (Hartree/Particle)

Thermal correction to Energy = 0.524889

Thermal correction to Enthalpy = 0.525833

Thermal correction to Gibbs Free Energy = 0.445052

Sum of electronic and zero-point Energies= −1460.537611

Sum of electronic and thermal Energies = −1460.511238

Sum of electronic and thermal Enthalpies = −1460.510293

Sum of electronic and thermal Free Energies = −1460.591074

Charge = 0 Multiplicity = 1

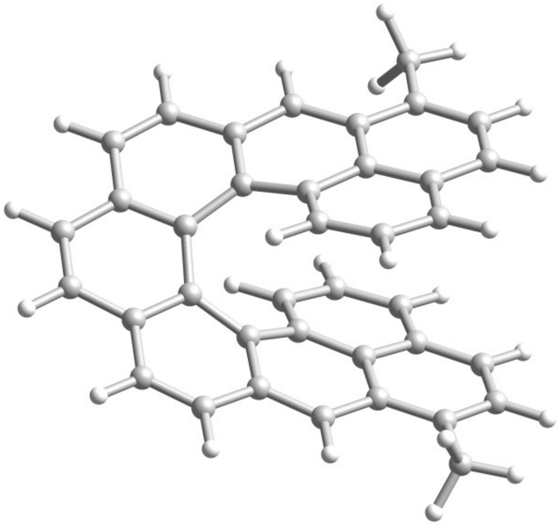

C	1.537922000	2.079089000	-0.039762000
C	0.182365000	2.420778000	-0.224575000
C	2.487312000	3.067434000	0.302992000
C	-0.159875000	3.769368000	-0.355616000
C	0.809436000	4.760685000	-0.088144000
C	2.091338000	4.419261000	0.290036000
C	3.820051000	2.651979000	0.676986000
C	1.966493000	0.701574000	-0.191456000
C	3.267694000	0.315348000	0.233089000
C	4.176986000	1.344464000	0.686108000
C	3.636051000	-1.024244000	0.155468000
C	2.759486000	-1.996440000	-0.356941000
C	1.488162000	-1.576355000	-0.866050000
C	1.115103000	-0.220059000	-0.798504000
C	0.589345000	-2.546303000	-1.428198000
C	-0.689312000	-2.084153000	-1.959269000
C	-1.056017000	-0.797819000	-1.932892000
C	-0.188761000	0.290471000	-1.380023000
C	-0.849963000	1.297694000	-0.339272000
C	-2.208738000	1.800982000	-0.810320000
C	-2.386810000	3.192725000	-1.187723000
C	-1.469760000	4.130188000	-0.887907000
C	-1.068750000	0.508514000	0.964202000
C	-2.177616000	-0.391991000	0.986907000
C	-3.251674000	-0.252403000	0.048214000
C	-3.284789000	0.986098000	-0.715426000
C	-2.246740000	-1.403803000	1.979534000
C	-3.303429000	-2.349099000	1.911819000
C	-4.272548000	-2.251386000	0.943569000
C	-4.285587000	-1.181535000	0.015067000
C	-1.271000000	-1.430629000	3.010698000
C	-0.292445000	-0.471361000	3.052990000
C	-0.186301000	0.492676000	2.022918000
C	0.960914000	-3.867677000	-1.452374000
C	2.216672000	-4.283159000	-0.941045000
C	3.114224000	-3.393453000	-0.405933000
H	0.073348000	0.941493000	-2.239391000
H	0.525781000	5.812217000	-0.177823000
H	2.817424000	5.194169000	0.547255000
H	4.534739000	3.421554000	0.979061000
H	5.179841000	1.047216000	1.002472000
H	4.633561000	-1.312149000	0.492166000
H	-1.351562000	-2.831835000	-2.403210000
H	-2.003996000	-0.495639000	-2.379192000
H	-3.351249000	3.479238000	-1.614997000
H	-1.670900000	5.186183000	-1.083687000
H	-4.250352000	1.341619000	-1.085088000
H	-3.338597000	-3.156283000	2.647613000
H	-5.074547000	-2.993072000	0.904688000
H	-1.326857000	-2.208800000	3.775695000
H	0.438093000	-0.463022000	3.864376000
H	0.632945000	1.208867000	2.067165000
H	0.279417000	-4.611969000	-1.871703000
H	2.474596000	-5.344817000	-0.978145000
C	-5.419737000	-1.053943000	-0.968172000
H	-6.062665000	-1.944536000	-0.948963000
H	-6.056351000	-0.183625000	-0.738043000
H	-5.052205000	-0.923693000	-1.997390000
C	4.443232000	-3.857535000	0.123213000
H	5.280172000	-3.389371000	-0.419682000
H	4.564912000	-3.603231000	1.188305000
H	4.546844000	-4.946403000	0.023265000

c-DMNC*

Thermochemistry

Temperature: 298.150 Kelvin; Pressure: 1.00000 Atm.

Zero-point correction = 0.503555 (Hartree/Particle)

Thermal correction to Energy = 0.529157

Thermal correction to Enthalpy = 0.530102

Thermal correction to Gibbs Free Energy = 0.450830

Sum of electronic and zero-point Energies= −1460.537626

Sum of electronic and thermal Energies = −1460.512024

Sum of electronic and thermal Enthalpies = −1460.511079

Sum of electronic and thermal Free Energies = −1460.590351

Charge = 0 Multiplicity = 1

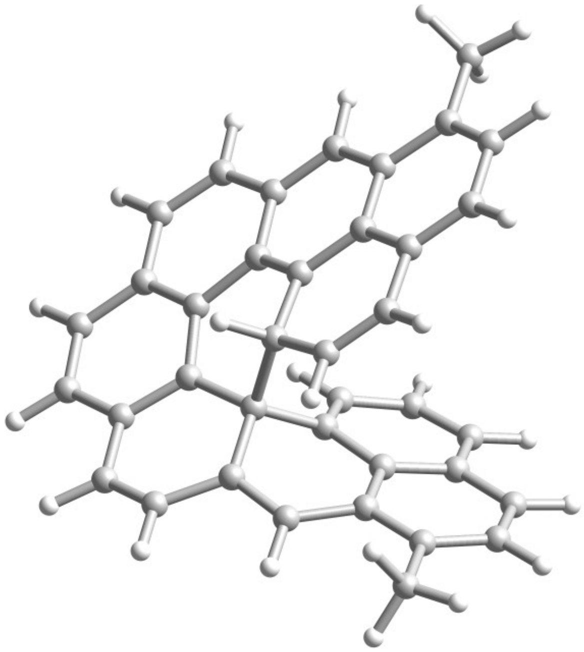

References

1 G. R. Fulmer, A. J. M. Miller, N. H. Sherden, H. E. Gottlieb, A. Nudelman, B. M. Stoltz, J. E. Bercaw, K. I. Goldberg, *Organometallics,* 2010, **29**, 2176–2179.

2 B. G. Das, A. Chirila, M. Tromp, J. N. H. Reek, B. de Bruin, *J. Am. Chem. Soc.,* **2016**, *138,* 8968–8975.

3 X. Liu, Z. Wang, Q. Chen, M. He, L. Wang, *Appl. Organomet. Chem.,* 2018, **32**, e4039.

4 R. C. Clark, J. S. Reid, *Acta Crystallogr. Sect. A,* 1995, **51**, 887–897.

5 CrysAlisPro. Rigaku Oxford Diffraction Ltd: Yarnton, England 2019.

6 O. v Dolomanov, L. J. Bourhis, R. J. Gildea, J. A. K. Howard, H. Puschmann *J. Appl. Crystallogr.,* 2009, **42**, 339–341.

7 G. M. Sheldrick, SHELXT – Integrated Space-Group and Crystal-Structure Determination. *Acta Crystallogr. Sect. A,* 2015, **71**, 3–8.

8 G. M. Sheldrick, *Acta Crystallogr. Sect. C,* 2015, **71**, 3–8.

9 A. L. Spek, *Acta Crystallogr. Sect. D,* 2009, **65**, 148–155.

10 M. J. Frisch, G. W. Trucks, H. B. Schlegel, G. E. Scuseria, M. A. Robb, J. R. Cheeseman, G. Scalmani, V. Barone, G. A. Petersson, H. Nakatsuji, X. Li, M. Caricato, A. v. Marenich, J. Bloino, B. G. Janesko, R. Gomperts, B. Mennucci, H. P. Hratchian, J. v. Ortiz, A. F. Izmaylov, J. L. Sonnenberg, D. Williams-Young, F. Ding, F. Lipparini, F. Egidi, J. Goings, B. Peng, A. Petrone, T. Henderson, D. Ranasinghe, V. G. Zakrzewski, J. Gao, N. Rega, G. Zheng, W. Liang, M. Hada, M. Ehara, K. Toyota, R. Fukuda, J. Hasegawa, M. Ishida, T. Nakajima, Y. Honda, O. Kitao, H. Nakai, T. Vreven, K. Throssell, J. A. Jr. Montgomery, J. E. Peralta, F. Ogliaro, M. J. Bearpark, J. J. Heyd, E. N. Brothers, K. N. Kudin, V. N. Staroverov, T. A. Keith, R. Kobayashi, J. Normand, K. Raghavachari, A. P. Rendell, J. C. Burant, S. S. Iyengar, J. Tomasi, M. Cossi, J. M. Millam, M. Klene, C. Adamo, R. Cammi, J. W. Ochterski, R. L. Martin, K. Morokuma, O. Farkas, J. B. Foresman, D. J. Fox, Gaussian 16 Rev. C.01. Wallingford, CT 2016.

11 F. Neese, F. Wennmohs, U. Becker, C. Riplinger, *J. Chem. Phys.,* 2020, **152**, 224108.

12 WinSim EPR simulator, https://www.niehs.nih.gov/research/resources/software/tox-pharm/tools/index.cfm, retrieved 02.02.2023.

13 K. Yamaguchi, *Chem. Phys. Lett.,* 1975, **33**, 330–335.

14 P. v. R. Schleyer, C. Maerker, A. Dransfeld, H. Jiao, N. J. R. van Eikema Hommes, *J. Am. Chem. Soc.,* 1996, **118**, 6317–6318.

15 D. Geuenich, K. Hess, F. Köhler, R. Herges, *Chem. Rev.,* 2005, **105**, 3758–3772.

16 R. Herges, D. Geuenich, *J. Phys. Chem. A,* 2001, **105**, 3214–3220.

17 D. Čavlović, D. Häussinger, O. Blacque, P. Ravat, M. Juríček, *JACS Au,* 2022, **2**, 1616–1626.
